# Supplementary figures and images for: Suppression of mechanical hypersensitivity and change in the expression of the dopamine D2 receptor by administration of anti-CGRP antibody into the trigeminal ganglion in trigeminal neuropathic pain model rats
Source: PLoS One. 2025 May 14;20(5):e0323810. doi: 10.1371/journal.pone.0323810 (PMC12077688; doi:10.1371/journal.pone.0323810)

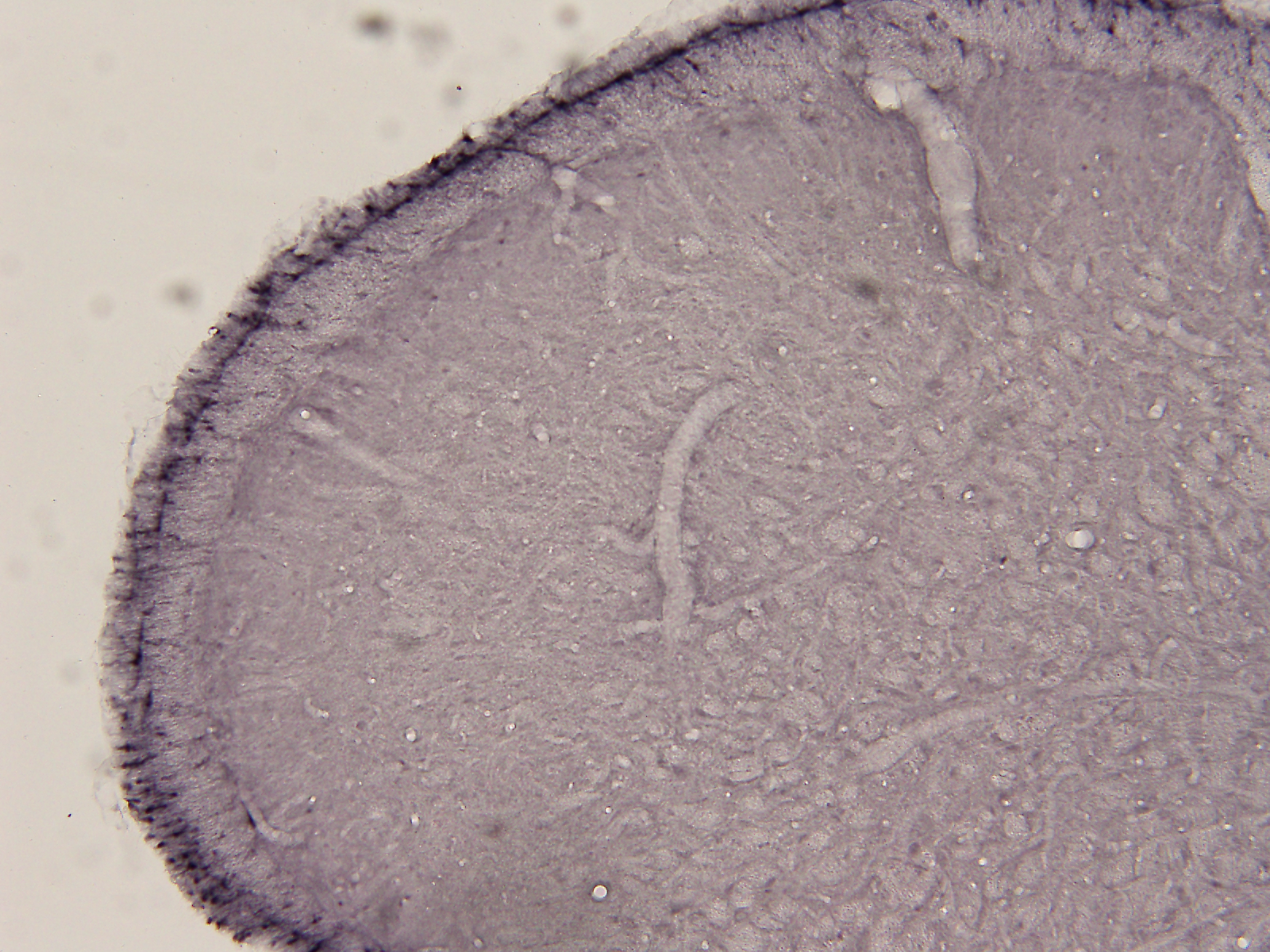

Supplement: S1 Data — S1 Fig. Photomicrograph of phosphorylated extracellular signal-regulated kinase (pERK)-immunoreactive (-IR) neurons in the trigeminal spinal subnucleus caudalis (Vc) of a sham rat. S2 Fig. Photomicrograph of pERK-IR neurons in the Vc of a chronic constriction injury of the infraorbital nerve (ION-CCI) rat. S3 Fig. Photomicrograph of pERK-IR neurons in the Vc of an ION-CCI rat receiving a vehicle. S4 Fig. Photomicrograph of pERK-IR neurons in the Vc of an ION-CCI rat receiving calcitonin gene-related peptide (CGRP). S5 Fig. Photomicrograph of pERK-IR neurons in the Vc of an ION-CCI rat receiving control immunogloublin G (IgG). S6 Fig. Photomicrograph of pERK-IR neurons in the Vc of an ION-CCI rat receiving an anti-CGRP antibody. S7 Fig. High magnification photomicrograph of pERK-IR neurons in the Vc of a sham rat. S8 Fig. High magnification photomicrograph of pERK-IR neurons in the Vc of an ION-CCI rat. S9 Fig. High magnification photomicrograph of pERK-IR neurons in the Vc of an ION-CCI rat receiving a vehicle. S10 Fig. High magnification photomicrograph of pERK-IR neurons in the Vc of an ION-CCI rat receiving CGRP. S11 Fig. High magnification photomicrograph of pERK-IR neurons in the Vc of an ION-CCI rat receiving control IgG. S12 Fig. High magnification photomicrograph of pERK-IR neurons in the Vc of an ION-CCI rat receiving an anti-CGRP antibody. S13 Fig. Immunofluorescent image of pERK in the Vc. S14 Fig. Immunofluorescent image of neuronal nuclei (NeuN) in the Vc. S15 Fig. Merged image of pERK and NeuN in the Vc. S16 Fig. Immunofluorescent image of pERK in the Vc. S17 Fig. Immunofluorescent image of dopamineD2 receptor (D2 receptor) in the Vc. S18 Fig. Merged image of pERK and D2 receptor in the Vc. S19 Fig. Immunofluorescent image of receptor activity modifying protein 1 (RAMP1) in the Vc. S20 Fig. Immunofluorescent image of D2 receptor in the Vc. S21 Fig. Merged image of RAMP1 and D2 receptor in the Vc. S22 Fig. Photomicrograph of CGRP-IR neurons in the trige [file pone.0323810.s001.zip › supplementary/Fig S1.TIF]

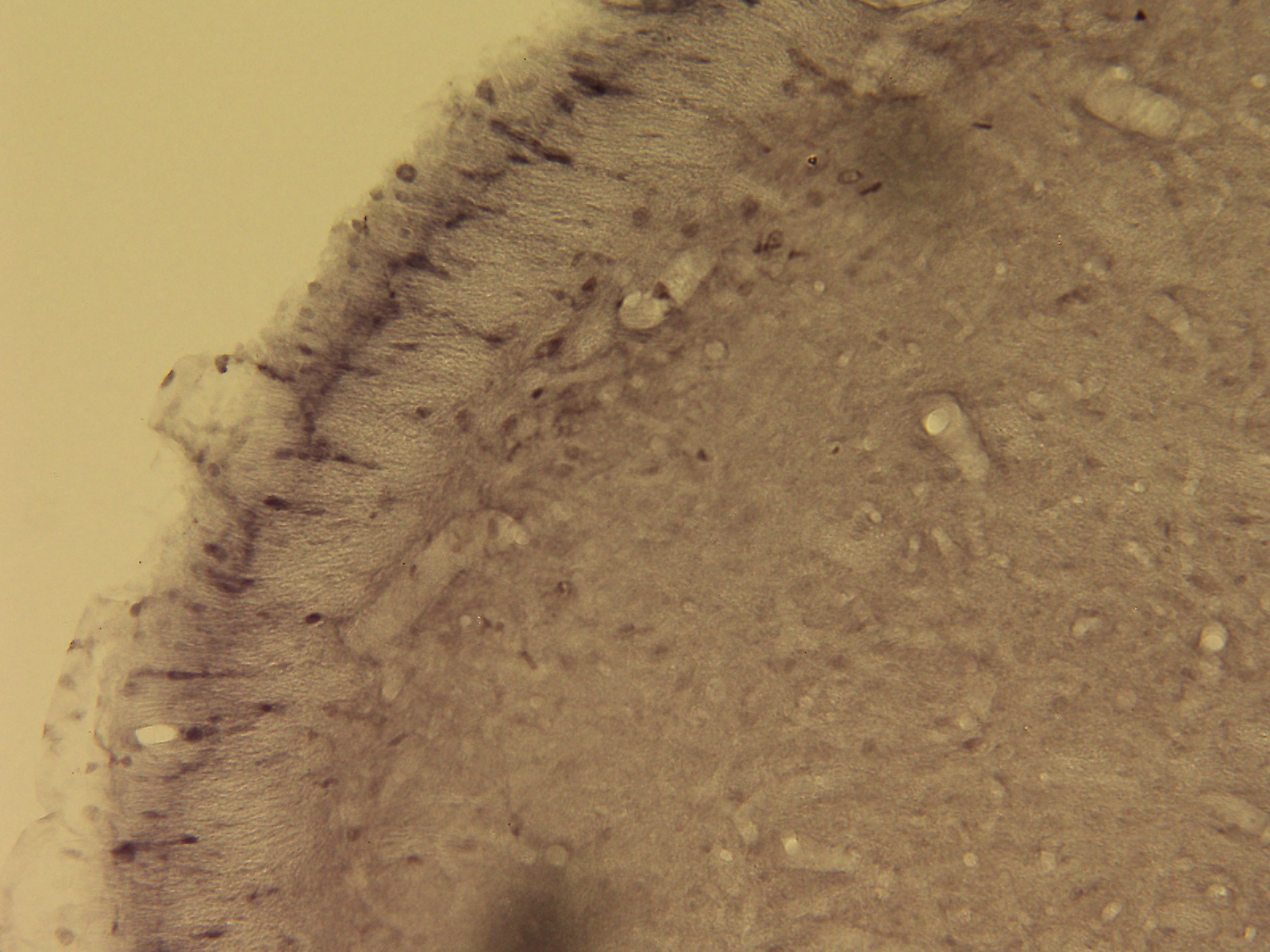

Supplement: S1 Data — S1 Fig. Photomicrograph of phosphorylated extracellular signal-regulated kinase (pERK)-immunoreactive (-IR) neurons in the trigeminal spinal subnucleus caudalis (Vc) of a sham rat. S2 Fig. Photomicrograph of pERK-IR neurons in the Vc of a chronic constriction injury of the infraorbital nerve (ION-CCI) rat. S3 Fig. Photomicrograph of pERK-IR neurons in the Vc of an ION-CCI rat receiving a vehicle. S4 Fig. Photomicrograph of pERK-IR neurons in the Vc of an ION-CCI rat receiving calcitonin gene-related peptide (CGRP). S5 Fig. Photomicrograph of pERK-IR neurons in the Vc of an ION-CCI rat receiving control immunogloublin G (IgG). S6 Fig. Photomicrograph of pERK-IR neurons in the Vc of an ION-CCI rat receiving an anti-CGRP antibody. S7 Fig. High magnification photomicrograph of pERK-IR neurons in the Vc of a sham rat. S8 Fig. High magnification photomicrograph of pERK-IR neurons in the Vc of an ION-CCI rat. S9 Fig. High magnification photomicrograph of pERK-IR neurons in the Vc of an ION-CCI rat receiving a vehicle. S10 Fig. High magnification photomicrograph of pERK-IR neurons in the Vc of an ION-CCI rat receiving CGRP. S11 Fig. High magnification photomicrograph of pERK-IR neurons in the Vc of an ION-CCI rat receiving control IgG. S12 Fig. High magnification photomicrograph of pERK-IR neurons in the Vc of an ION-CCI rat receiving an anti-CGRP antibody. S13 Fig. Immunofluorescent image of pERK in the Vc. S14 Fig. Immunofluorescent image of neuronal nuclei (NeuN) in the Vc. S15 Fig. Merged image of pERK and NeuN in the Vc. S16 Fig. Immunofluorescent image of pERK in the Vc. S17 Fig. Immunofluorescent image of dopamineD2 receptor (D2 receptor) in the Vc. S18 Fig. Merged image of pERK and D2 receptor in the Vc. S19 Fig. Immunofluorescent image of receptor activity modifying protein 1 (RAMP1) in the Vc. S20 Fig. Immunofluorescent image of D2 receptor in the Vc. S21 Fig. Merged image of RAMP1 and D2 receptor in the Vc. S22 Fig. Photomicrograph of CGRP-IR neurons in the trige [file pone.0323810.s001.zip › supplementary/Fig S10.TIF]

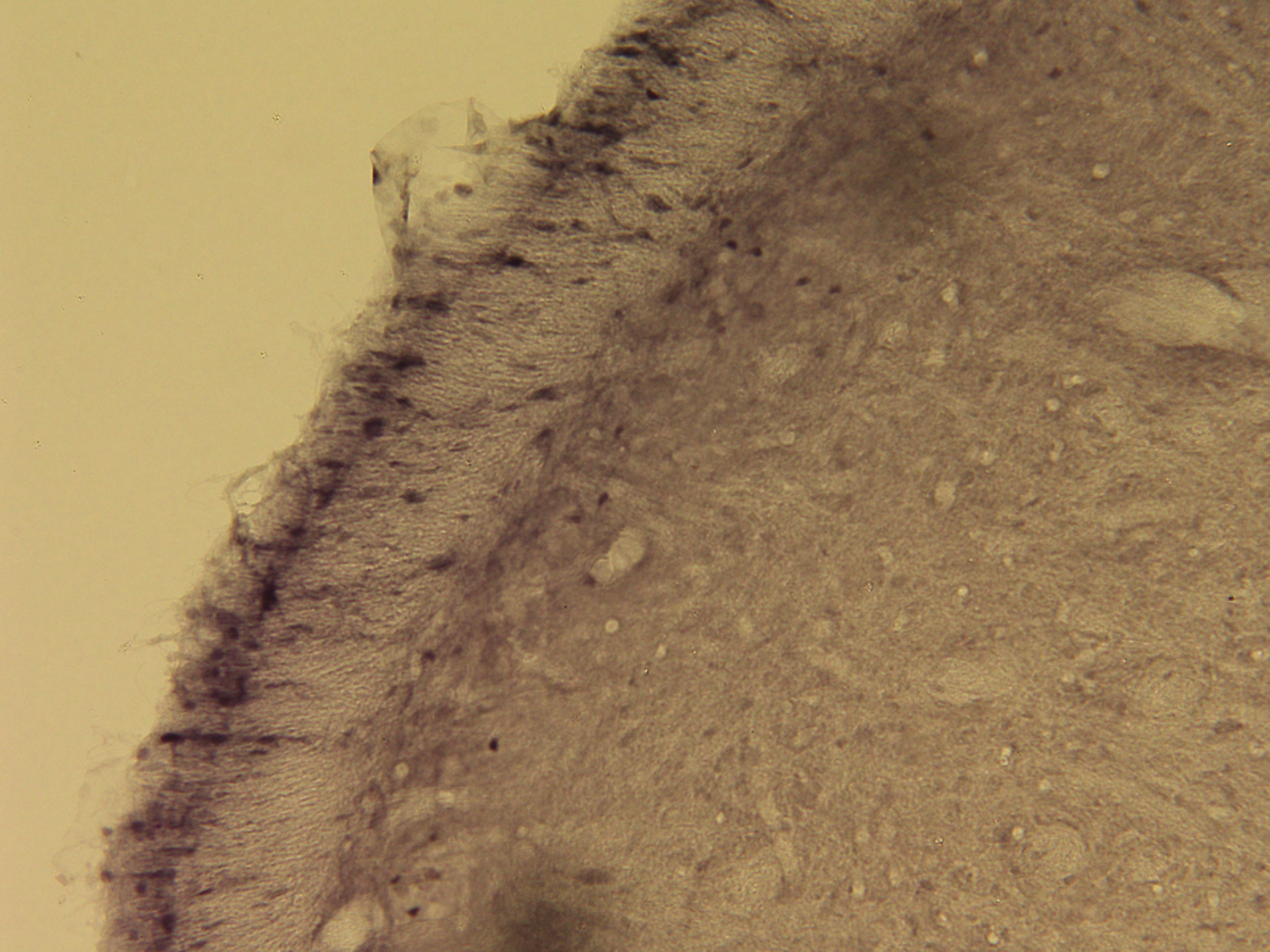

Supplement: S1 Data — S1 Fig. Photomicrograph of phosphorylated extracellular signal-regulated kinase (pERK)-immunoreactive (-IR) neurons in the trigeminal spinal subnucleus caudalis (Vc) of a sham rat. S2 Fig. Photomicrograph of pERK-IR neurons in the Vc of a chronic constriction injury of the infraorbital nerve (ION-CCI) rat. S3 Fig. Photomicrograph of pERK-IR neurons in the Vc of an ION-CCI rat receiving a vehicle. S4 Fig. Photomicrograph of pERK-IR neurons in the Vc of an ION-CCI rat receiving calcitonin gene-related peptide (CGRP). S5 Fig. Photomicrograph of pERK-IR neurons in the Vc of an ION-CCI rat receiving control immunogloublin G (IgG). S6 Fig. Photomicrograph of pERK-IR neurons in the Vc of an ION-CCI rat receiving an anti-CGRP antibody. S7 Fig. High magnification photomicrograph of pERK-IR neurons in the Vc of a sham rat. S8 Fig. High magnification photomicrograph of pERK-IR neurons in the Vc of an ION-CCI rat. S9 Fig. High magnification photomicrograph of pERK-IR neurons in the Vc of an ION-CCI rat receiving a vehicle. S10 Fig. High magnification photomicrograph of pERK-IR neurons in the Vc of an ION-CCI rat receiving CGRP. S11 Fig. High magnification photomicrograph of pERK-IR neurons in the Vc of an ION-CCI rat receiving control IgG. S12 Fig. High magnification photomicrograph of pERK-IR neurons in the Vc of an ION-CCI rat receiving an anti-CGRP antibody. S13 Fig. Immunofluorescent image of pERK in the Vc. S14 Fig. Immunofluorescent image of neuronal nuclei (NeuN) in the Vc. S15 Fig. Merged image of pERK and NeuN in the Vc. S16 Fig. Immunofluorescent image of pERK in the Vc. S17 Fig. Immunofluorescent image of dopamineD2 receptor (D2 receptor) in the Vc. S18 Fig. Merged image of pERK and D2 receptor in the Vc. S19 Fig. Immunofluorescent image of receptor activity modifying protein 1 (RAMP1) in the Vc. S20 Fig. Immunofluorescent image of D2 receptor in the Vc. S21 Fig. Merged image of RAMP1 and D2 receptor in the Vc. S22 Fig. Photomicrograph of CGRP-IR neurons in the trige [file pone.0323810.s001.zip › supplementary/Fig S11.TIF]

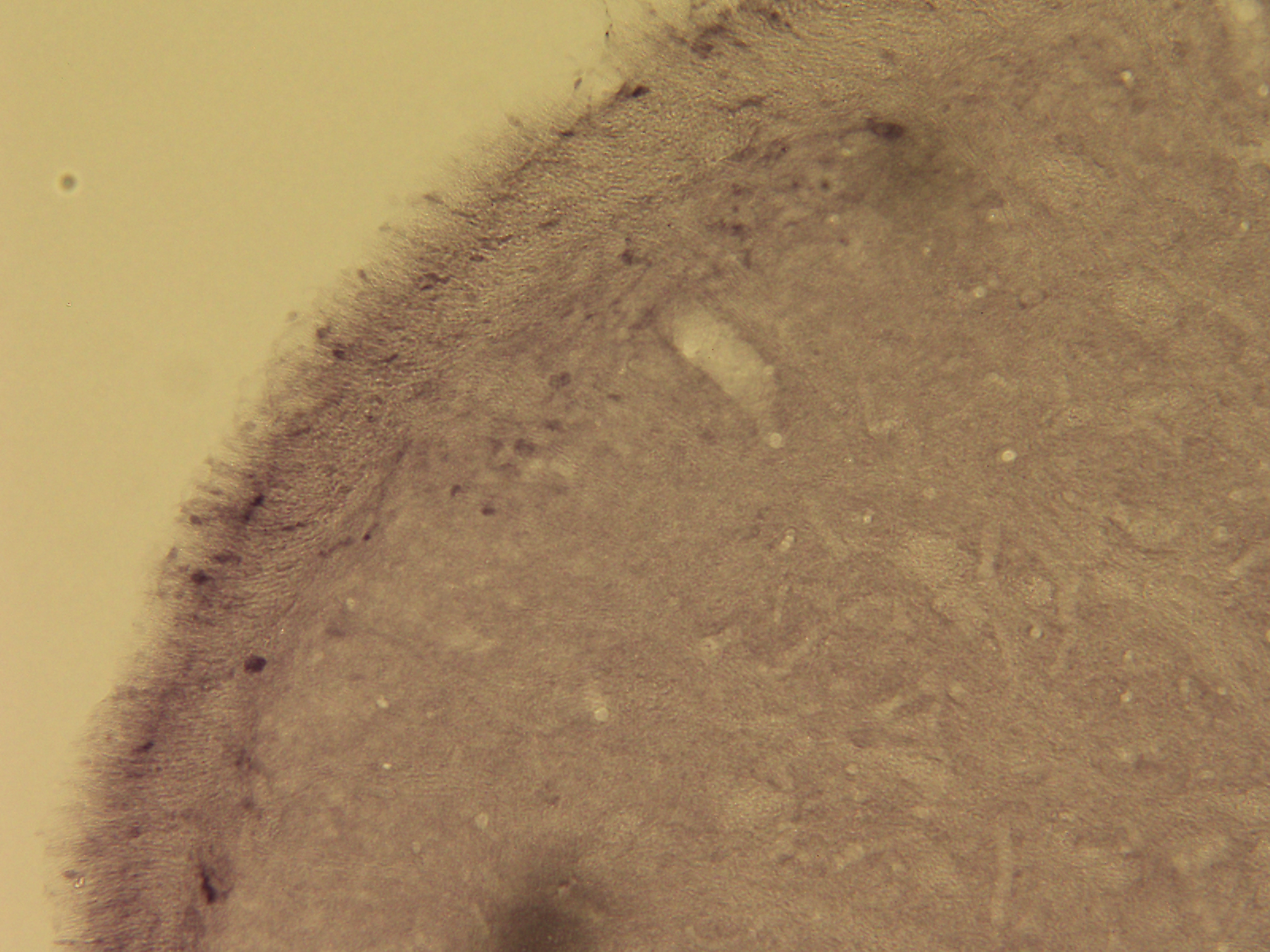

Supplement: S1 Data — S1 Fig. Photomicrograph of phosphorylated extracellular signal-regulated kinase (pERK)-immunoreactive (-IR) neurons in the trigeminal spinal subnucleus caudalis (Vc) of a sham rat. S2 Fig. Photomicrograph of pERK-IR neurons in the Vc of a chronic constriction injury of the infraorbital nerve (ION-CCI) rat. S3 Fig. Photomicrograph of pERK-IR neurons in the Vc of an ION-CCI rat receiving a vehicle. S4 Fig. Photomicrograph of pERK-IR neurons in the Vc of an ION-CCI rat receiving calcitonin gene-related peptide (CGRP). S5 Fig. Photomicrograph of pERK-IR neurons in the Vc of an ION-CCI rat receiving control immunogloublin G (IgG). S6 Fig. Photomicrograph of pERK-IR neurons in the Vc of an ION-CCI rat receiving an anti-CGRP antibody. S7 Fig. High magnification photomicrograph of pERK-IR neurons in the Vc of a sham rat. S8 Fig. High magnification photomicrograph of pERK-IR neurons in the Vc of an ION-CCI rat. S9 Fig. High magnification photomicrograph of pERK-IR neurons in the Vc of an ION-CCI rat receiving a vehicle. S10 Fig. High magnification photomicrograph of pERK-IR neurons in the Vc of an ION-CCI rat receiving CGRP. S11 Fig. High magnification photomicrograph of pERK-IR neurons in the Vc of an ION-CCI rat receiving control IgG. S12 Fig. High magnification photomicrograph of pERK-IR neurons in the Vc of an ION-CCI rat receiving an anti-CGRP antibody. S13 Fig. Immunofluorescent image of pERK in the Vc. S14 Fig. Immunofluorescent image of neuronal nuclei (NeuN) in the Vc. S15 Fig. Merged image of pERK and NeuN in the Vc. S16 Fig. Immunofluorescent image of pERK in the Vc. S17 Fig. Immunofluorescent image of dopamineD2 receptor (D2 receptor) in the Vc. S18 Fig. Merged image of pERK and D2 receptor in the Vc. S19 Fig. Immunofluorescent image of receptor activity modifying protein 1 (RAMP1) in the Vc. S20 Fig. Immunofluorescent image of D2 receptor in the Vc. S21 Fig. Merged image of RAMP1 and D2 receptor in the Vc. S22 Fig. Photomicrograph of CGRP-IR neurons in the trige [file pone.0323810.s001.zip › supplementary/Fig S12.TIF]

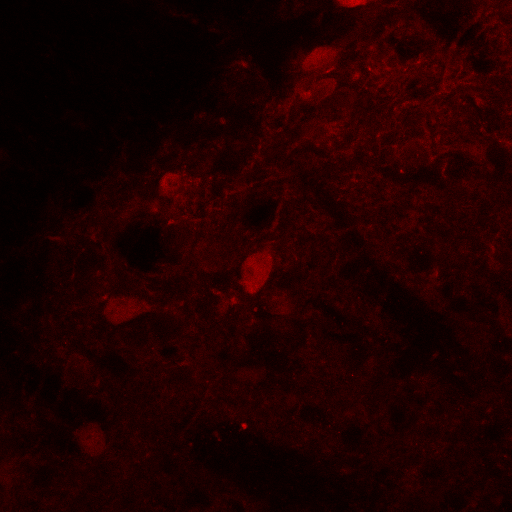

Supplement: S1 Data — S1 Fig. Photomicrograph of phosphorylated extracellular signal-regulated kinase (pERK)-immunoreactive (-IR) neurons in the trigeminal spinal subnucleus caudalis (Vc) of a sham rat. S2 Fig. Photomicrograph of pERK-IR neurons in the Vc of a chronic constriction injury of the infraorbital nerve (ION-CCI) rat. S3 Fig. Photomicrograph of pERK-IR neurons in the Vc of an ION-CCI rat receiving a vehicle. S4 Fig. Photomicrograph of pERK-IR neurons in the Vc of an ION-CCI rat receiving calcitonin gene-related peptide (CGRP). S5 Fig. Photomicrograph of pERK-IR neurons in the Vc of an ION-CCI rat receiving control immunogloublin G (IgG). S6 Fig. Photomicrograph of pERK-IR neurons in the Vc of an ION-CCI rat receiving an anti-CGRP antibody. S7 Fig. High magnification photomicrograph of pERK-IR neurons in the Vc of a sham rat. S8 Fig. High magnification photomicrograph of pERK-IR neurons in the Vc of an ION-CCI rat. S9 Fig. High magnification photomicrograph of pERK-IR neurons in the Vc of an ION-CCI rat receiving a vehicle. S10 Fig. High magnification photomicrograph of pERK-IR neurons in the Vc of an ION-CCI rat receiving CGRP. S11 Fig. High magnification photomicrograph of pERK-IR neurons in the Vc of an ION-CCI rat receiving control IgG. S12 Fig. High magnification photomicrograph of pERK-IR neurons in the Vc of an ION-CCI rat receiving an anti-CGRP antibody. S13 Fig. Immunofluorescent image of pERK in the Vc. S14 Fig. Immunofluorescent image of neuronal nuclei (NeuN) in the Vc. S15 Fig. Merged image of pERK and NeuN in the Vc. S16 Fig. Immunofluorescent image of pERK in the Vc. S17 Fig. Immunofluorescent image of dopamineD2 receptor (D2 receptor) in the Vc. S18 Fig. Merged image of pERK and D2 receptor in the Vc. S19 Fig. Immunofluorescent image of receptor activity modifying protein 1 (RAMP1) in the Vc. S20 Fig. Immunofluorescent image of D2 receptor in the Vc. S21 Fig. Merged image of RAMP1 and D2 receptor in the Vc. S22 Fig. Photomicrograph of CGRP-IR neurons in the trige [file pone.0323810.s001.zip › supplementary/Fig S13.tif]

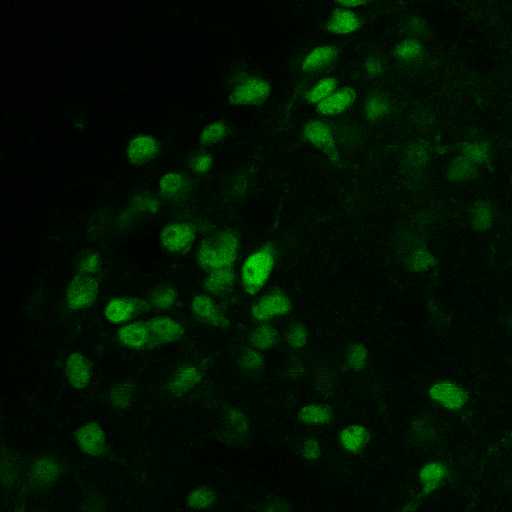

Supplement: S1 Data — S1 Fig. Photomicrograph of phosphorylated extracellular signal-regulated kinase (pERK)-immunoreactive (-IR) neurons in the trigeminal spinal subnucleus caudalis (Vc) of a sham rat. S2 Fig. Photomicrograph of pERK-IR neurons in the Vc of a chronic constriction injury of the infraorbital nerve (ION-CCI) rat. S3 Fig. Photomicrograph of pERK-IR neurons in the Vc of an ION-CCI rat receiving a vehicle. S4 Fig. Photomicrograph of pERK-IR neurons in the Vc of an ION-CCI rat receiving calcitonin gene-related peptide (CGRP). S5 Fig. Photomicrograph of pERK-IR neurons in the Vc of an ION-CCI rat receiving control immunogloublin G (IgG). S6 Fig. Photomicrograph of pERK-IR neurons in the Vc of an ION-CCI rat receiving an anti-CGRP antibody. S7 Fig. High magnification photomicrograph of pERK-IR neurons in the Vc of a sham rat. S8 Fig. High magnification photomicrograph of pERK-IR neurons in the Vc of an ION-CCI rat. S9 Fig. High magnification photomicrograph of pERK-IR neurons in the Vc of an ION-CCI rat receiving a vehicle. S10 Fig. High magnification photomicrograph of pERK-IR neurons in the Vc of an ION-CCI rat receiving CGRP. S11 Fig. High magnification photomicrograph of pERK-IR neurons in the Vc of an ION-CCI rat receiving control IgG. S12 Fig. High magnification photomicrograph of pERK-IR neurons in the Vc of an ION-CCI rat receiving an anti-CGRP antibody. S13 Fig. Immunofluorescent image of pERK in the Vc. S14 Fig. Immunofluorescent image of neuronal nuclei (NeuN) in the Vc. S15 Fig. Merged image of pERK and NeuN in the Vc. S16 Fig. Immunofluorescent image of pERK in the Vc. S17 Fig. Immunofluorescent image of dopamineD2 receptor (D2 receptor) in the Vc. S18 Fig. Merged image of pERK and D2 receptor in the Vc. S19 Fig. Immunofluorescent image of receptor activity modifying protein 1 (RAMP1) in the Vc. S20 Fig. Immunofluorescent image of D2 receptor in the Vc. S21 Fig. Merged image of RAMP1 and D2 receptor in the Vc. S22 Fig. Photomicrograph of CGRP-IR neurons in the trige [file pone.0323810.s001.zip › supplementary/Fig S14.tif]

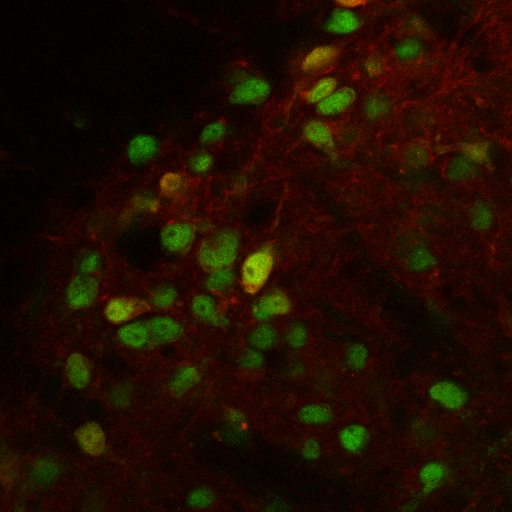

Supplement: S1 Data — S1 Fig. Photomicrograph of phosphorylated extracellular signal-regulated kinase (pERK)-immunoreactive (-IR) neurons in the trigeminal spinal subnucleus caudalis (Vc) of a sham rat. S2 Fig. Photomicrograph of pERK-IR neurons in the Vc of a chronic constriction injury of the infraorbital nerve (ION-CCI) rat. S3 Fig. Photomicrograph of pERK-IR neurons in the Vc of an ION-CCI rat receiving a vehicle. S4 Fig. Photomicrograph of pERK-IR neurons in the Vc of an ION-CCI rat receiving calcitonin gene-related peptide (CGRP). S5 Fig. Photomicrograph of pERK-IR neurons in the Vc of an ION-CCI rat receiving control immunogloublin G (IgG). S6 Fig. Photomicrograph of pERK-IR neurons in the Vc of an ION-CCI rat receiving an anti-CGRP antibody. S7 Fig. High magnification photomicrograph of pERK-IR neurons in the Vc of a sham rat. S8 Fig. High magnification photomicrograph of pERK-IR neurons in the Vc of an ION-CCI rat. S9 Fig. High magnification photomicrograph of pERK-IR neurons in the Vc of an ION-CCI rat receiving a vehicle. S10 Fig. High magnification photomicrograph of pERK-IR neurons in the Vc of an ION-CCI rat receiving CGRP. S11 Fig. High magnification photomicrograph of pERK-IR neurons in the Vc of an ION-CCI rat receiving control IgG. S12 Fig. High magnification photomicrograph of pERK-IR neurons in the Vc of an ION-CCI rat receiving an anti-CGRP antibody. S13 Fig. Immunofluorescent image of pERK in the Vc. S14 Fig. Immunofluorescent image of neuronal nuclei (NeuN) in the Vc. S15 Fig. Merged image of pERK and NeuN in the Vc. S16 Fig. Immunofluorescent image of pERK in the Vc. S17 Fig. Immunofluorescent image of dopamineD2 receptor (D2 receptor) in the Vc. S18 Fig. Merged image of pERK and D2 receptor in the Vc. S19 Fig. Immunofluorescent image of receptor activity modifying protein 1 (RAMP1) in the Vc. S20 Fig. Immunofluorescent image of D2 receptor in the Vc. S21 Fig. Merged image of RAMP1 and D2 receptor in the Vc. S22 Fig. Photomicrograph of CGRP-IR neurons in the trige [file pone.0323810.s001.zip › supplementary/Fig S15.tif]

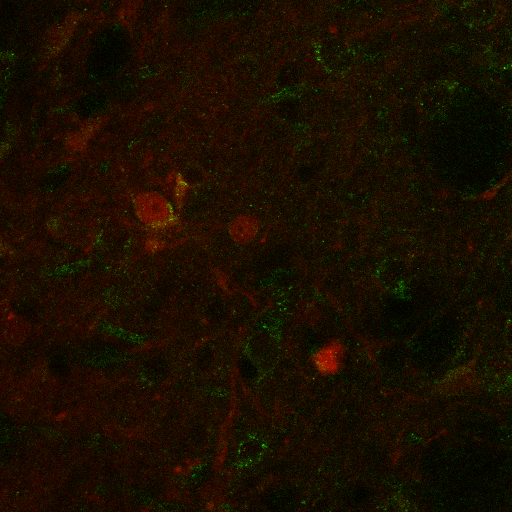

Supplement: S1 Data — S1 Fig. Photomicrograph of phosphorylated extracellular signal-regulated kinase (pERK)-immunoreactive (-IR) neurons in the trigeminal spinal subnucleus caudalis (Vc) of a sham rat. S2 Fig. Photomicrograph of pERK-IR neurons in the Vc of a chronic constriction injury of the infraorbital nerve (ION-CCI) rat. S3 Fig. Photomicrograph of pERK-IR neurons in the Vc of an ION-CCI rat receiving a vehicle. S4 Fig. Photomicrograph of pERK-IR neurons in the Vc of an ION-CCI rat receiving calcitonin gene-related peptide (CGRP). S5 Fig. Photomicrograph of pERK-IR neurons in the Vc of an ION-CCI rat receiving control immunogloublin G (IgG). S6 Fig. Photomicrograph of pERK-IR neurons in the Vc of an ION-CCI rat receiving an anti-CGRP antibody. S7 Fig. High magnification photomicrograph of pERK-IR neurons in the Vc of a sham rat. S8 Fig. High magnification photomicrograph of pERK-IR neurons in the Vc of an ION-CCI rat. S9 Fig. High magnification photomicrograph of pERK-IR neurons in the Vc of an ION-CCI rat receiving a vehicle. S10 Fig. High magnification photomicrograph of pERK-IR neurons in the Vc of an ION-CCI rat receiving CGRP. S11 Fig. High magnification photomicrograph of pERK-IR neurons in the Vc of an ION-CCI rat receiving control IgG. S12 Fig. High magnification photomicrograph of pERK-IR neurons in the Vc of an ION-CCI rat receiving an anti-CGRP antibody. S13 Fig. Immunofluorescent image of pERK in the Vc. S14 Fig. Immunofluorescent image of neuronal nuclei (NeuN) in the Vc. S15 Fig. Merged image of pERK and NeuN in the Vc. S16 Fig. Immunofluorescent image of pERK in the Vc. S17 Fig. Immunofluorescent image of dopamineD2 receptor (D2 receptor) in the Vc. S18 Fig. Merged image of pERK and D2 receptor in the Vc. S19 Fig. Immunofluorescent image of receptor activity modifying protein 1 (RAMP1) in the Vc. S20 Fig. Immunofluorescent image of D2 receptor in the Vc. S21 Fig. Merged image of RAMP1 and D2 receptor in the Vc. S22 Fig. Photomicrograph of CGRP-IR neurons in the trige [file pone.0323810.s001.zip › supplementary/Fig S16.tif]

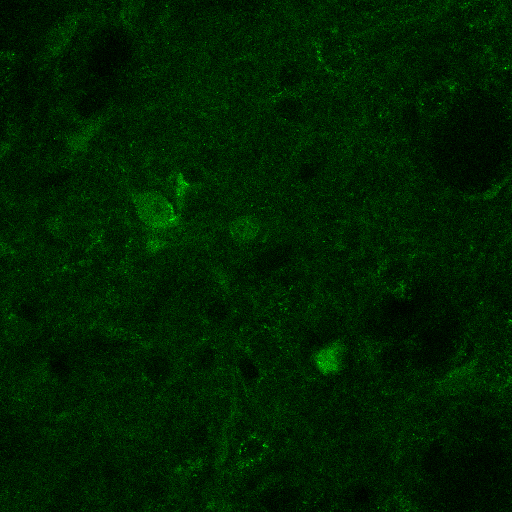

Supplement: S1 Data — S1 Fig. Photomicrograph of phosphorylated extracellular signal-regulated kinase (pERK)-immunoreactive (-IR) neurons in the trigeminal spinal subnucleus caudalis (Vc) of a sham rat. S2 Fig. Photomicrograph of pERK-IR neurons in the Vc of a chronic constriction injury of the infraorbital nerve (ION-CCI) rat. S3 Fig. Photomicrograph of pERK-IR neurons in the Vc of an ION-CCI rat receiving a vehicle. S4 Fig. Photomicrograph of pERK-IR neurons in the Vc of an ION-CCI rat receiving calcitonin gene-related peptide (CGRP). S5 Fig. Photomicrograph of pERK-IR neurons in the Vc of an ION-CCI rat receiving control immunogloublin G (IgG). S6 Fig. Photomicrograph of pERK-IR neurons in the Vc of an ION-CCI rat receiving an anti-CGRP antibody. S7 Fig. High magnification photomicrograph of pERK-IR neurons in the Vc of a sham rat. S8 Fig. High magnification photomicrograph of pERK-IR neurons in the Vc of an ION-CCI rat. S9 Fig. High magnification photomicrograph of pERK-IR neurons in the Vc of an ION-CCI rat receiving a vehicle. S10 Fig. High magnification photomicrograph of pERK-IR neurons in the Vc of an ION-CCI rat receiving CGRP. S11 Fig. High magnification photomicrograph of pERK-IR neurons in the Vc of an ION-CCI rat receiving control IgG. S12 Fig. High magnification photomicrograph of pERK-IR neurons in the Vc of an ION-CCI rat receiving an anti-CGRP antibody. S13 Fig. Immunofluorescent image of pERK in the Vc. S14 Fig. Immunofluorescent image of neuronal nuclei (NeuN) in the Vc. S15 Fig. Merged image of pERK and NeuN in the Vc. S16 Fig. Immunofluorescent image of pERK in the Vc. S17 Fig. Immunofluorescent image of dopamineD2 receptor (D2 receptor) in the Vc. S18 Fig. Merged image of pERK and D2 receptor in the Vc. S19 Fig. Immunofluorescent image of receptor activity modifying protein 1 (RAMP1) in the Vc. S20 Fig. Immunofluorescent image of D2 receptor in the Vc. S21 Fig. Merged image of RAMP1 and D2 receptor in the Vc. S22 Fig. Photomicrograph of CGRP-IR neurons in the trige [file pone.0323810.s001.zip › supplementary/Fig S17.tif]

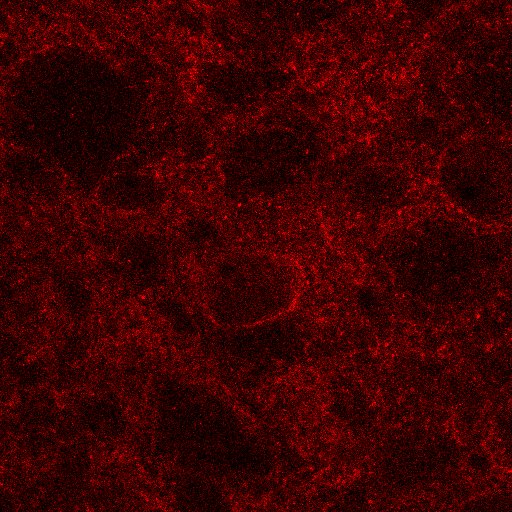

Supplement: S1 Data — S1 Fig. Photomicrograph of phosphorylated extracellular signal-regulated kinase (pERK)-immunoreactive (-IR) neurons in the trigeminal spinal subnucleus caudalis (Vc) of a sham rat. S2 Fig. Photomicrograph of pERK-IR neurons in the Vc of a chronic constriction injury of the infraorbital nerve (ION-CCI) rat. S3 Fig. Photomicrograph of pERK-IR neurons in the Vc of an ION-CCI rat receiving a vehicle. S4 Fig. Photomicrograph of pERK-IR neurons in the Vc of an ION-CCI rat receiving calcitonin gene-related peptide (CGRP). S5 Fig. Photomicrograph of pERK-IR neurons in the Vc of an ION-CCI rat receiving control immunogloublin G (IgG). S6 Fig. Photomicrograph of pERK-IR neurons in the Vc of an ION-CCI rat receiving an anti-CGRP antibody. S7 Fig. High magnification photomicrograph of pERK-IR neurons in the Vc of a sham rat. S8 Fig. High magnification photomicrograph of pERK-IR neurons in the Vc of an ION-CCI rat. S9 Fig. High magnification photomicrograph of pERK-IR neurons in the Vc of an ION-CCI rat receiving a vehicle. S10 Fig. High magnification photomicrograph of pERK-IR neurons in the Vc of an ION-CCI rat receiving CGRP. S11 Fig. High magnification photomicrograph of pERK-IR neurons in the Vc of an ION-CCI rat receiving control IgG. S12 Fig. High magnification photomicrograph of pERK-IR neurons in the Vc of an ION-CCI rat receiving an anti-CGRP antibody. S13 Fig. Immunofluorescent image of pERK in the Vc. S14 Fig. Immunofluorescent image of neuronal nuclei (NeuN) in the Vc. S15 Fig. Merged image of pERK and NeuN in the Vc. S16 Fig. Immunofluorescent image of pERK in the Vc. S17 Fig. Immunofluorescent image of dopamineD2 receptor (D2 receptor) in the Vc. S18 Fig. Merged image of pERK and D2 receptor in the Vc. S19 Fig. Immunofluorescent image of receptor activity modifying protein 1 (RAMP1) in the Vc. S20 Fig. Immunofluorescent image of D2 receptor in the Vc. S21 Fig. Merged image of RAMP1 and D2 receptor in the Vc. S22 Fig. Photomicrograph of CGRP-IR neurons in the trige [file pone.0323810.s001.zip › supplementary/Fig S19.tif]

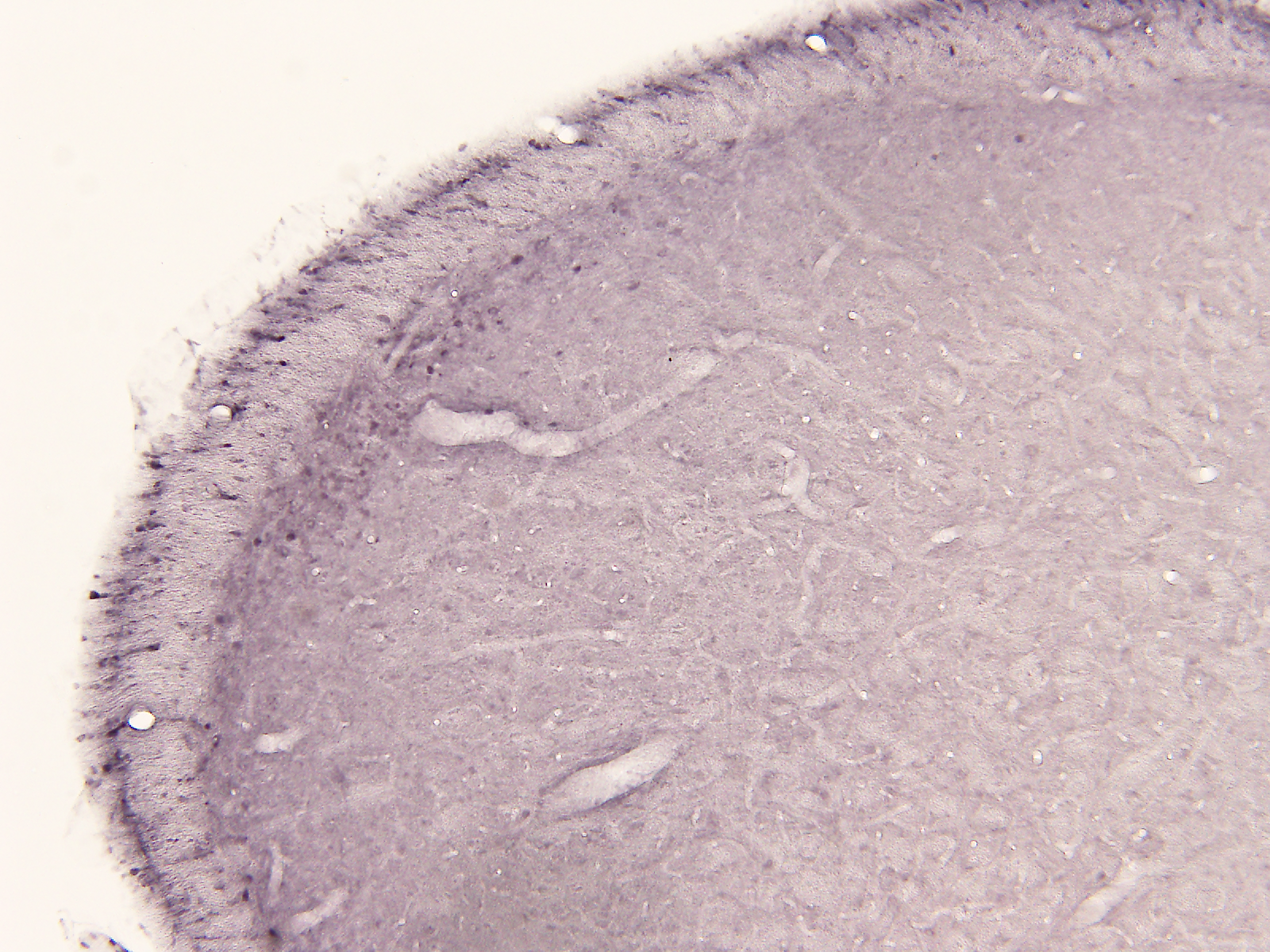

Supplement: S1 Data — S1 Fig. Photomicrograph of phosphorylated extracellular signal-regulated kinase (pERK)-immunoreactive (-IR) neurons in the trigeminal spinal subnucleus caudalis (Vc) of a sham rat. S2 Fig. Photomicrograph of pERK-IR neurons in the Vc of a chronic constriction injury of the infraorbital nerve (ION-CCI) rat. S3 Fig. Photomicrograph of pERK-IR neurons in the Vc of an ION-CCI rat receiving a vehicle. S4 Fig. Photomicrograph of pERK-IR neurons in the Vc of an ION-CCI rat receiving calcitonin gene-related peptide (CGRP). S5 Fig. Photomicrograph of pERK-IR neurons in the Vc of an ION-CCI rat receiving control immunogloublin G (IgG). S6 Fig. Photomicrograph of pERK-IR neurons in the Vc of an ION-CCI rat receiving an anti-CGRP antibody. S7 Fig. High magnification photomicrograph of pERK-IR neurons in the Vc of a sham rat. S8 Fig. High magnification photomicrograph of pERK-IR neurons in the Vc of an ION-CCI rat. S9 Fig. High magnification photomicrograph of pERK-IR neurons in the Vc of an ION-CCI rat receiving a vehicle. S10 Fig. High magnification photomicrograph of pERK-IR neurons in the Vc of an ION-CCI rat receiving CGRP. S11 Fig. High magnification photomicrograph of pERK-IR neurons in the Vc of an ION-CCI rat receiving control IgG. S12 Fig. High magnification photomicrograph of pERK-IR neurons in the Vc of an ION-CCI rat receiving an anti-CGRP antibody. S13 Fig. Immunofluorescent image of pERK in the Vc. S14 Fig. Immunofluorescent image of neuronal nuclei (NeuN) in the Vc. S15 Fig. Merged image of pERK and NeuN in the Vc. S16 Fig. Immunofluorescent image of pERK in the Vc. S17 Fig. Immunofluorescent image of dopamineD2 receptor (D2 receptor) in the Vc. S18 Fig. Merged image of pERK and D2 receptor in the Vc. S19 Fig. Immunofluorescent image of receptor activity modifying protein 1 (RAMP1) in the Vc. S20 Fig. Immunofluorescent image of D2 receptor in the Vc. S21 Fig. Merged image of RAMP1 and D2 receptor in the Vc. S22 Fig. Photomicrograph of CGRP-IR neurons in the trige [file pone.0323810.s001.zip › supplementary/Fig S2.TIF]

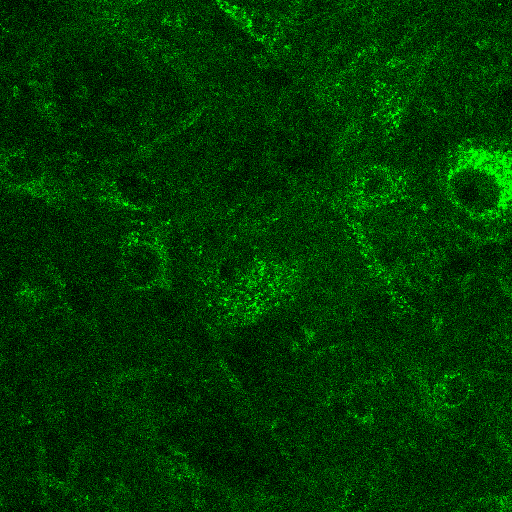

Supplement: S1 Data — S1 Fig. Photomicrograph of phosphorylated extracellular signal-regulated kinase (pERK)-immunoreactive (-IR) neurons in the trigeminal spinal subnucleus caudalis (Vc) of a sham rat. S2 Fig. Photomicrograph of pERK-IR neurons in the Vc of a chronic constriction injury of the infraorbital nerve (ION-CCI) rat. S3 Fig. Photomicrograph of pERK-IR neurons in the Vc of an ION-CCI rat receiving a vehicle. S4 Fig. Photomicrograph of pERK-IR neurons in the Vc of an ION-CCI rat receiving calcitonin gene-related peptide (CGRP). S5 Fig. Photomicrograph of pERK-IR neurons in the Vc of an ION-CCI rat receiving control immunogloublin G (IgG). S6 Fig. Photomicrograph of pERK-IR neurons in the Vc of an ION-CCI rat receiving an anti-CGRP antibody. S7 Fig. High magnification photomicrograph of pERK-IR neurons in the Vc of a sham rat. S8 Fig. High magnification photomicrograph of pERK-IR neurons in the Vc of an ION-CCI rat. S9 Fig. High magnification photomicrograph of pERK-IR neurons in the Vc of an ION-CCI rat receiving a vehicle. S10 Fig. High magnification photomicrograph of pERK-IR neurons in the Vc of an ION-CCI rat receiving CGRP. S11 Fig. High magnification photomicrograph of pERK-IR neurons in the Vc of an ION-CCI rat receiving control IgG. S12 Fig. High magnification photomicrograph of pERK-IR neurons in the Vc of an ION-CCI rat receiving an anti-CGRP antibody. S13 Fig. Immunofluorescent image of pERK in the Vc. S14 Fig. Immunofluorescent image of neuronal nuclei (NeuN) in the Vc. S15 Fig. Merged image of pERK and NeuN in the Vc. S16 Fig. Immunofluorescent image of pERK in the Vc. S17 Fig. Immunofluorescent image of dopamineD2 receptor (D2 receptor) in the Vc. S18 Fig. Merged image of pERK and D2 receptor in the Vc. S19 Fig. Immunofluorescent image of receptor activity modifying protein 1 (RAMP1) in the Vc. S20 Fig. Immunofluorescent image of D2 receptor in the Vc. S21 Fig. Merged image of RAMP1 and D2 receptor in the Vc. S22 Fig. Photomicrograph of CGRP-IR neurons in the trige [file pone.0323810.s001.zip › supplementary/Fig S20.tif]

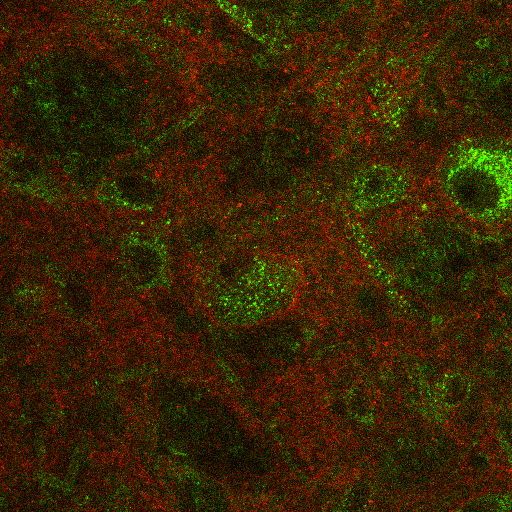

Supplement: S1 Data — S1 Fig. Photomicrograph of phosphorylated extracellular signal-regulated kinase (pERK)-immunoreactive (-IR) neurons in the trigeminal spinal subnucleus caudalis (Vc) of a sham rat. S2 Fig. Photomicrograph of pERK-IR neurons in the Vc of a chronic constriction injury of the infraorbital nerve (ION-CCI) rat. S3 Fig. Photomicrograph of pERK-IR neurons in the Vc of an ION-CCI rat receiving a vehicle. S4 Fig. Photomicrograph of pERK-IR neurons in the Vc of an ION-CCI rat receiving calcitonin gene-related peptide (CGRP). S5 Fig. Photomicrograph of pERK-IR neurons in the Vc of an ION-CCI rat receiving control immunogloublin G (IgG). S6 Fig. Photomicrograph of pERK-IR neurons in the Vc of an ION-CCI rat receiving an anti-CGRP antibody. S7 Fig. High magnification photomicrograph of pERK-IR neurons in the Vc of a sham rat. S8 Fig. High magnification photomicrograph of pERK-IR neurons in the Vc of an ION-CCI rat. S9 Fig. High magnification photomicrograph of pERK-IR neurons in the Vc of an ION-CCI rat receiving a vehicle. S10 Fig. High magnification photomicrograph of pERK-IR neurons in the Vc of an ION-CCI rat receiving CGRP. S11 Fig. High magnification photomicrograph of pERK-IR neurons in the Vc of an ION-CCI rat receiving control IgG. S12 Fig. High magnification photomicrograph of pERK-IR neurons in the Vc of an ION-CCI rat receiving an anti-CGRP antibody. S13 Fig. Immunofluorescent image of pERK in the Vc. S14 Fig. Immunofluorescent image of neuronal nuclei (NeuN) in the Vc. S15 Fig. Merged image of pERK and NeuN in the Vc. S16 Fig. Immunofluorescent image of pERK in the Vc. S17 Fig. Immunofluorescent image of dopamineD2 receptor (D2 receptor) in the Vc. S18 Fig. Merged image of pERK and D2 receptor in the Vc. S19 Fig. Immunofluorescent image of receptor activity modifying protein 1 (RAMP1) in the Vc. S20 Fig. Immunofluorescent image of D2 receptor in the Vc. S21 Fig. Merged image of RAMP1 and D2 receptor in the Vc. S22 Fig. Photomicrograph of CGRP-IR neurons in the trige [file pone.0323810.s001.zip › supplementary/Fig S21.tif]

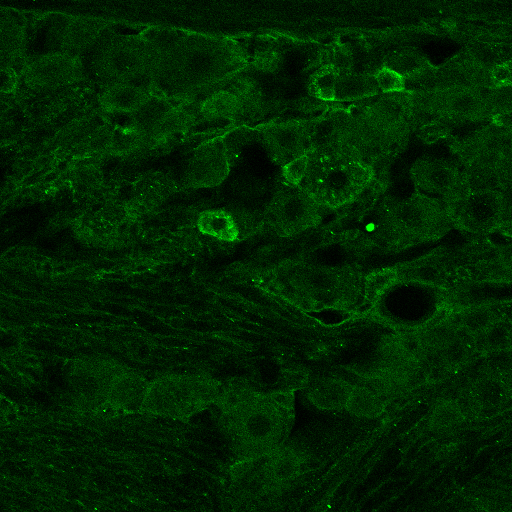

Supplement: S1 Data — S1 Fig. Photomicrograph of phosphorylated extracellular signal-regulated kinase (pERK)-immunoreactive (-IR) neurons in the trigeminal spinal subnucleus caudalis (Vc) of a sham rat. S2 Fig. Photomicrograph of pERK-IR neurons in the Vc of a chronic constriction injury of the infraorbital nerve (ION-CCI) rat. S3 Fig. Photomicrograph of pERK-IR neurons in the Vc of an ION-CCI rat receiving a vehicle. S4 Fig. Photomicrograph of pERK-IR neurons in the Vc of an ION-CCI rat receiving calcitonin gene-related peptide (CGRP). S5 Fig. Photomicrograph of pERK-IR neurons in the Vc of an ION-CCI rat receiving control immunogloublin G (IgG). S6 Fig. Photomicrograph of pERK-IR neurons in the Vc of an ION-CCI rat receiving an anti-CGRP antibody. S7 Fig. High magnification photomicrograph of pERK-IR neurons in the Vc of a sham rat. S8 Fig. High magnification photomicrograph of pERK-IR neurons in the Vc of an ION-CCI rat. S9 Fig. High magnification photomicrograph of pERK-IR neurons in the Vc of an ION-CCI rat receiving a vehicle. S10 Fig. High magnification photomicrograph of pERK-IR neurons in the Vc of an ION-CCI rat receiving CGRP. S11 Fig. High magnification photomicrograph of pERK-IR neurons in the Vc of an ION-CCI rat receiving control IgG. S12 Fig. High magnification photomicrograph of pERK-IR neurons in the Vc of an ION-CCI rat receiving an anti-CGRP antibody. S13 Fig. Immunofluorescent image of pERK in the Vc. S14 Fig. Immunofluorescent image of neuronal nuclei (NeuN) in the Vc. S15 Fig. Merged image of pERK and NeuN in the Vc. S16 Fig. Immunofluorescent image of pERK in the Vc. S17 Fig. Immunofluorescent image of dopamineD2 receptor (D2 receptor) in the Vc. S18 Fig. Merged image of pERK and D2 receptor in the Vc. S19 Fig. Immunofluorescent image of receptor activity modifying protein 1 (RAMP1) in the Vc. S20 Fig. Immunofluorescent image of D2 receptor in the Vc. S21 Fig. Merged image of RAMP1 and D2 receptor in the Vc. S22 Fig. Photomicrograph of CGRP-IR neurons in the trige [file pone.0323810.s001.zip › supplementary/Fig S22.tif]

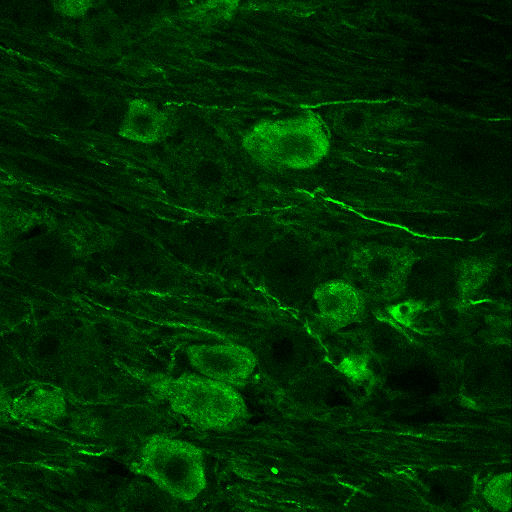

Supplement: S1 Data — S1 Fig. Photomicrograph of phosphorylated extracellular signal-regulated kinase (pERK)-immunoreactive (-IR) neurons in the trigeminal spinal subnucleus caudalis (Vc) of a sham rat. S2 Fig. Photomicrograph of pERK-IR neurons in the Vc of a chronic constriction injury of the infraorbital nerve (ION-CCI) rat. S3 Fig. Photomicrograph of pERK-IR neurons in the Vc of an ION-CCI rat receiving a vehicle. S4 Fig. Photomicrograph of pERK-IR neurons in the Vc of an ION-CCI rat receiving calcitonin gene-related peptide (CGRP). S5 Fig. Photomicrograph of pERK-IR neurons in the Vc of an ION-CCI rat receiving control immunogloublin G (IgG). S6 Fig. Photomicrograph of pERK-IR neurons in the Vc of an ION-CCI rat receiving an anti-CGRP antibody. S7 Fig. High magnification photomicrograph of pERK-IR neurons in the Vc of a sham rat. S8 Fig. High magnification photomicrograph of pERK-IR neurons in the Vc of an ION-CCI rat. S9 Fig. High magnification photomicrograph of pERK-IR neurons in the Vc of an ION-CCI rat receiving a vehicle. S10 Fig. High magnification photomicrograph of pERK-IR neurons in the Vc of an ION-CCI rat receiving CGRP. S11 Fig. High magnification photomicrograph of pERK-IR neurons in the Vc of an ION-CCI rat receiving control IgG. S12 Fig. High magnification photomicrograph of pERK-IR neurons in the Vc of an ION-CCI rat receiving an anti-CGRP antibody. S13 Fig. Immunofluorescent image of pERK in the Vc. S14 Fig. Immunofluorescent image of neuronal nuclei (NeuN) in the Vc. S15 Fig. Merged image of pERK and NeuN in the Vc. S16 Fig. Immunofluorescent image of pERK in the Vc. S17 Fig. Immunofluorescent image of dopamineD2 receptor (D2 receptor) in the Vc. S18 Fig. Merged image of pERK and D2 receptor in the Vc. S19 Fig. Immunofluorescent image of receptor activity modifying protein 1 (RAMP1) in the Vc. S20 Fig. Immunofluorescent image of D2 receptor in the Vc. S21 Fig. Merged image of RAMP1 and D2 receptor in the Vc. S22 Fig. Photomicrograph of CGRP-IR neurons in the trige [file pone.0323810.s001.zip › supplementary/Fig S23.tif]

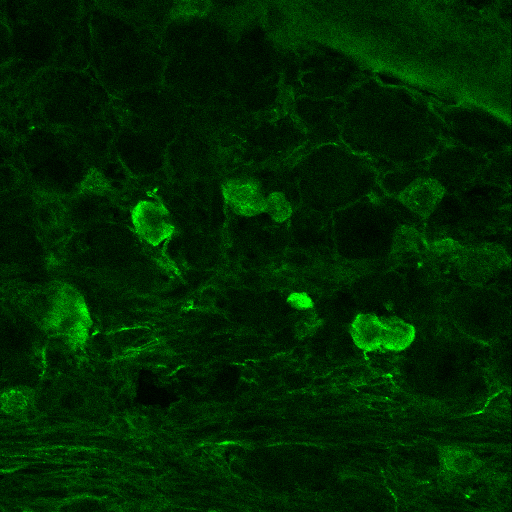

Supplement: S1 Data — S1 Fig. Photomicrograph of phosphorylated extracellular signal-regulated kinase (pERK)-immunoreactive (-IR) neurons in the trigeminal spinal subnucleus caudalis (Vc) of a sham rat. S2 Fig. Photomicrograph of pERK-IR neurons in the Vc of a chronic constriction injury of the infraorbital nerve (ION-CCI) rat. S3 Fig. Photomicrograph of pERK-IR neurons in the Vc of an ION-CCI rat receiving a vehicle. S4 Fig. Photomicrograph of pERK-IR neurons in the Vc of an ION-CCI rat receiving calcitonin gene-related peptide (CGRP). S5 Fig. Photomicrograph of pERK-IR neurons in the Vc of an ION-CCI rat receiving control immunogloublin G (IgG). S6 Fig. Photomicrograph of pERK-IR neurons in the Vc of an ION-CCI rat receiving an anti-CGRP antibody. S7 Fig. High magnification photomicrograph of pERK-IR neurons in the Vc of a sham rat. S8 Fig. High magnification photomicrograph of pERK-IR neurons in the Vc of an ION-CCI rat. S9 Fig. High magnification photomicrograph of pERK-IR neurons in the Vc of an ION-CCI rat receiving a vehicle. S10 Fig. High magnification photomicrograph of pERK-IR neurons in the Vc of an ION-CCI rat receiving CGRP. S11 Fig. High magnification photomicrograph of pERK-IR neurons in the Vc of an ION-CCI rat receiving control IgG. S12 Fig. High magnification photomicrograph of pERK-IR neurons in the Vc of an ION-CCI rat receiving an anti-CGRP antibody. S13 Fig. Immunofluorescent image of pERK in the Vc. S14 Fig. Immunofluorescent image of neuronal nuclei (NeuN) in the Vc. S15 Fig. Merged image of pERK and NeuN in the Vc. S16 Fig. Immunofluorescent image of pERK in the Vc. S17 Fig. Immunofluorescent image of dopamineD2 receptor (D2 receptor) in the Vc. S18 Fig. Merged image of pERK and D2 receptor in the Vc. S19 Fig. Immunofluorescent image of receptor activity modifying protein 1 (RAMP1) in the Vc. S20 Fig. Immunofluorescent image of D2 receptor in the Vc. S21 Fig. Merged image of RAMP1 and D2 receptor in the Vc. S22 Fig. Photomicrograph of CGRP-IR neurons in the trige [file pone.0323810.s001.zip › supplementary/Fig S24.tif]

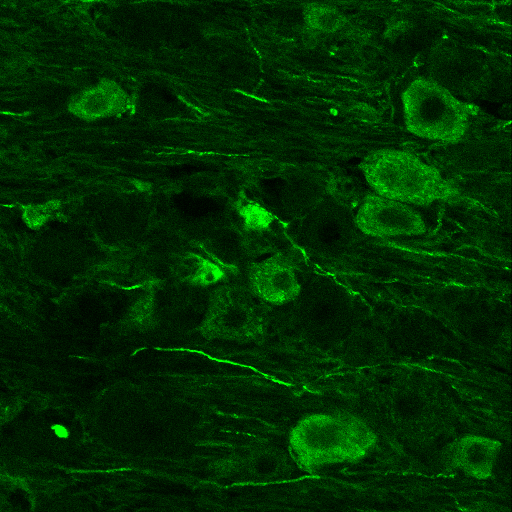

Supplement: S1 Data — S1 Fig. Photomicrograph of phosphorylated extracellular signal-regulated kinase (pERK)-immunoreactive (-IR) neurons in the trigeminal spinal subnucleus caudalis (Vc) of a sham rat. S2 Fig. Photomicrograph of pERK-IR neurons in the Vc of a chronic constriction injury of the infraorbital nerve (ION-CCI) rat. S3 Fig. Photomicrograph of pERK-IR neurons in the Vc of an ION-CCI rat receiving a vehicle. S4 Fig. Photomicrograph of pERK-IR neurons in the Vc of an ION-CCI rat receiving calcitonin gene-related peptide (CGRP). S5 Fig. Photomicrograph of pERK-IR neurons in the Vc of an ION-CCI rat receiving control immunogloublin G (IgG). S6 Fig. Photomicrograph of pERK-IR neurons in the Vc of an ION-CCI rat receiving an anti-CGRP antibody. S7 Fig. High magnification photomicrograph of pERK-IR neurons in the Vc of a sham rat. S8 Fig. High magnification photomicrograph of pERK-IR neurons in the Vc of an ION-CCI rat. S9 Fig. High magnification photomicrograph of pERK-IR neurons in the Vc of an ION-CCI rat receiving a vehicle. S10 Fig. High magnification photomicrograph of pERK-IR neurons in the Vc of an ION-CCI rat receiving CGRP. S11 Fig. High magnification photomicrograph of pERK-IR neurons in the Vc of an ION-CCI rat receiving control IgG. S12 Fig. High magnification photomicrograph of pERK-IR neurons in the Vc of an ION-CCI rat receiving an anti-CGRP antibody. S13 Fig. Immunofluorescent image of pERK in the Vc. S14 Fig. Immunofluorescent image of neuronal nuclei (NeuN) in the Vc. S15 Fig. Merged image of pERK and NeuN in the Vc. S16 Fig. Immunofluorescent image of pERK in the Vc. S17 Fig. Immunofluorescent image of dopamineD2 receptor (D2 receptor) in the Vc. S18 Fig. Merged image of pERK and D2 receptor in the Vc. S19 Fig. Immunofluorescent image of receptor activity modifying protein 1 (RAMP1) in the Vc. S20 Fig. Immunofluorescent image of D2 receptor in the Vc. S21 Fig. Merged image of RAMP1 and D2 receptor in the Vc. S22 Fig. Photomicrograph of CGRP-IR neurons in the trige [file pone.0323810.s001.zip › supplementary/Fig S25.tif]

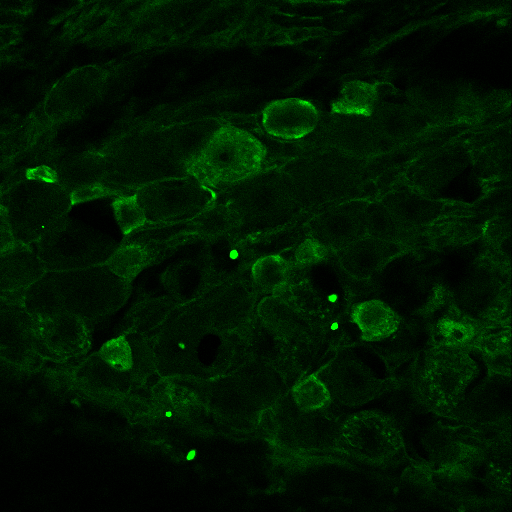

Supplement: S1 Data — S1 Fig. Photomicrograph of phosphorylated extracellular signal-regulated kinase (pERK)-immunoreactive (-IR) neurons in the trigeminal spinal subnucleus caudalis (Vc) of a sham rat. S2 Fig. Photomicrograph of pERK-IR neurons in the Vc of a chronic constriction injury of the infraorbital nerve (ION-CCI) rat. S3 Fig. Photomicrograph of pERK-IR neurons in the Vc of an ION-CCI rat receiving a vehicle. S4 Fig. Photomicrograph of pERK-IR neurons in the Vc of an ION-CCI rat receiving calcitonin gene-related peptide (CGRP). S5 Fig. Photomicrograph of pERK-IR neurons in the Vc of an ION-CCI rat receiving control immunogloublin G (IgG). S6 Fig. Photomicrograph of pERK-IR neurons in the Vc of an ION-CCI rat receiving an anti-CGRP antibody. S7 Fig. High magnification photomicrograph of pERK-IR neurons in the Vc of a sham rat. S8 Fig. High magnification photomicrograph of pERK-IR neurons in the Vc of an ION-CCI rat. S9 Fig. High magnification photomicrograph of pERK-IR neurons in the Vc of an ION-CCI rat receiving a vehicle. S10 Fig. High magnification photomicrograph of pERK-IR neurons in the Vc of an ION-CCI rat receiving CGRP. S11 Fig. High magnification photomicrograph of pERK-IR neurons in the Vc of an ION-CCI rat receiving control IgG. S12 Fig. High magnification photomicrograph of pERK-IR neurons in the Vc of an ION-CCI rat receiving an anti-CGRP antibody. S13 Fig. Immunofluorescent image of pERK in the Vc. S14 Fig. Immunofluorescent image of neuronal nuclei (NeuN) in the Vc. S15 Fig. Merged image of pERK and NeuN in the Vc. S16 Fig. Immunofluorescent image of pERK in the Vc. S17 Fig. Immunofluorescent image of dopamineD2 receptor (D2 receptor) in the Vc. S18 Fig. Merged image of pERK and D2 receptor in the Vc. S19 Fig. Immunofluorescent image of receptor activity modifying protein 1 (RAMP1) in the Vc. S20 Fig. Immunofluorescent image of D2 receptor in the Vc. S21 Fig. Merged image of RAMP1 and D2 receptor in the Vc. S22 Fig. Photomicrograph of CGRP-IR neurons in the trige [file pone.0323810.s001.zip › supplementary/Fig S26.tif]

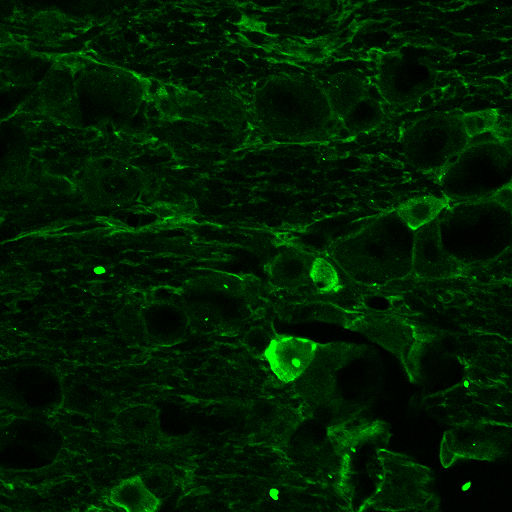

Supplement: S1 Data — S1 Fig. Photomicrograph of phosphorylated extracellular signal-regulated kinase (pERK)-immunoreactive (-IR) neurons in the trigeminal spinal subnucleus caudalis (Vc) of a sham rat. S2 Fig. Photomicrograph of pERK-IR neurons in the Vc of a chronic constriction injury of the infraorbital nerve (ION-CCI) rat. S3 Fig. Photomicrograph of pERK-IR neurons in the Vc of an ION-CCI rat receiving a vehicle. S4 Fig. Photomicrograph of pERK-IR neurons in the Vc of an ION-CCI rat receiving calcitonin gene-related peptide (CGRP). S5 Fig. Photomicrograph of pERK-IR neurons in the Vc of an ION-CCI rat receiving control immunogloublin G (IgG). S6 Fig. Photomicrograph of pERK-IR neurons in the Vc of an ION-CCI rat receiving an anti-CGRP antibody. S7 Fig. High magnification photomicrograph of pERK-IR neurons in the Vc of a sham rat. S8 Fig. High magnification photomicrograph of pERK-IR neurons in the Vc of an ION-CCI rat. S9 Fig. High magnification photomicrograph of pERK-IR neurons in the Vc of an ION-CCI rat receiving a vehicle. S10 Fig. High magnification photomicrograph of pERK-IR neurons in the Vc of an ION-CCI rat receiving CGRP. S11 Fig. High magnification photomicrograph of pERK-IR neurons in the Vc of an ION-CCI rat receiving control IgG. S12 Fig. High magnification photomicrograph of pERK-IR neurons in the Vc of an ION-CCI rat receiving an anti-CGRP antibody. S13 Fig. Immunofluorescent image of pERK in the Vc. S14 Fig. Immunofluorescent image of neuronal nuclei (NeuN) in the Vc. S15 Fig. Merged image of pERK and NeuN in the Vc. S16 Fig. Immunofluorescent image of pERK in the Vc. S17 Fig. Immunofluorescent image of dopamineD2 receptor (D2 receptor) in the Vc. S18 Fig. Merged image of pERK and D2 receptor in the Vc. S19 Fig. Immunofluorescent image of receptor activity modifying protein 1 (RAMP1) in the Vc. S20 Fig. Immunofluorescent image of D2 receptor in the Vc. S21 Fig. Merged image of RAMP1 and D2 receptor in the Vc. S22 Fig. Photomicrograph of CGRP-IR neurons in the trige [file pone.0323810.s001.zip › supplementary/Fig S27.tif]

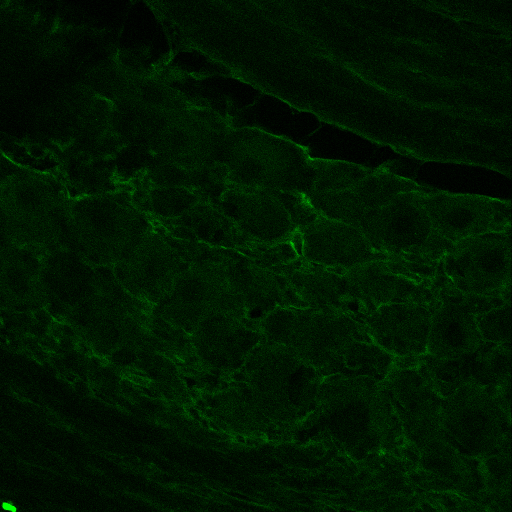

Supplement: S1 Data — S1 Fig. Photomicrograph of phosphorylated extracellular signal-regulated kinase (pERK)-immunoreactive (-IR) neurons in the trigeminal spinal subnucleus caudalis (Vc) of a sham rat. S2 Fig. Photomicrograph of pERK-IR neurons in the Vc of a chronic constriction injury of the infraorbital nerve (ION-CCI) rat. S3 Fig. Photomicrograph of pERK-IR neurons in the Vc of an ION-CCI rat receiving a vehicle. S4 Fig. Photomicrograph of pERK-IR neurons in the Vc of an ION-CCI rat receiving calcitonin gene-related peptide (CGRP). S5 Fig. Photomicrograph of pERK-IR neurons in the Vc of an ION-CCI rat receiving control immunogloublin G (IgG). S6 Fig. Photomicrograph of pERK-IR neurons in the Vc of an ION-CCI rat receiving an anti-CGRP antibody. S7 Fig. High magnification photomicrograph of pERK-IR neurons in the Vc of a sham rat. S8 Fig. High magnification photomicrograph of pERK-IR neurons in the Vc of an ION-CCI rat. S9 Fig. High magnification photomicrograph of pERK-IR neurons in the Vc of an ION-CCI rat receiving a vehicle. S10 Fig. High magnification photomicrograph of pERK-IR neurons in the Vc of an ION-CCI rat receiving CGRP. S11 Fig. High magnification photomicrograph of pERK-IR neurons in the Vc of an ION-CCI rat receiving control IgG. S12 Fig. High magnification photomicrograph of pERK-IR neurons in the Vc of an ION-CCI rat receiving an anti-CGRP antibody. S13 Fig. Immunofluorescent image of pERK in the Vc. S14 Fig. Immunofluorescent image of neuronal nuclei (NeuN) in the Vc. S15 Fig. Merged image of pERK and NeuN in the Vc. S16 Fig. Immunofluorescent image of pERK in the Vc. S17 Fig. Immunofluorescent image of dopamineD2 receptor (D2 receptor) in the Vc. S18 Fig. Merged image of pERK and D2 receptor in the Vc. S19 Fig. Immunofluorescent image of receptor activity modifying protein 1 (RAMP1) in the Vc. S20 Fig. Immunofluorescent image of D2 receptor in the Vc. S21 Fig. Merged image of RAMP1 and D2 receptor in the Vc. S22 Fig. Photomicrograph of CGRP-IR neurons in the trige [file pone.0323810.s001.zip › supplementary/Fig S28.tif]

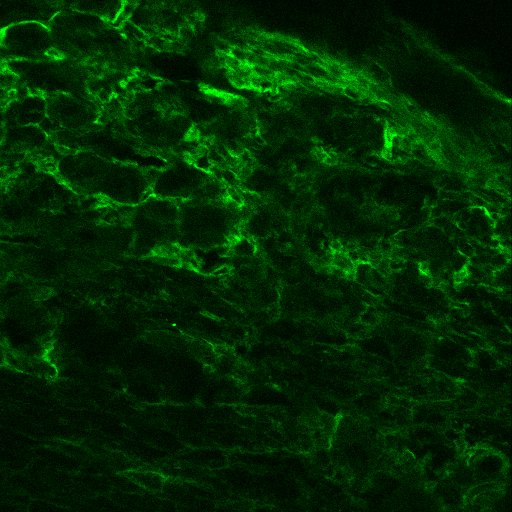

Supplement: S1 Data — S1 Fig. Photomicrograph of phosphorylated extracellular signal-regulated kinase (pERK)-immunoreactive (-IR) neurons in the trigeminal spinal subnucleus caudalis (Vc) of a sham rat. S2 Fig. Photomicrograph of pERK-IR neurons in the Vc of a chronic constriction injury of the infraorbital nerve (ION-CCI) rat. S3 Fig. Photomicrograph of pERK-IR neurons in the Vc of an ION-CCI rat receiving a vehicle. S4 Fig. Photomicrograph of pERK-IR neurons in the Vc of an ION-CCI rat receiving calcitonin gene-related peptide (CGRP). S5 Fig. Photomicrograph of pERK-IR neurons in the Vc of an ION-CCI rat receiving control immunogloublin G (IgG). S6 Fig. Photomicrograph of pERK-IR neurons in the Vc of an ION-CCI rat receiving an anti-CGRP antibody. S7 Fig. High magnification photomicrograph of pERK-IR neurons in the Vc of a sham rat. S8 Fig. High magnification photomicrograph of pERK-IR neurons in the Vc of an ION-CCI rat. S9 Fig. High magnification photomicrograph of pERK-IR neurons in the Vc of an ION-CCI rat receiving a vehicle. S10 Fig. High magnification photomicrograph of pERK-IR neurons in the Vc of an ION-CCI rat receiving CGRP. S11 Fig. High magnification photomicrograph of pERK-IR neurons in the Vc of an ION-CCI rat receiving control IgG. S12 Fig. High magnification photomicrograph of pERK-IR neurons in the Vc of an ION-CCI rat receiving an anti-CGRP antibody. S13 Fig. Immunofluorescent image of pERK in the Vc. S14 Fig. Immunofluorescent image of neuronal nuclei (NeuN) in the Vc. S15 Fig. Merged image of pERK and NeuN in the Vc. S16 Fig. Immunofluorescent image of pERK in the Vc. S17 Fig. Immunofluorescent image of dopamineD2 receptor (D2 receptor) in the Vc. S18 Fig. Merged image of pERK and D2 receptor in the Vc. S19 Fig. Immunofluorescent image of receptor activity modifying protein 1 (RAMP1) in the Vc. S20 Fig. Immunofluorescent image of D2 receptor in the Vc. S21 Fig. Merged image of RAMP1 and D2 receptor in the Vc. S22 Fig. Photomicrograph of CGRP-IR neurons in the trige [file pone.0323810.s001.zip › supplementary/Fig S29.tif]

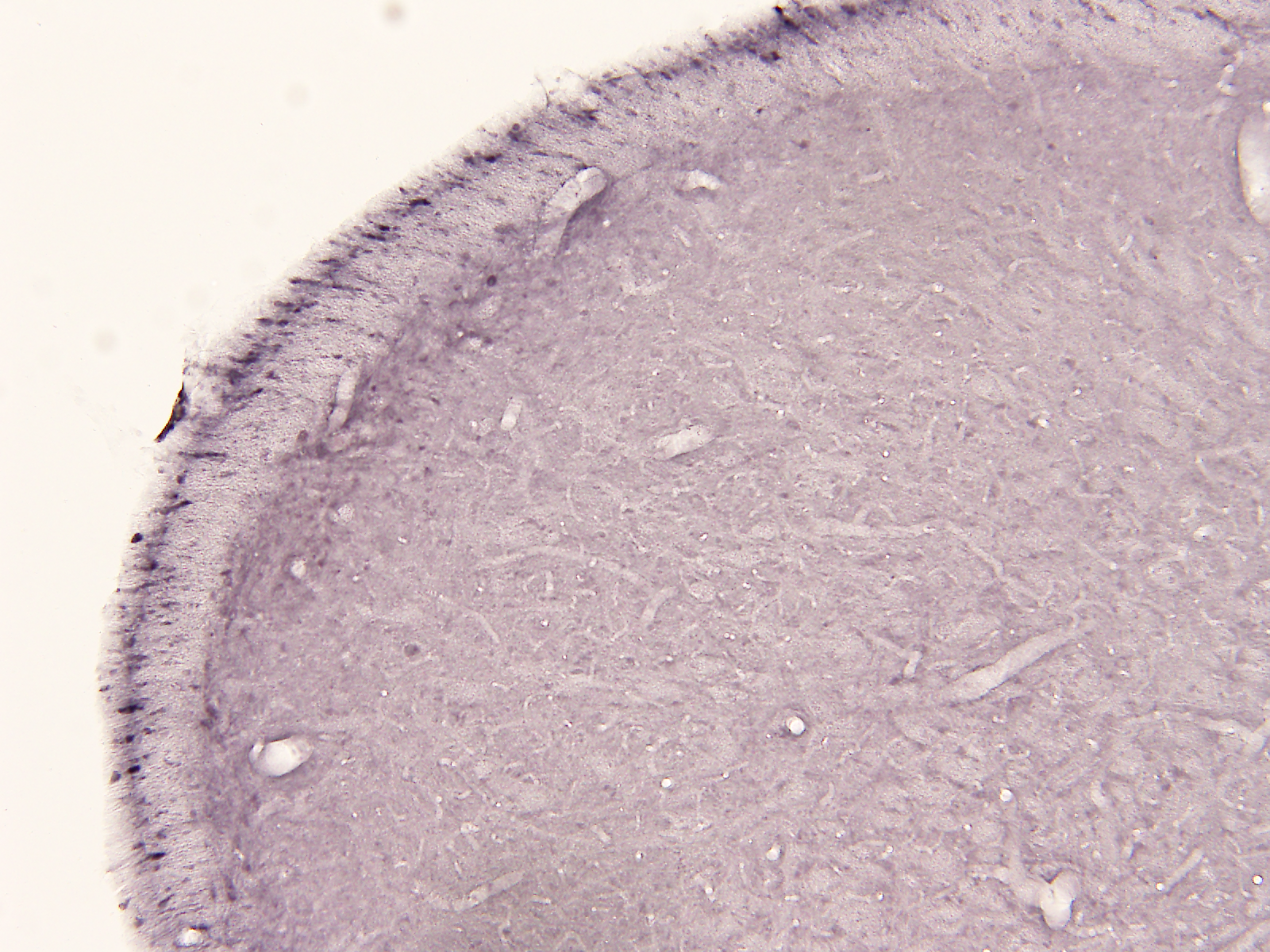

Supplement: S1 Data — S1 Fig. Photomicrograph of phosphorylated extracellular signal-regulated kinase (pERK)-immunoreactive (-IR) neurons in the trigeminal spinal subnucleus caudalis (Vc) of a sham rat. S2 Fig. Photomicrograph of pERK-IR neurons in the Vc of a chronic constriction injury of the infraorbital nerve (ION-CCI) rat. S3 Fig. Photomicrograph of pERK-IR neurons in the Vc of an ION-CCI rat receiving a vehicle. S4 Fig. Photomicrograph of pERK-IR neurons in the Vc of an ION-CCI rat receiving calcitonin gene-related peptide (CGRP). S5 Fig. Photomicrograph of pERK-IR neurons in the Vc of an ION-CCI rat receiving control immunogloublin G (IgG). S6 Fig. Photomicrograph of pERK-IR neurons in the Vc of an ION-CCI rat receiving an anti-CGRP antibody. S7 Fig. High magnification photomicrograph of pERK-IR neurons in the Vc of a sham rat. S8 Fig. High magnification photomicrograph of pERK-IR neurons in the Vc of an ION-CCI rat. S9 Fig. High magnification photomicrograph of pERK-IR neurons in the Vc of an ION-CCI rat receiving a vehicle. S10 Fig. High magnification photomicrograph of pERK-IR neurons in the Vc of an ION-CCI rat receiving CGRP. S11 Fig. High magnification photomicrograph of pERK-IR neurons in the Vc of an ION-CCI rat receiving control IgG. S12 Fig. High magnification photomicrograph of pERK-IR neurons in the Vc of an ION-CCI rat receiving an anti-CGRP antibody. S13 Fig. Immunofluorescent image of pERK in the Vc. S14 Fig. Immunofluorescent image of neuronal nuclei (NeuN) in the Vc. S15 Fig. Merged image of pERK and NeuN in the Vc. S16 Fig. Immunofluorescent image of pERK in the Vc. S17 Fig. Immunofluorescent image of dopamineD2 receptor (D2 receptor) in the Vc. S18 Fig. Merged image of pERK and D2 receptor in the Vc. S19 Fig. Immunofluorescent image of receptor activity modifying protein 1 (RAMP1) in the Vc. S20 Fig. Immunofluorescent image of D2 receptor in the Vc. S21 Fig. Merged image of RAMP1 and D2 receptor in the Vc. S22 Fig. Photomicrograph of CGRP-IR neurons in the trige [file pone.0323810.s001.zip › supplementary/Fig S3.TIF]

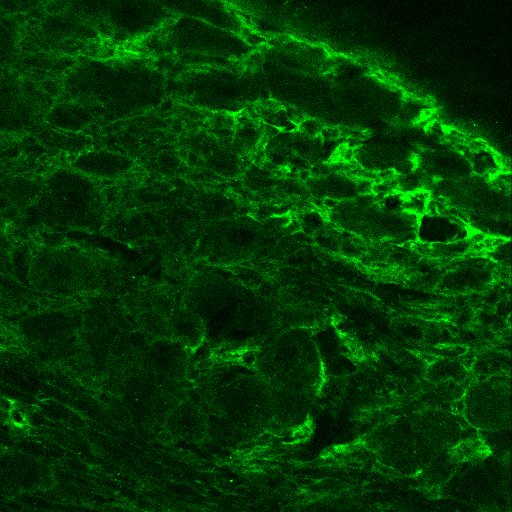

Supplement: S1 Data — S1 Fig. Photomicrograph of phosphorylated extracellular signal-regulated kinase (pERK)-immunoreactive (-IR) neurons in the trigeminal spinal subnucleus caudalis (Vc) of a sham rat. S2 Fig. Photomicrograph of pERK-IR neurons in the Vc of a chronic constriction injury of the infraorbital nerve (ION-CCI) rat. S3 Fig. Photomicrograph of pERK-IR neurons in the Vc of an ION-CCI rat receiving a vehicle. S4 Fig. Photomicrograph of pERK-IR neurons in the Vc of an ION-CCI rat receiving calcitonin gene-related peptide (CGRP). S5 Fig. Photomicrograph of pERK-IR neurons in the Vc of an ION-CCI rat receiving control immunogloublin G (IgG). S6 Fig. Photomicrograph of pERK-IR neurons in the Vc of an ION-CCI rat receiving an anti-CGRP antibody. S7 Fig. High magnification photomicrograph of pERK-IR neurons in the Vc of a sham rat. S8 Fig. High magnification photomicrograph of pERK-IR neurons in the Vc of an ION-CCI rat. S9 Fig. High magnification photomicrograph of pERK-IR neurons in the Vc of an ION-CCI rat receiving a vehicle. S10 Fig. High magnification photomicrograph of pERK-IR neurons in the Vc of an ION-CCI rat receiving CGRP. S11 Fig. High magnification photomicrograph of pERK-IR neurons in the Vc of an ION-CCI rat receiving control IgG. S12 Fig. High magnification photomicrograph of pERK-IR neurons in the Vc of an ION-CCI rat receiving an anti-CGRP antibody. S13 Fig. Immunofluorescent image of pERK in the Vc. S14 Fig. Immunofluorescent image of neuronal nuclei (NeuN) in the Vc. S15 Fig. Merged image of pERK and NeuN in the Vc. S16 Fig. Immunofluorescent image of pERK in the Vc. S17 Fig. Immunofluorescent image of dopamineD2 receptor (D2 receptor) in the Vc. S18 Fig. Merged image of pERK and D2 receptor in the Vc. S19 Fig. Immunofluorescent image of receptor activity modifying protein 1 (RAMP1) in the Vc. S20 Fig. Immunofluorescent image of D2 receptor in the Vc. S21 Fig. Merged image of RAMP1 and D2 receptor in the Vc. S22 Fig. Photomicrograph of CGRP-IR neurons in the trige [file pone.0323810.s001.zip › supplementary/Fig S30.tif]

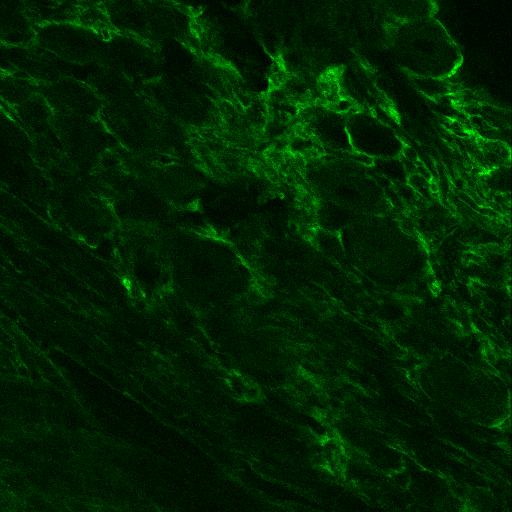

Supplement: S1 Data — S1 Fig. Photomicrograph of phosphorylated extracellular signal-regulated kinase (pERK)-immunoreactive (-IR) neurons in the trigeminal spinal subnucleus caudalis (Vc) of a sham rat. S2 Fig. Photomicrograph of pERK-IR neurons in the Vc of a chronic constriction injury of the infraorbital nerve (ION-CCI) rat. S3 Fig. Photomicrograph of pERK-IR neurons in the Vc of an ION-CCI rat receiving a vehicle. S4 Fig. Photomicrograph of pERK-IR neurons in the Vc of an ION-CCI rat receiving calcitonin gene-related peptide (CGRP). S5 Fig. Photomicrograph of pERK-IR neurons in the Vc of an ION-CCI rat receiving control immunogloublin G (IgG). S6 Fig. Photomicrograph of pERK-IR neurons in the Vc of an ION-CCI rat receiving an anti-CGRP antibody. S7 Fig. High magnification photomicrograph of pERK-IR neurons in the Vc of a sham rat. S8 Fig. High magnification photomicrograph of pERK-IR neurons in the Vc of an ION-CCI rat. S9 Fig. High magnification photomicrograph of pERK-IR neurons in the Vc of an ION-CCI rat receiving a vehicle. S10 Fig. High magnification photomicrograph of pERK-IR neurons in the Vc of an ION-CCI rat receiving CGRP. S11 Fig. High magnification photomicrograph of pERK-IR neurons in the Vc of an ION-CCI rat receiving control IgG. S12 Fig. High magnification photomicrograph of pERK-IR neurons in the Vc of an ION-CCI rat receiving an anti-CGRP antibody. S13 Fig. Immunofluorescent image of pERK in the Vc. S14 Fig. Immunofluorescent image of neuronal nuclei (NeuN) in the Vc. S15 Fig. Merged image of pERK and NeuN in the Vc. S16 Fig. Immunofluorescent image of pERK in the Vc. S17 Fig. Immunofluorescent image of dopamineD2 receptor (D2 receptor) in the Vc. S18 Fig. Merged image of pERK and D2 receptor in the Vc. S19 Fig. Immunofluorescent image of receptor activity modifying protein 1 (RAMP1) in the Vc. S20 Fig. Immunofluorescent image of D2 receptor in the Vc. S21 Fig. Merged image of RAMP1 and D2 receptor in the Vc. S22 Fig. Photomicrograph of CGRP-IR neurons in the trige [file pone.0323810.s001.zip › supplementary/Fig S31.tif]

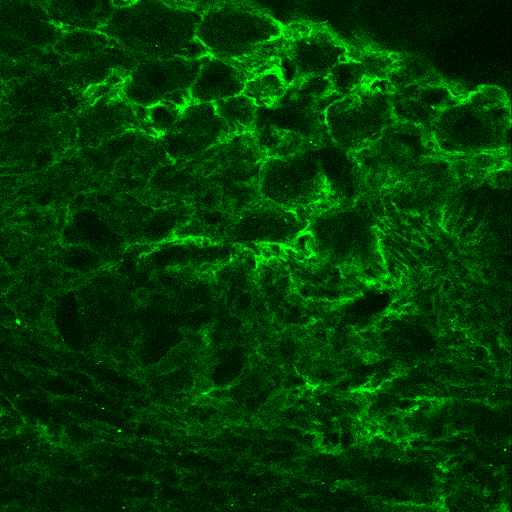

Supplement: S1 Data — S1 Fig. Photomicrograph of phosphorylated extracellular signal-regulated kinase (pERK)-immunoreactive (-IR) neurons in the trigeminal spinal subnucleus caudalis (Vc) of a sham rat. S2 Fig. Photomicrograph of pERK-IR neurons in the Vc of a chronic constriction injury of the infraorbital nerve (ION-CCI) rat. S3 Fig. Photomicrograph of pERK-IR neurons in the Vc of an ION-CCI rat receiving a vehicle. S4 Fig. Photomicrograph of pERK-IR neurons in the Vc of an ION-CCI rat receiving calcitonin gene-related peptide (CGRP). S5 Fig. Photomicrograph of pERK-IR neurons in the Vc of an ION-CCI rat receiving control immunogloublin G (IgG). S6 Fig. Photomicrograph of pERK-IR neurons in the Vc of an ION-CCI rat receiving an anti-CGRP antibody. S7 Fig. High magnification photomicrograph of pERK-IR neurons in the Vc of a sham rat. S8 Fig. High magnification photomicrograph of pERK-IR neurons in the Vc of an ION-CCI rat. S9 Fig. High magnification photomicrograph of pERK-IR neurons in the Vc of an ION-CCI rat receiving a vehicle. S10 Fig. High magnification photomicrograph of pERK-IR neurons in the Vc of an ION-CCI rat receiving CGRP. S11 Fig. High magnification photomicrograph of pERK-IR neurons in the Vc of an ION-CCI rat receiving control IgG. S12 Fig. High magnification photomicrograph of pERK-IR neurons in the Vc of an ION-CCI rat receiving an anti-CGRP antibody. S13 Fig. Immunofluorescent image of pERK in the Vc. S14 Fig. Immunofluorescent image of neuronal nuclei (NeuN) in the Vc. S15 Fig. Merged image of pERK and NeuN in the Vc. S16 Fig. Immunofluorescent image of pERK in the Vc. S17 Fig. Immunofluorescent image of dopamineD2 receptor (D2 receptor) in the Vc. S18 Fig. Merged image of pERK and D2 receptor in the Vc. S19 Fig. Immunofluorescent image of receptor activity modifying protein 1 (RAMP1) in the Vc. S20 Fig. Immunofluorescent image of D2 receptor in the Vc. S21 Fig. Merged image of RAMP1 and D2 receptor in the Vc. S22 Fig. Photomicrograph of CGRP-IR neurons in the trige [file pone.0323810.s001.zip › supplementary/Fig S32.tif]

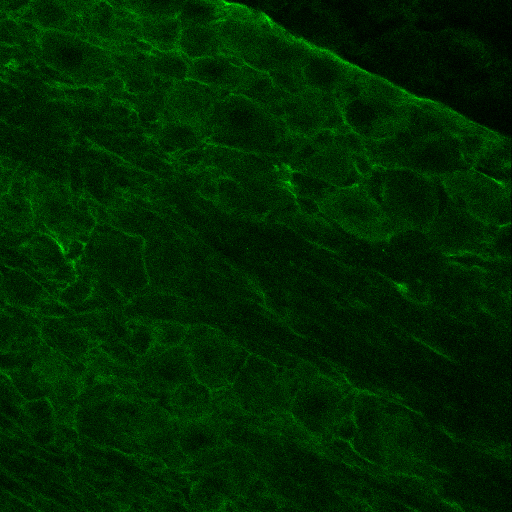

Supplement: S1 Data — S1 Fig. Photomicrograph of phosphorylated extracellular signal-regulated kinase (pERK)-immunoreactive (-IR) neurons in the trigeminal spinal subnucleus caudalis (Vc) of a sham rat. S2 Fig. Photomicrograph of pERK-IR neurons in the Vc of a chronic constriction injury of the infraorbital nerve (ION-CCI) rat. S3 Fig. Photomicrograph of pERK-IR neurons in the Vc of an ION-CCI rat receiving a vehicle. S4 Fig. Photomicrograph of pERK-IR neurons in the Vc of an ION-CCI rat receiving calcitonin gene-related peptide (CGRP). S5 Fig. Photomicrograph of pERK-IR neurons in the Vc of an ION-CCI rat receiving control immunogloublin G (IgG). S6 Fig. Photomicrograph of pERK-IR neurons in the Vc of an ION-CCI rat receiving an anti-CGRP antibody. S7 Fig. High magnification photomicrograph of pERK-IR neurons in the Vc of a sham rat. S8 Fig. High magnification photomicrograph of pERK-IR neurons in the Vc of an ION-CCI rat. S9 Fig. High magnification photomicrograph of pERK-IR neurons in the Vc of an ION-CCI rat receiving a vehicle. S10 Fig. High magnification photomicrograph of pERK-IR neurons in the Vc of an ION-CCI rat receiving CGRP. S11 Fig. High magnification photomicrograph of pERK-IR neurons in the Vc of an ION-CCI rat receiving control IgG. S12 Fig. High magnification photomicrograph of pERK-IR neurons in the Vc of an ION-CCI rat receiving an anti-CGRP antibody. S13 Fig. Immunofluorescent image of pERK in the Vc. S14 Fig. Immunofluorescent image of neuronal nuclei (NeuN) in the Vc. S15 Fig. Merged image of pERK and NeuN in the Vc. S16 Fig. Immunofluorescent image of pERK in the Vc. S17 Fig. Immunofluorescent image of dopamineD2 receptor (D2 receptor) in the Vc. S18 Fig. Merged image of pERK and D2 receptor in the Vc. S19 Fig. Immunofluorescent image of receptor activity modifying protein 1 (RAMP1) in the Vc. S20 Fig. Immunofluorescent image of D2 receptor in the Vc. S21 Fig. Merged image of RAMP1 and D2 receptor in the Vc. S22 Fig. Photomicrograph of CGRP-IR neurons in the trige [file pone.0323810.s001.zip › supplementary/Fig S33.tif]

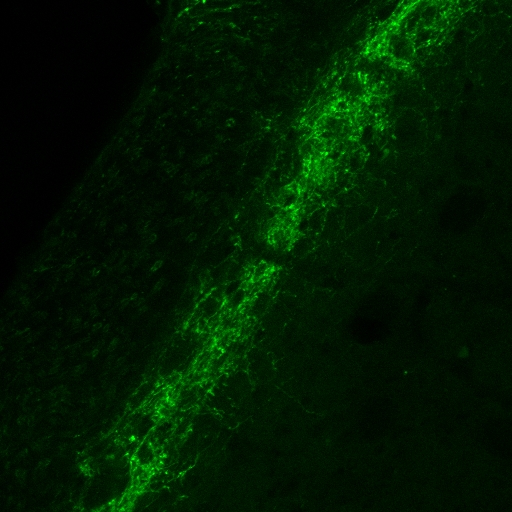

Supplement: S1 Data — S1 Fig. Photomicrograph of phosphorylated extracellular signal-regulated kinase (pERK)-immunoreactive (-IR) neurons in the trigeminal spinal subnucleus caudalis (Vc) of a sham rat. S2 Fig. Photomicrograph of pERK-IR neurons in the Vc of a chronic constriction injury of the infraorbital nerve (ION-CCI) rat. S3 Fig. Photomicrograph of pERK-IR neurons in the Vc of an ION-CCI rat receiving a vehicle. S4 Fig. Photomicrograph of pERK-IR neurons in the Vc of an ION-CCI rat receiving calcitonin gene-related peptide (CGRP). S5 Fig. Photomicrograph of pERK-IR neurons in the Vc of an ION-CCI rat receiving control immunogloublin G (IgG). S6 Fig. Photomicrograph of pERK-IR neurons in the Vc of an ION-CCI rat receiving an anti-CGRP antibody. S7 Fig. High magnification photomicrograph of pERK-IR neurons in the Vc of a sham rat. S8 Fig. High magnification photomicrograph of pERK-IR neurons in the Vc of an ION-CCI rat. S9 Fig. High magnification photomicrograph of pERK-IR neurons in the Vc of an ION-CCI rat receiving a vehicle. S10 Fig. High magnification photomicrograph of pERK-IR neurons in the Vc of an ION-CCI rat receiving CGRP. S11 Fig. High magnification photomicrograph of pERK-IR neurons in the Vc of an ION-CCI rat receiving control IgG. S12 Fig. High magnification photomicrograph of pERK-IR neurons in the Vc of an ION-CCI rat receiving an anti-CGRP antibody. S13 Fig. Immunofluorescent image of pERK in the Vc. S14 Fig. Immunofluorescent image of neuronal nuclei (NeuN) in the Vc. S15 Fig. Merged image of pERK and NeuN in the Vc. S16 Fig. Immunofluorescent image of pERK in the Vc. S17 Fig. Immunofluorescent image of dopamineD2 receptor (D2 receptor) in the Vc. S18 Fig. Merged image of pERK and D2 receptor in the Vc. S19 Fig. Immunofluorescent image of receptor activity modifying protein 1 (RAMP1) in the Vc. S20 Fig. Immunofluorescent image of D2 receptor in the Vc. S21 Fig. Merged image of RAMP1 and D2 receptor in the Vc. S22 Fig. Photomicrograph of CGRP-IR neurons in the trige [file pone.0323810.s001.zip › supplementary/Fig S34.tif]

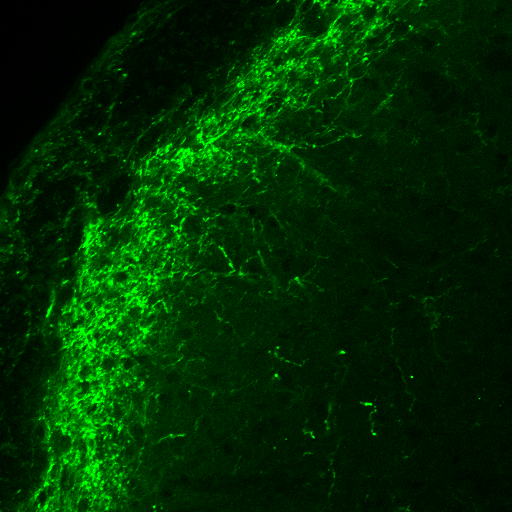

Supplement: S1 Data — S1 Fig. Photomicrograph of phosphorylated extracellular signal-regulated kinase (pERK)-immunoreactive (-IR) neurons in the trigeminal spinal subnucleus caudalis (Vc) of a sham rat. S2 Fig. Photomicrograph of pERK-IR neurons in the Vc of a chronic constriction injury of the infraorbital nerve (ION-CCI) rat. S3 Fig. Photomicrograph of pERK-IR neurons in the Vc of an ION-CCI rat receiving a vehicle. S4 Fig. Photomicrograph of pERK-IR neurons in the Vc of an ION-CCI rat receiving calcitonin gene-related peptide (CGRP). S5 Fig. Photomicrograph of pERK-IR neurons in the Vc of an ION-CCI rat receiving control immunogloublin G (IgG). S6 Fig. Photomicrograph of pERK-IR neurons in the Vc of an ION-CCI rat receiving an anti-CGRP antibody. S7 Fig. High magnification photomicrograph of pERK-IR neurons in the Vc of a sham rat. S8 Fig. High magnification photomicrograph of pERK-IR neurons in the Vc of an ION-CCI rat. S9 Fig. High magnification photomicrograph of pERK-IR neurons in the Vc of an ION-CCI rat receiving a vehicle. S10 Fig. High magnification photomicrograph of pERK-IR neurons in the Vc of an ION-CCI rat receiving CGRP. S11 Fig. High magnification photomicrograph of pERK-IR neurons in the Vc of an ION-CCI rat receiving control IgG. S12 Fig. High magnification photomicrograph of pERK-IR neurons in the Vc of an ION-CCI rat receiving an anti-CGRP antibody. S13 Fig. Immunofluorescent image of pERK in the Vc. S14 Fig. Immunofluorescent image of neuronal nuclei (NeuN) in the Vc. S15 Fig. Merged image of pERK and NeuN in the Vc. S16 Fig. Immunofluorescent image of pERK in the Vc. S17 Fig. Immunofluorescent image of dopamineD2 receptor (D2 receptor) in the Vc. S18 Fig. Merged image of pERK and D2 receptor in the Vc. S19 Fig. Immunofluorescent image of receptor activity modifying protein 1 (RAMP1) in the Vc. S20 Fig. Immunofluorescent image of D2 receptor in the Vc. S21 Fig. Merged image of RAMP1 and D2 receptor in the Vc. S22 Fig. Photomicrograph of CGRP-IR neurons in the trige [file pone.0323810.s001.zip › supplementary/Fig S35.tif]

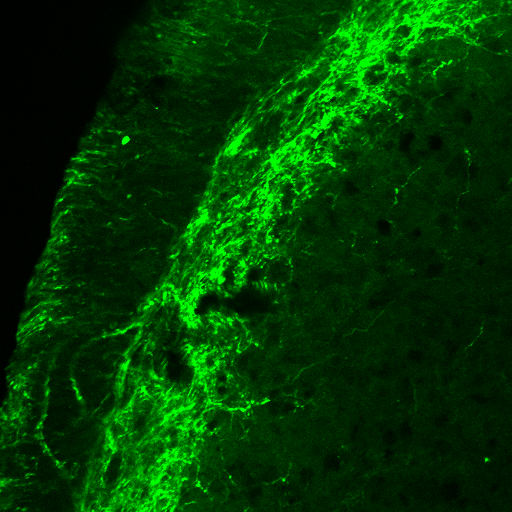

Supplement: S1 Data — S1 Fig. Photomicrograph of phosphorylated extracellular signal-regulated kinase (pERK)-immunoreactive (-IR) neurons in the trigeminal spinal subnucleus caudalis (Vc) of a sham rat. S2 Fig. Photomicrograph of pERK-IR neurons in the Vc of a chronic constriction injury of the infraorbital nerve (ION-CCI) rat. S3 Fig. Photomicrograph of pERK-IR neurons in the Vc of an ION-CCI rat receiving a vehicle. S4 Fig. Photomicrograph of pERK-IR neurons in the Vc of an ION-CCI rat receiving calcitonin gene-related peptide (CGRP). S5 Fig. Photomicrograph of pERK-IR neurons in the Vc of an ION-CCI rat receiving control immunogloublin G (IgG). S6 Fig. Photomicrograph of pERK-IR neurons in the Vc of an ION-CCI rat receiving an anti-CGRP antibody. S7 Fig. High magnification photomicrograph of pERK-IR neurons in the Vc of a sham rat. S8 Fig. High magnification photomicrograph of pERK-IR neurons in the Vc of an ION-CCI rat. S9 Fig. High magnification photomicrograph of pERK-IR neurons in the Vc of an ION-CCI rat receiving a vehicle. S10 Fig. High magnification photomicrograph of pERK-IR neurons in the Vc of an ION-CCI rat receiving CGRP. S11 Fig. High magnification photomicrograph of pERK-IR neurons in the Vc of an ION-CCI rat receiving control IgG. S12 Fig. High magnification photomicrograph of pERK-IR neurons in the Vc of an ION-CCI rat receiving an anti-CGRP antibody. S13 Fig. Immunofluorescent image of pERK in the Vc. S14 Fig. Immunofluorescent image of neuronal nuclei (NeuN) in the Vc. S15 Fig. Merged image of pERK and NeuN in the Vc. S16 Fig. Immunofluorescent image of pERK in the Vc. S17 Fig. Immunofluorescent image of dopamineD2 receptor (D2 receptor) in the Vc. S18 Fig. Merged image of pERK and D2 receptor in the Vc. S19 Fig. Immunofluorescent image of receptor activity modifying protein 1 (RAMP1) in the Vc. S20 Fig. Immunofluorescent image of D2 receptor in the Vc. S21 Fig. Merged image of RAMP1 and D2 receptor in the Vc. S22 Fig. Photomicrograph of CGRP-IR neurons in the trige [file pone.0323810.s001.zip › supplementary/Fig S36.tif]

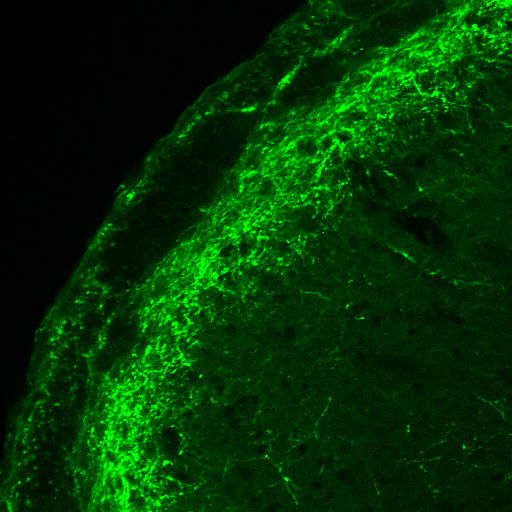

Supplement: S1 Data — S1 Fig. Photomicrograph of phosphorylated extracellular signal-regulated kinase (pERK)-immunoreactive (-IR) neurons in the trigeminal spinal subnucleus caudalis (Vc) of a sham rat. S2 Fig. Photomicrograph of pERK-IR neurons in the Vc of a chronic constriction injury of the infraorbital nerve (ION-CCI) rat. S3 Fig. Photomicrograph of pERK-IR neurons in the Vc of an ION-CCI rat receiving a vehicle. S4 Fig. Photomicrograph of pERK-IR neurons in the Vc of an ION-CCI rat receiving calcitonin gene-related peptide (CGRP). S5 Fig. Photomicrograph of pERK-IR neurons in the Vc of an ION-CCI rat receiving control immunogloublin G (IgG). S6 Fig. Photomicrograph of pERK-IR neurons in the Vc of an ION-CCI rat receiving an anti-CGRP antibody. S7 Fig. High magnification photomicrograph of pERK-IR neurons in the Vc of a sham rat. S8 Fig. High magnification photomicrograph of pERK-IR neurons in the Vc of an ION-CCI rat. S9 Fig. High magnification photomicrograph of pERK-IR neurons in the Vc of an ION-CCI rat receiving a vehicle. S10 Fig. High magnification photomicrograph of pERK-IR neurons in the Vc of an ION-CCI rat receiving CGRP. S11 Fig. High magnification photomicrograph of pERK-IR neurons in the Vc of an ION-CCI rat receiving control IgG. S12 Fig. High magnification photomicrograph of pERK-IR neurons in the Vc of an ION-CCI rat receiving an anti-CGRP antibody. S13 Fig. Immunofluorescent image of pERK in the Vc. S14 Fig. Immunofluorescent image of neuronal nuclei (NeuN) in the Vc. S15 Fig. Merged image of pERK and NeuN in the Vc. S16 Fig. Immunofluorescent image of pERK in the Vc. S17 Fig. Immunofluorescent image of dopamineD2 receptor (D2 receptor) in the Vc. S18 Fig. Merged image of pERK and D2 receptor in the Vc. S19 Fig. Immunofluorescent image of receptor activity modifying protein 1 (RAMP1) in the Vc. S20 Fig. Immunofluorescent image of D2 receptor in the Vc. S21 Fig. Merged image of RAMP1 and D2 receptor in the Vc. S22 Fig. Photomicrograph of CGRP-IR neurons in the trige [file pone.0323810.s001.zip › supplementary/Fig S37.tif]

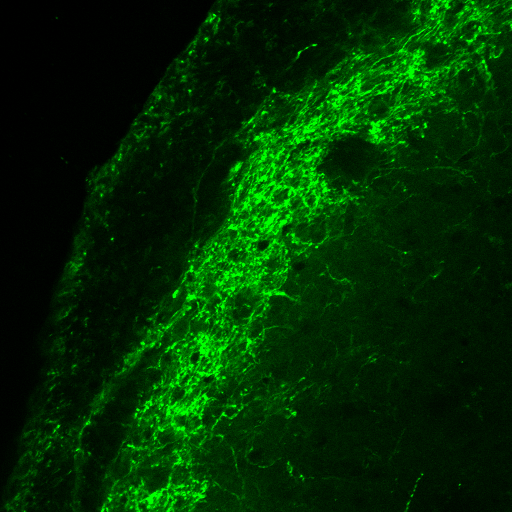

Supplement: S1 Data — S1 Fig. Photomicrograph of phosphorylated extracellular signal-regulated kinase (pERK)-immunoreactive (-IR) neurons in the trigeminal spinal subnucleus caudalis (Vc) of a sham rat. S2 Fig. Photomicrograph of pERK-IR neurons in the Vc of a chronic constriction injury of the infraorbital nerve (ION-CCI) rat. S3 Fig. Photomicrograph of pERK-IR neurons in the Vc of an ION-CCI rat receiving a vehicle. S4 Fig. Photomicrograph of pERK-IR neurons in the Vc of an ION-CCI rat receiving calcitonin gene-related peptide (CGRP). S5 Fig. Photomicrograph of pERK-IR neurons in the Vc of an ION-CCI rat receiving control immunogloublin G (IgG). S6 Fig. Photomicrograph of pERK-IR neurons in the Vc of an ION-CCI rat receiving an anti-CGRP antibody. S7 Fig. High magnification photomicrograph of pERK-IR neurons in the Vc of a sham rat. S8 Fig. High magnification photomicrograph of pERK-IR neurons in the Vc of an ION-CCI rat. S9 Fig. High magnification photomicrograph of pERK-IR neurons in the Vc of an ION-CCI rat receiving a vehicle. S10 Fig. High magnification photomicrograph of pERK-IR neurons in the Vc of an ION-CCI rat receiving CGRP. S11 Fig. High magnification photomicrograph of pERK-IR neurons in the Vc of an ION-CCI rat receiving control IgG. S12 Fig. High magnification photomicrograph of pERK-IR neurons in the Vc of an ION-CCI rat receiving an anti-CGRP antibody. S13 Fig. Immunofluorescent image of pERK in the Vc. S14 Fig. Immunofluorescent image of neuronal nuclei (NeuN) in the Vc. S15 Fig. Merged image of pERK and NeuN in the Vc. S16 Fig. Immunofluorescent image of pERK in the Vc. S17 Fig. Immunofluorescent image of dopamineD2 receptor (D2 receptor) in the Vc. S18 Fig. Merged image of pERK and D2 receptor in the Vc. S19 Fig. Immunofluorescent image of receptor activity modifying protein 1 (RAMP1) in the Vc. S20 Fig. Immunofluorescent image of D2 receptor in the Vc. S21 Fig. Merged image of RAMP1 and D2 receptor in the Vc. S22 Fig. Photomicrograph of CGRP-IR neurons in the trige [file pone.0323810.s001.zip › supplementary/Fig S38.tif]

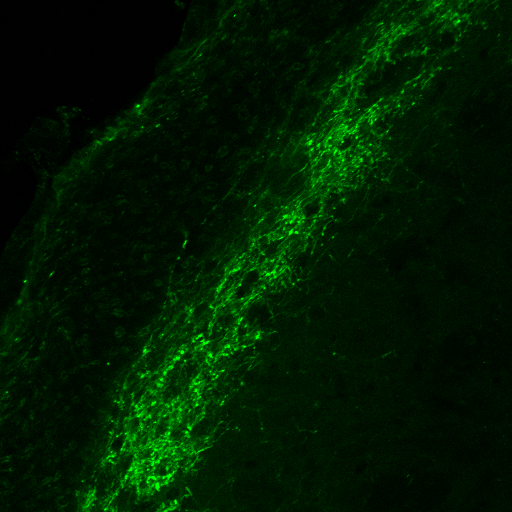

Supplement: S1 Data — S1 Fig. Photomicrograph of phosphorylated extracellular signal-regulated kinase (pERK)-immunoreactive (-IR) neurons in the trigeminal spinal subnucleus caudalis (Vc) of a sham rat. S2 Fig. Photomicrograph of pERK-IR neurons in the Vc of a chronic constriction injury of the infraorbital nerve (ION-CCI) rat. S3 Fig. Photomicrograph of pERK-IR neurons in the Vc of an ION-CCI rat receiving a vehicle. S4 Fig. Photomicrograph of pERK-IR neurons in the Vc of an ION-CCI rat receiving calcitonin gene-related peptide (CGRP). S5 Fig. Photomicrograph of pERK-IR neurons in the Vc of an ION-CCI rat receiving control immunogloublin G (IgG). S6 Fig. Photomicrograph of pERK-IR neurons in the Vc of an ION-CCI rat receiving an anti-CGRP antibody. S7 Fig. High magnification photomicrograph of pERK-IR neurons in the Vc of a sham rat. S8 Fig. High magnification photomicrograph of pERK-IR neurons in the Vc of an ION-CCI rat. S9 Fig. High magnification photomicrograph of pERK-IR neurons in the Vc of an ION-CCI rat receiving a vehicle. S10 Fig. High magnification photomicrograph of pERK-IR neurons in the Vc of an ION-CCI rat receiving CGRP. S11 Fig. High magnification photomicrograph of pERK-IR neurons in the Vc of an ION-CCI rat receiving control IgG. S12 Fig. High magnification photomicrograph of pERK-IR neurons in the Vc of an ION-CCI rat receiving an anti-CGRP antibody. S13 Fig. Immunofluorescent image of pERK in the Vc. S14 Fig. Immunofluorescent image of neuronal nuclei (NeuN) in the Vc. S15 Fig. Merged image of pERK and NeuN in the Vc. S16 Fig. Immunofluorescent image of pERK in the Vc. S17 Fig. Immunofluorescent image of dopamineD2 receptor (D2 receptor) in the Vc. S18 Fig. Merged image of pERK and D2 receptor in the Vc. S19 Fig. Immunofluorescent image of receptor activity modifying protein 1 (RAMP1) in the Vc. S20 Fig. Immunofluorescent image of D2 receptor in the Vc. S21 Fig. Merged image of RAMP1 and D2 receptor in the Vc. S22 Fig. Photomicrograph of CGRP-IR neurons in the trige [file pone.0323810.s001.zip › supplementary/Fig S39.tif]

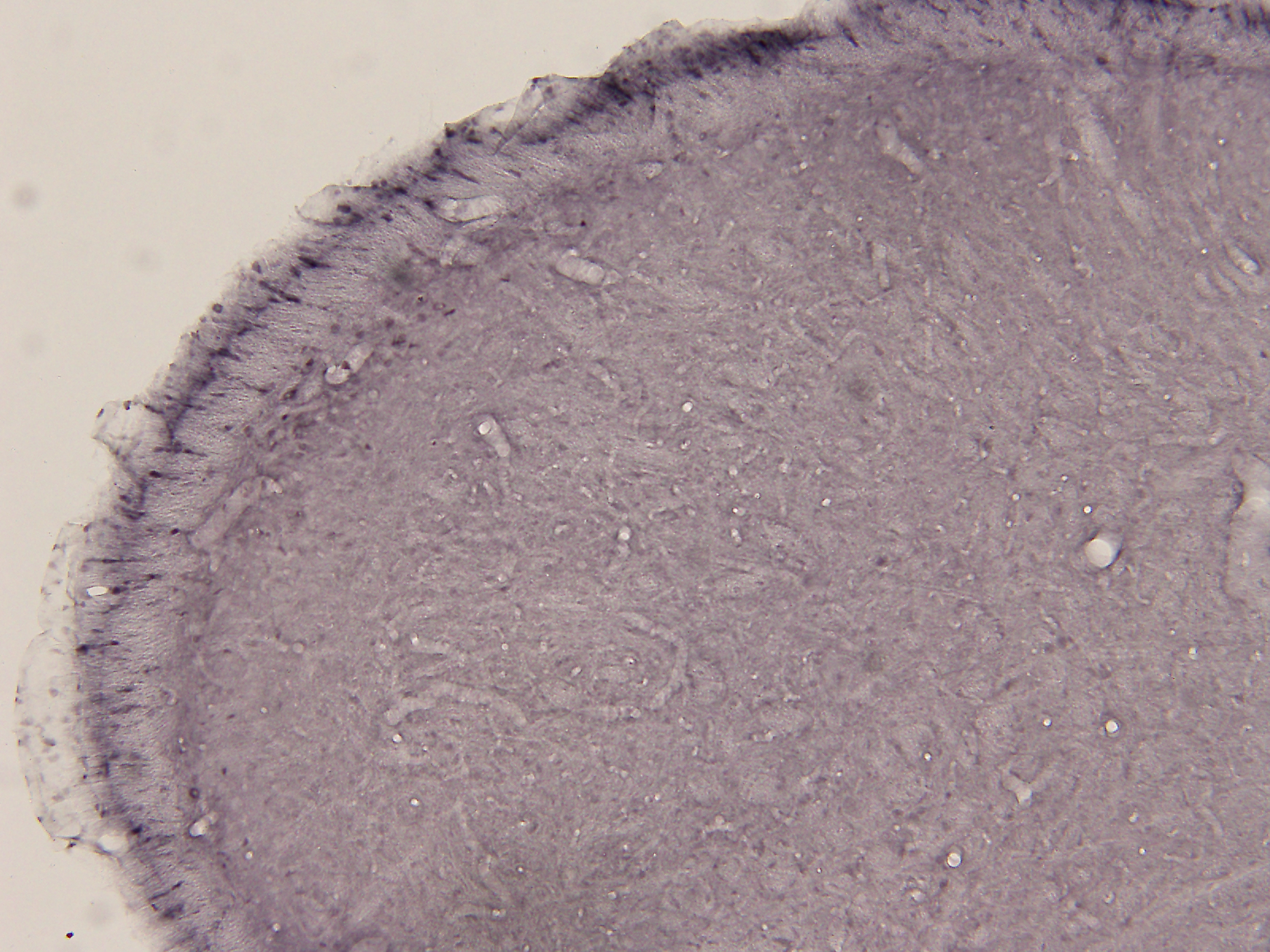

Supplement: S1 Data — S1 Fig. Photomicrograph of phosphorylated extracellular signal-regulated kinase (pERK)-immunoreactive (-IR) neurons in the trigeminal spinal subnucleus caudalis (Vc) of a sham rat. S2 Fig. Photomicrograph of pERK-IR neurons in the Vc of a chronic constriction injury of the infraorbital nerve (ION-CCI) rat. S3 Fig. Photomicrograph of pERK-IR neurons in the Vc of an ION-CCI rat receiving a vehicle. S4 Fig. Photomicrograph of pERK-IR neurons in the Vc of an ION-CCI rat receiving calcitonin gene-related peptide (CGRP). S5 Fig. Photomicrograph of pERK-IR neurons in the Vc of an ION-CCI rat receiving control immunogloublin G (IgG). S6 Fig. Photomicrograph of pERK-IR neurons in the Vc of an ION-CCI rat receiving an anti-CGRP antibody. S7 Fig. High magnification photomicrograph of pERK-IR neurons in the Vc of a sham rat. S8 Fig. High magnification photomicrograph of pERK-IR neurons in the Vc of an ION-CCI rat. S9 Fig. High magnification photomicrograph of pERK-IR neurons in the Vc of an ION-CCI rat receiving a vehicle. S10 Fig. High magnification photomicrograph of pERK-IR neurons in the Vc of an ION-CCI rat receiving CGRP. S11 Fig. High magnification photomicrograph of pERK-IR neurons in the Vc of an ION-CCI rat receiving control IgG. S12 Fig. High magnification photomicrograph of pERK-IR neurons in the Vc of an ION-CCI rat receiving an anti-CGRP antibody. S13 Fig. Immunofluorescent image of pERK in the Vc. S14 Fig. Immunofluorescent image of neuronal nuclei (NeuN) in the Vc. S15 Fig. Merged image of pERK and NeuN in the Vc. S16 Fig. Immunofluorescent image of pERK in the Vc. S17 Fig. Immunofluorescent image of dopamineD2 receptor (D2 receptor) in the Vc. S18 Fig. Merged image of pERK and D2 receptor in the Vc. S19 Fig. Immunofluorescent image of receptor activity modifying protein 1 (RAMP1) in the Vc. S20 Fig. Immunofluorescent image of D2 receptor in the Vc. S21 Fig. Merged image of RAMP1 and D2 receptor in the Vc. S22 Fig. Photomicrograph of CGRP-IR neurons in the trige [file pone.0323810.s001.zip › supplementary/Fig S4.TIF]

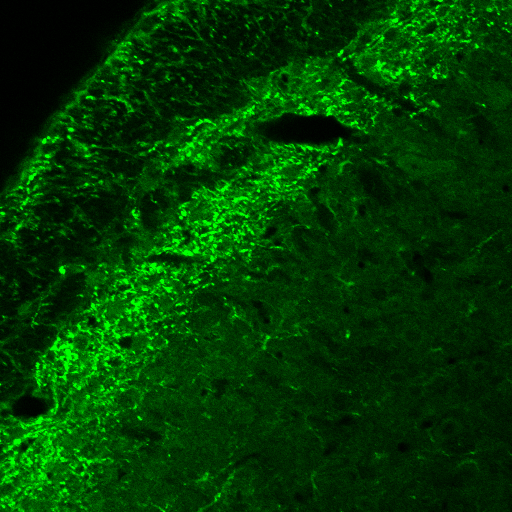

Supplement: S1 Data — S1 Fig. Photomicrograph of phosphorylated extracellular signal-regulated kinase (pERK)-immunoreactive (-IR) neurons in the trigeminal spinal subnucleus caudalis (Vc) of a sham rat. S2 Fig. Photomicrograph of pERK-IR neurons in the Vc of a chronic constriction injury of the infraorbital nerve (ION-CCI) rat. S3 Fig. Photomicrograph of pERK-IR neurons in the Vc of an ION-CCI rat receiving a vehicle. S4 Fig. Photomicrograph of pERK-IR neurons in the Vc of an ION-CCI rat receiving calcitonin gene-related peptide (CGRP). S5 Fig. Photomicrograph of pERK-IR neurons in the Vc of an ION-CCI rat receiving control immunogloublin G (IgG). S6 Fig. Photomicrograph of pERK-IR neurons in the Vc of an ION-CCI rat receiving an anti-CGRP antibody. S7 Fig. High magnification photomicrograph of pERK-IR neurons in the Vc of a sham rat. S8 Fig. High magnification photomicrograph of pERK-IR neurons in the Vc of an ION-CCI rat. S9 Fig. High magnification photomicrograph of pERK-IR neurons in the Vc of an ION-CCI rat receiving a vehicle. S10 Fig. High magnification photomicrograph of pERK-IR neurons in the Vc of an ION-CCI rat receiving CGRP. S11 Fig. High magnification photomicrograph of pERK-IR neurons in the Vc of an ION-CCI rat receiving control IgG. S12 Fig. High magnification photomicrograph of pERK-IR neurons in the Vc of an ION-CCI rat receiving an anti-CGRP antibody. S13 Fig. Immunofluorescent image of pERK in the Vc. S14 Fig. Immunofluorescent image of neuronal nuclei (NeuN) in the Vc. S15 Fig. Merged image of pERK and NeuN in the Vc. S16 Fig. Immunofluorescent image of pERK in the Vc. S17 Fig. Immunofluorescent image of dopamineD2 receptor (D2 receptor) in the Vc. S18 Fig. Merged image of pERK and D2 receptor in the Vc. S19 Fig. Immunofluorescent image of receptor activity modifying protein 1 (RAMP1) in the Vc. S20 Fig. Immunofluorescent image of D2 receptor in the Vc. S21 Fig. Merged image of RAMP1 and D2 receptor in the Vc. S22 Fig. Photomicrograph of CGRP-IR neurons in the trige [file pone.0323810.s001.zip › supplementary/Fig S40.tif]

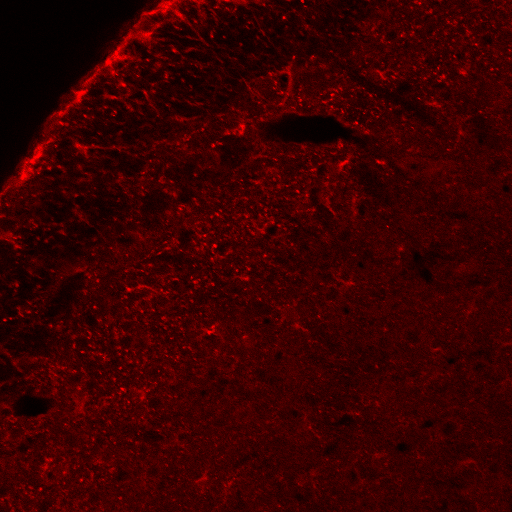

Supplement: S1 Data — S1 Fig. Photomicrograph of phosphorylated extracellular signal-regulated kinase (pERK)-immunoreactive (-IR) neurons in the trigeminal spinal subnucleus caudalis (Vc) of a sham rat. S2 Fig. Photomicrograph of pERK-IR neurons in the Vc of a chronic constriction injury of the infraorbital nerve (ION-CCI) rat. S3 Fig. Photomicrograph of pERK-IR neurons in the Vc of an ION-CCI rat receiving a vehicle. S4 Fig. Photomicrograph of pERK-IR neurons in the Vc of an ION-CCI rat receiving calcitonin gene-related peptide (CGRP). S5 Fig. Photomicrograph of pERK-IR neurons in the Vc of an ION-CCI rat receiving control immunogloublin G (IgG). S6 Fig. Photomicrograph of pERK-IR neurons in the Vc of an ION-CCI rat receiving an anti-CGRP antibody. S7 Fig. High magnification photomicrograph of pERK-IR neurons in the Vc of a sham rat. S8 Fig. High magnification photomicrograph of pERK-IR neurons in the Vc of an ION-CCI rat. S9 Fig. High magnification photomicrograph of pERK-IR neurons in the Vc of an ION-CCI rat receiving a vehicle. S10 Fig. High magnification photomicrograph of pERK-IR neurons in the Vc of an ION-CCI rat receiving CGRP. S11 Fig. High magnification photomicrograph of pERK-IR neurons in the Vc of an ION-CCI rat receiving control IgG. S12 Fig. High magnification photomicrograph of pERK-IR neurons in the Vc of an ION-CCI rat receiving an anti-CGRP antibody. S13 Fig. Immunofluorescent image of pERK in the Vc. S14 Fig. Immunofluorescent image of neuronal nuclei (NeuN) in the Vc. S15 Fig. Merged image of pERK and NeuN in the Vc. S16 Fig. Immunofluorescent image of pERK in the Vc. S17 Fig. Immunofluorescent image of dopamineD2 receptor (D2 receptor) in the Vc. S18 Fig. Merged image of pERK and D2 receptor in the Vc. S19 Fig. Immunofluorescent image of receptor activity modifying protein 1 (RAMP1) in the Vc. S20 Fig. Immunofluorescent image of D2 receptor in the Vc. S21 Fig. Merged image of RAMP1 and D2 receptor in the Vc. S22 Fig. Photomicrograph of CGRP-IR neurons in the trige [file pone.0323810.s001.zip › supplementary/Fig S41.tif]

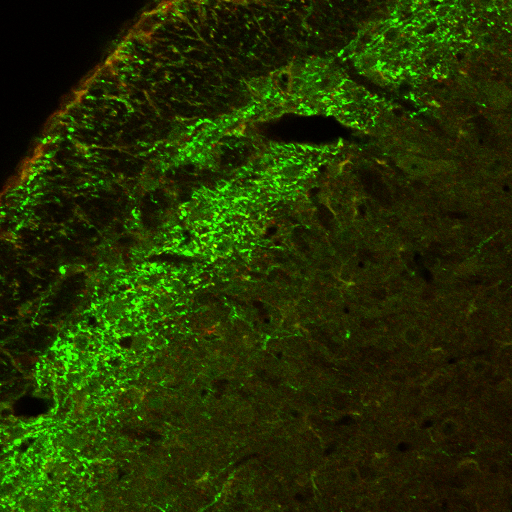

Supplement: S1 Data — S1 Fig. Photomicrograph of phosphorylated extracellular signal-regulated kinase (pERK)-immunoreactive (-IR) neurons in the trigeminal spinal subnucleus caudalis (Vc) of a sham rat. S2 Fig. Photomicrograph of pERK-IR neurons in the Vc of a chronic constriction injury of the infraorbital nerve (ION-CCI) rat. S3 Fig. Photomicrograph of pERK-IR neurons in the Vc of an ION-CCI rat receiving a vehicle. S4 Fig. Photomicrograph of pERK-IR neurons in the Vc of an ION-CCI rat receiving calcitonin gene-related peptide (CGRP). S5 Fig. Photomicrograph of pERK-IR neurons in the Vc of an ION-CCI rat receiving control immunogloublin G (IgG). S6 Fig. Photomicrograph of pERK-IR neurons in the Vc of an ION-CCI rat receiving an anti-CGRP antibody. S7 Fig. High magnification photomicrograph of pERK-IR neurons in the Vc of a sham rat. S8 Fig. High magnification photomicrograph of pERK-IR neurons in the Vc of an ION-CCI rat. S9 Fig. High magnification photomicrograph of pERK-IR neurons in the Vc of an ION-CCI rat receiving a vehicle. S10 Fig. High magnification photomicrograph of pERK-IR neurons in the Vc of an ION-CCI rat receiving CGRP. S11 Fig. High magnification photomicrograph of pERK-IR neurons in the Vc of an ION-CCI rat receiving control IgG. S12 Fig. High magnification photomicrograph of pERK-IR neurons in the Vc of an ION-CCI rat receiving an anti-CGRP antibody. S13 Fig. Immunofluorescent image of pERK in the Vc. S14 Fig. Immunofluorescent image of neuronal nuclei (NeuN) in the Vc. S15 Fig. Merged image of pERK and NeuN in the Vc. S16 Fig. Immunofluorescent image of pERK in the Vc. S17 Fig. Immunofluorescent image of dopamineD2 receptor (D2 receptor) in the Vc. S18 Fig. Merged image of pERK and D2 receptor in the Vc. S19 Fig. Immunofluorescent image of receptor activity modifying protein 1 (RAMP1) in the Vc. S20 Fig. Immunofluorescent image of D2 receptor in the Vc. S21 Fig. Merged image of RAMP1 and D2 receptor in the Vc. S22 Fig. Photomicrograph of CGRP-IR neurons in the trige [file pone.0323810.s001.zip › supplementary/Fig S42.tif]

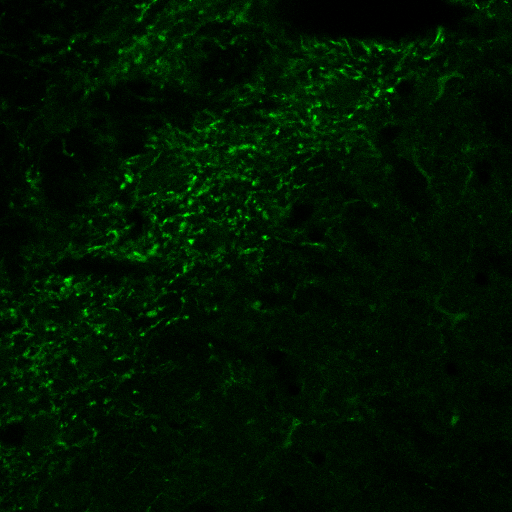

Supplement: S1 Data — S1 Fig. Photomicrograph of phosphorylated extracellular signal-regulated kinase (pERK)-immunoreactive (-IR) neurons in the trigeminal spinal subnucleus caudalis (Vc) of a sham rat. S2 Fig. Photomicrograph of pERK-IR neurons in the Vc of a chronic constriction injury of the infraorbital nerve (ION-CCI) rat. S3 Fig. Photomicrograph of pERK-IR neurons in the Vc of an ION-CCI rat receiving a vehicle. S4 Fig. Photomicrograph of pERK-IR neurons in the Vc of an ION-CCI rat receiving calcitonin gene-related peptide (CGRP). S5 Fig. Photomicrograph of pERK-IR neurons in the Vc of an ION-CCI rat receiving control immunogloublin G (IgG). S6 Fig. Photomicrograph of pERK-IR neurons in the Vc of an ION-CCI rat receiving an anti-CGRP antibody. S7 Fig. High magnification photomicrograph of pERK-IR neurons in the Vc of a sham rat. S8 Fig. High magnification photomicrograph of pERK-IR neurons in the Vc of an ION-CCI rat. S9 Fig. High magnification photomicrograph of pERK-IR neurons in the Vc of an ION-CCI rat receiving a vehicle. S10 Fig. High magnification photomicrograph of pERK-IR neurons in the Vc of an ION-CCI rat receiving CGRP. S11 Fig. High magnification photomicrograph of pERK-IR neurons in the Vc of an ION-CCI rat receiving control IgG. S12 Fig. High magnification photomicrograph of pERK-IR neurons in the Vc of an ION-CCI rat receiving an anti-CGRP antibody. S13 Fig. Immunofluorescent image of pERK in the Vc. S14 Fig. Immunofluorescent image of neuronal nuclei (NeuN) in the Vc. S15 Fig. Merged image of pERK and NeuN in the Vc. S16 Fig. Immunofluorescent image of pERK in the Vc. S17 Fig. Immunofluorescent image of dopamineD2 receptor (D2 receptor) in the Vc. S18 Fig. Merged image of pERK and D2 receptor in the Vc. S19 Fig. Immunofluorescent image of receptor activity modifying protein 1 (RAMP1) in the Vc. S20 Fig. Immunofluorescent image of D2 receptor in the Vc. S21 Fig. Merged image of RAMP1 and D2 receptor in the Vc. S22 Fig. Photomicrograph of CGRP-IR neurons in the trige [file pone.0323810.s001.zip › supplementary/Fig S43.tif]

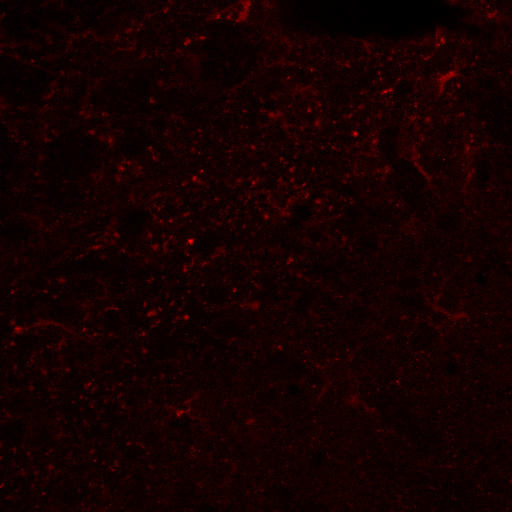

Supplement: S1 Data — S1 Fig. Photomicrograph of phosphorylated extracellular signal-regulated kinase (pERK)-immunoreactive (-IR) neurons in the trigeminal spinal subnucleus caudalis (Vc) of a sham rat. S2 Fig. Photomicrograph of pERK-IR neurons in the Vc of a chronic constriction injury of the infraorbital nerve (ION-CCI) rat. S3 Fig. Photomicrograph of pERK-IR neurons in the Vc of an ION-CCI rat receiving a vehicle. S4 Fig. Photomicrograph of pERK-IR neurons in the Vc of an ION-CCI rat receiving calcitonin gene-related peptide (CGRP). S5 Fig. Photomicrograph of pERK-IR neurons in the Vc of an ION-CCI rat receiving control immunogloublin G (IgG). S6 Fig. Photomicrograph of pERK-IR neurons in the Vc of an ION-CCI rat receiving an anti-CGRP antibody. S7 Fig. High magnification photomicrograph of pERK-IR neurons in the Vc of a sham rat. S8 Fig. High magnification photomicrograph of pERK-IR neurons in the Vc of an ION-CCI rat. S9 Fig. High magnification photomicrograph of pERK-IR neurons in the Vc of an ION-CCI rat receiving a vehicle. S10 Fig. High magnification photomicrograph of pERK-IR neurons in the Vc of an ION-CCI rat receiving CGRP. S11 Fig. High magnification photomicrograph of pERK-IR neurons in the Vc of an ION-CCI rat receiving control IgG. S12 Fig. High magnification photomicrograph of pERK-IR neurons in the Vc of an ION-CCI rat receiving an anti-CGRP antibody. S13 Fig. Immunofluorescent image of pERK in the Vc. S14 Fig. Immunofluorescent image of neuronal nuclei (NeuN) in the Vc. S15 Fig. Merged image of pERK and NeuN in the Vc. S16 Fig. Immunofluorescent image of pERK in the Vc. S17 Fig. Immunofluorescent image of dopamineD2 receptor (D2 receptor) in the Vc. S18 Fig. Merged image of pERK and D2 receptor in the Vc. S19 Fig. Immunofluorescent image of receptor activity modifying protein 1 (RAMP1) in the Vc. S20 Fig. Immunofluorescent image of D2 receptor in the Vc. S21 Fig. Merged image of RAMP1 and D2 receptor in the Vc. S22 Fig. Photomicrograph of CGRP-IR neurons in the trige [file pone.0323810.s001.zip › supplementary/Fig S44.tif]

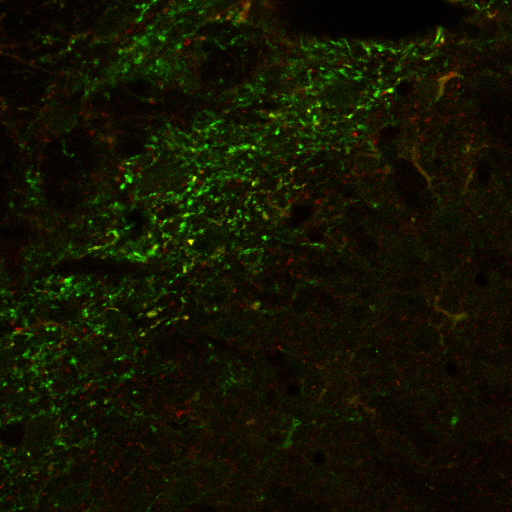

Supplement: S1 Data — S1 Fig. Photomicrograph of phosphorylated extracellular signal-regulated kinase (pERK)-immunoreactive (-IR) neurons in the trigeminal spinal subnucleus caudalis (Vc) of a sham rat. S2 Fig. Photomicrograph of pERK-IR neurons in the Vc of a chronic constriction injury of the infraorbital nerve (ION-CCI) rat. S3 Fig. Photomicrograph of pERK-IR neurons in the Vc of an ION-CCI rat receiving a vehicle. S4 Fig. Photomicrograph of pERK-IR neurons in the Vc of an ION-CCI rat receiving calcitonin gene-related peptide (CGRP). S5 Fig. Photomicrograph of pERK-IR neurons in the Vc of an ION-CCI rat receiving control immunogloublin G (IgG). S6 Fig. Photomicrograph of pERK-IR neurons in the Vc of an ION-CCI rat receiving an anti-CGRP antibody. S7 Fig. High magnification photomicrograph of pERK-IR neurons in the Vc of a sham rat. S8 Fig. High magnification photomicrograph of pERK-IR neurons in the Vc of an ION-CCI rat. S9 Fig. High magnification photomicrograph of pERK-IR neurons in the Vc of an ION-CCI rat receiving a vehicle. S10 Fig. High magnification photomicrograph of pERK-IR neurons in the Vc of an ION-CCI rat receiving CGRP. S11 Fig. High magnification photomicrograph of pERK-IR neurons in the Vc of an ION-CCI rat receiving control IgG. S12 Fig. High magnification photomicrograph of pERK-IR neurons in the Vc of an ION-CCI rat receiving an anti-CGRP antibody. S13 Fig. Immunofluorescent image of pERK in the Vc. S14 Fig. Immunofluorescent image of neuronal nuclei (NeuN) in the Vc. S15 Fig. Merged image of pERK and NeuN in the Vc. S16 Fig. Immunofluorescent image of pERK in the Vc. S17 Fig. Immunofluorescent image of dopamineD2 receptor (D2 receptor) in the Vc. S18 Fig. Merged image of pERK and D2 receptor in the Vc. S19 Fig. Immunofluorescent image of receptor activity modifying protein 1 (RAMP1) in the Vc. S20 Fig. Immunofluorescent image of D2 receptor in the Vc. S21 Fig. Merged image of RAMP1 and D2 receptor in the Vc. S22 Fig. Photomicrograph of CGRP-IR neurons in the trige [file pone.0323810.s001.zip › supplementary/Fig S45.tif]

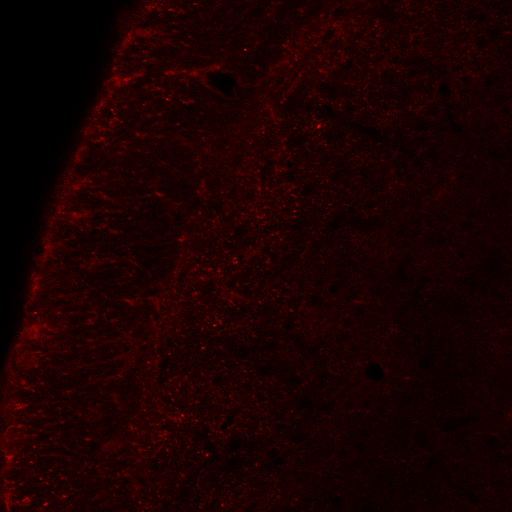

Supplement: S1 Data — S1 Fig. Photomicrograph of phosphorylated extracellular signal-regulated kinase (pERK)-immunoreactive (-IR) neurons in the trigeminal spinal subnucleus caudalis (Vc) of a sham rat. S2 Fig. Photomicrograph of pERK-IR neurons in the Vc of a chronic constriction injury of the infraorbital nerve (ION-CCI) rat. S3 Fig. Photomicrograph of pERK-IR neurons in the Vc of an ION-CCI rat receiving a vehicle. S4 Fig. Photomicrograph of pERK-IR neurons in the Vc of an ION-CCI rat receiving calcitonin gene-related peptide (CGRP). S5 Fig. Photomicrograph of pERK-IR neurons in the Vc of an ION-CCI rat receiving control immunogloublin G (IgG). S6 Fig. Photomicrograph of pERK-IR neurons in the Vc of an ION-CCI rat receiving an anti-CGRP antibody. S7 Fig. High magnification photomicrograph of pERK-IR neurons in the Vc of a sham rat. S8 Fig. High magnification photomicrograph of pERK-IR neurons in the Vc of an ION-CCI rat. S9 Fig. High magnification photomicrograph of pERK-IR neurons in the Vc of an ION-CCI rat receiving a vehicle. S10 Fig. High magnification photomicrograph of pERK-IR neurons in the Vc of an ION-CCI rat receiving CGRP. S11 Fig. High magnification photomicrograph of pERK-IR neurons in the Vc of an ION-CCI rat receiving control IgG. S12 Fig. High magnification photomicrograph of pERK-IR neurons in the Vc of an ION-CCI rat receiving an anti-CGRP antibody. S13 Fig. Immunofluorescent image of pERK in the Vc. S14 Fig. Immunofluorescent image of neuronal nuclei (NeuN) in the Vc. S15 Fig. Merged image of pERK and NeuN in the Vc. S16 Fig. Immunofluorescent image of pERK in the Vc. S17 Fig. Immunofluorescent image of dopamineD2 receptor (D2 receptor) in the Vc. S18 Fig. Merged image of pERK and D2 receptor in the Vc. S19 Fig. Immunofluorescent image of receptor activity modifying protein 1 (RAMP1) in the Vc. S20 Fig. Immunofluorescent image of D2 receptor in the Vc. S21 Fig. Merged image of RAMP1 and D2 receptor in the Vc. S22 Fig. Photomicrograph of CGRP-IR neurons in the trige [file pone.0323810.s001.zip › supplementary/Fig S46.tif]

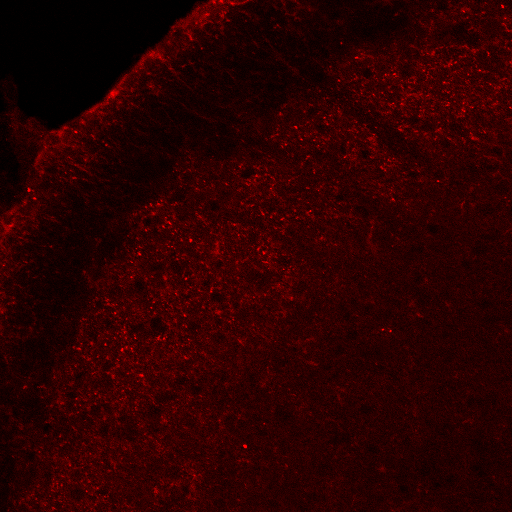

Supplement: S1 Data — S1 Fig. Photomicrograph of phosphorylated extracellular signal-regulated kinase (pERK)-immunoreactive (-IR) neurons in the trigeminal spinal subnucleus caudalis (Vc) of a sham rat. S2 Fig. Photomicrograph of pERK-IR neurons in the Vc of a chronic constriction injury of the infraorbital nerve (ION-CCI) rat. S3 Fig. Photomicrograph of pERK-IR neurons in the Vc of an ION-CCI rat receiving a vehicle. S4 Fig. Photomicrograph of pERK-IR neurons in the Vc of an ION-CCI rat receiving calcitonin gene-related peptide (CGRP). S5 Fig. Photomicrograph of pERK-IR neurons in the Vc of an ION-CCI rat receiving control immunogloublin G (IgG). S6 Fig. Photomicrograph of pERK-IR neurons in the Vc of an ION-CCI rat receiving an anti-CGRP antibody. S7 Fig. High magnification photomicrograph of pERK-IR neurons in the Vc of a sham rat. S8 Fig. High magnification photomicrograph of pERK-IR neurons in the Vc of an ION-CCI rat. S9 Fig. High magnification photomicrograph of pERK-IR neurons in the Vc of an ION-CCI rat receiving a vehicle. S10 Fig. High magnification photomicrograph of pERK-IR neurons in the Vc of an ION-CCI rat receiving CGRP. S11 Fig. High magnification photomicrograph of pERK-IR neurons in the Vc of an ION-CCI rat receiving control IgG. S12 Fig. High magnification photomicrograph of pERK-IR neurons in the Vc of an ION-CCI rat receiving an anti-CGRP antibody. S13 Fig. Immunofluorescent image of pERK in the Vc. S14 Fig. Immunofluorescent image of neuronal nuclei (NeuN) in the Vc. S15 Fig. Merged image of pERK and NeuN in the Vc. S16 Fig. Immunofluorescent image of pERK in the Vc. S17 Fig. Immunofluorescent image of dopamineD2 receptor (D2 receptor) in the Vc. S18 Fig. Merged image of pERK and D2 receptor in the Vc. S19 Fig. Immunofluorescent image of receptor activity modifying protein 1 (RAMP1) in the Vc. S20 Fig. Immunofluorescent image of D2 receptor in the Vc. S21 Fig. Merged image of RAMP1 and D2 receptor in the Vc. S22 Fig. Photomicrograph of CGRP-IR neurons in the trige [file pone.0323810.s001.zip › supplementary/Fig S47.tif]

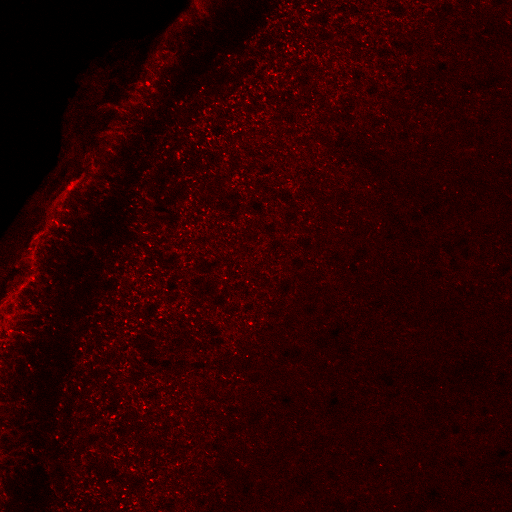

Supplement: S1 Data — S1 Fig. Photomicrograph of phosphorylated extracellular signal-regulated kinase (pERK)-immunoreactive (-IR) neurons in the trigeminal spinal subnucleus caudalis (Vc) of a sham rat. S2 Fig. Photomicrograph of pERK-IR neurons in the Vc of a chronic constriction injury of the infraorbital nerve (ION-CCI) rat. S3 Fig. Photomicrograph of pERK-IR neurons in the Vc of an ION-CCI rat receiving a vehicle. S4 Fig. Photomicrograph of pERK-IR neurons in the Vc of an ION-CCI rat receiving calcitonin gene-related peptide (CGRP). S5 Fig. Photomicrograph of pERK-IR neurons in the Vc of an ION-CCI rat receiving control immunogloublin G (IgG). S6 Fig. Photomicrograph of pERK-IR neurons in the Vc of an ION-CCI rat receiving an anti-CGRP antibody. S7 Fig. High magnification photomicrograph of pERK-IR neurons in the Vc of a sham rat. S8 Fig. High magnification photomicrograph of pERK-IR neurons in the Vc of an ION-CCI rat. S9 Fig. High magnification photomicrograph of pERK-IR neurons in the Vc of an ION-CCI rat receiving a vehicle. S10 Fig. High magnification photomicrograph of pERK-IR neurons in the Vc of an ION-CCI rat receiving CGRP. S11 Fig. High magnification photomicrograph of pERK-IR neurons in the Vc of an ION-CCI rat receiving control IgG. S12 Fig. High magnification photomicrograph of pERK-IR neurons in the Vc of an ION-CCI rat receiving an anti-CGRP antibody. S13 Fig. Immunofluorescent image of pERK in the Vc. S14 Fig. Immunofluorescent image of neuronal nuclei (NeuN) in the Vc. S15 Fig. Merged image of pERK and NeuN in the Vc. S16 Fig. Immunofluorescent image of pERK in the Vc. S17 Fig. Immunofluorescent image of dopamineD2 receptor (D2 receptor) in the Vc. S18 Fig. Merged image of pERK and D2 receptor in the Vc. S19 Fig. Immunofluorescent image of receptor activity modifying protein 1 (RAMP1) in the Vc. S20 Fig. Immunofluorescent image of D2 receptor in the Vc. S21 Fig. Merged image of RAMP1 and D2 receptor in the Vc. S22 Fig. Photomicrograph of CGRP-IR neurons in the trige [file pone.0323810.s001.zip › supplementary/Fig S48.tif]

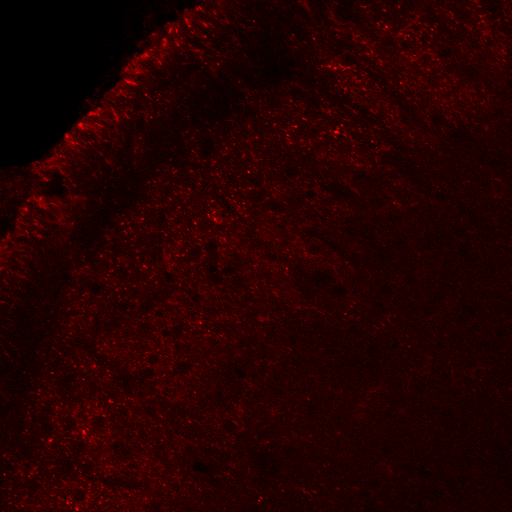

Supplement: S1 Data — S1 Fig. Photomicrograph of phosphorylated extracellular signal-regulated kinase (pERK)-immunoreactive (-IR) neurons in the trigeminal spinal subnucleus caudalis (Vc) of a sham rat. S2 Fig. Photomicrograph of pERK-IR neurons in the Vc of a chronic constriction injury of the infraorbital nerve (ION-CCI) rat. S3 Fig. Photomicrograph of pERK-IR neurons in the Vc of an ION-CCI rat receiving a vehicle. S4 Fig. Photomicrograph of pERK-IR neurons in the Vc of an ION-CCI rat receiving calcitonin gene-related peptide (CGRP). S5 Fig. Photomicrograph of pERK-IR neurons in the Vc of an ION-CCI rat receiving control immunogloublin G (IgG). S6 Fig. Photomicrograph of pERK-IR neurons in the Vc of an ION-CCI rat receiving an anti-CGRP antibody. S7 Fig. High magnification photomicrograph of pERK-IR neurons in the Vc of a sham rat. S8 Fig. High magnification photomicrograph of pERK-IR neurons in the Vc of an ION-CCI rat. S9 Fig. High magnification photomicrograph of pERK-IR neurons in the Vc of an ION-CCI rat receiving a vehicle. S10 Fig. High magnification photomicrograph of pERK-IR neurons in the Vc of an ION-CCI rat receiving CGRP. S11 Fig. High magnification photomicrograph of pERK-IR neurons in the Vc of an ION-CCI rat receiving control IgG. S12 Fig. High magnification photomicrograph of pERK-IR neurons in the Vc of an ION-CCI rat receiving an anti-CGRP antibody. S13 Fig. Immunofluorescent image of pERK in the Vc. S14 Fig. Immunofluorescent image of neuronal nuclei (NeuN) in the Vc. S15 Fig. Merged image of pERK and NeuN in the Vc. S16 Fig. Immunofluorescent image of pERK in the Vc. S17 Fig. Immunofluorescent image of dopamineD2 receptor (D2 receptor) in the Vc. S18 Fig. Merged image of pERK and D2 receptor in the Vc. S19 Fig. Immunofluorescent image of receptor activity modifying protein 1 (RAMP1) in the Vc. S20 Fig. Immunofluorescent image of D2 receptor in the Vc. S21 Fig. Merged image of RAMP1 and D2 receptor in the Vc. S22 Fig. Photomicrograph of CGRP-IR neurons in the trige [file pone.0323810.s001.zip › supplementary/Fig S49.tif]

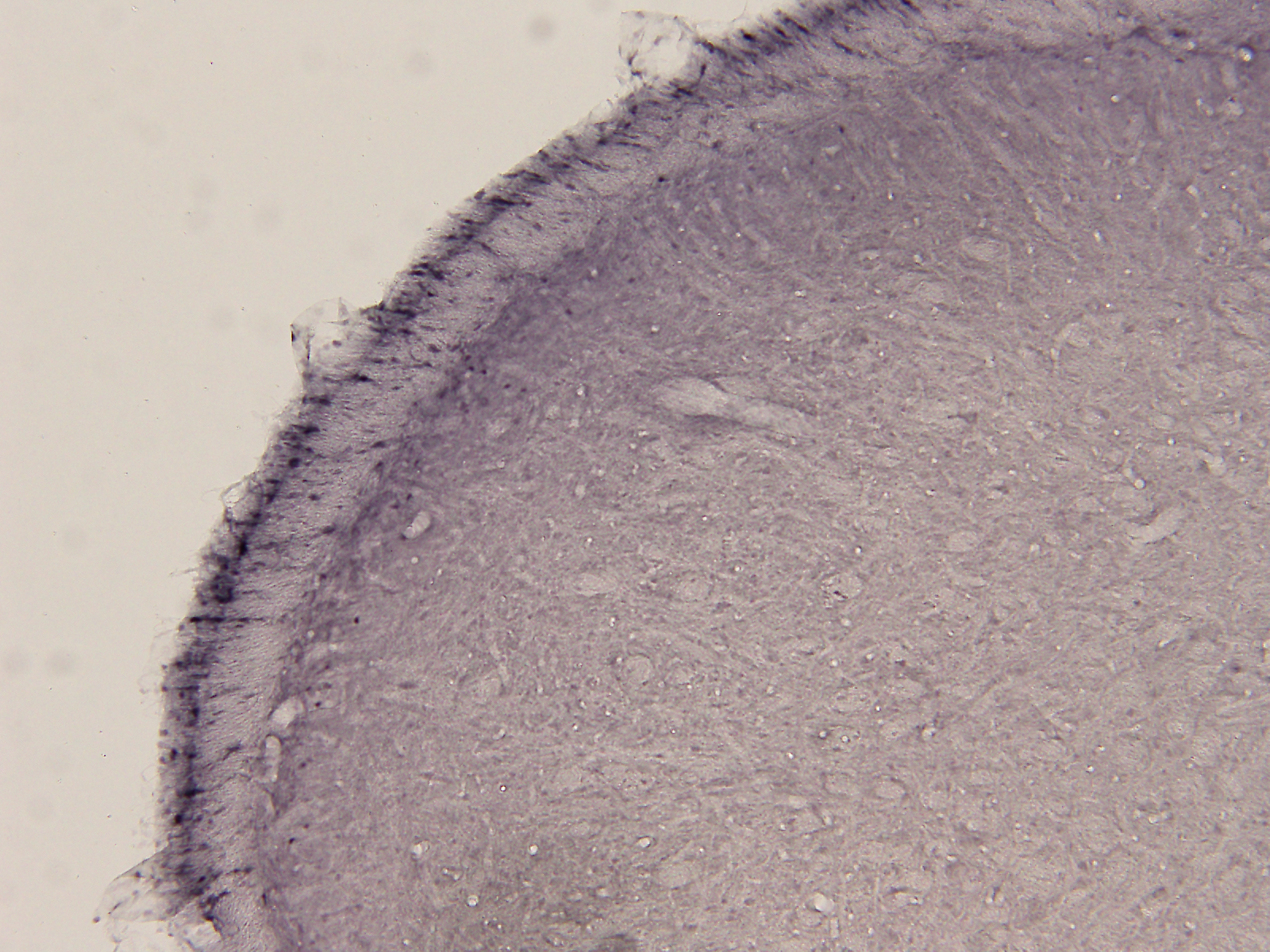

Supplement: S1 Data — S1 Fig. Photomicrograph of phosphorylated extracellular signal-regulated kinase (pERK)-immunoreactive (-IR) neurons in the trigeminal spinal subnucleus caudalis (Vc) of a sham rat. S2 Fig. Photomicrograph of pERK-IR neurons in the Vc of a chronic constriction injury of the infraorbital nerve (ION-CCI) rat. S3 Fig. Photomicrograph of pERK-IR neurons in the Vc of an ION-CCI rat receiving a vehicle. S4 Fig. Photomicrograph of pERK-IR neurons in the Vc of an ION-CCI rat receiving calcitonin gene-related peptide (CGRP). S5 Fig. Photomicrograph of pERK-IR neurons in the Vc of an ION-CCI rat receiving control immunogloublin G (IgG). S6 Fig. Photomicrograph of pERK-IR neurons in the Vc of an ION-CCI rat receiving an anti-CGRP antibody. S7 Fig. High magnification photomicrograph of pERK-IR neurons in the Vc of a sham rat. S8 Fig. High magnification photomicrograph of pERK-IR neurons in the Vc of an ION-CCI rat. S9 Fig. High magnification photomicrograph of pERK-IR neurons in the Vc of an ION-CCI rat receiving a vehicle. S10 Fig. High magnification photomicrograph of pERK-IR neurons in the Vc of an ION-CCI rat receiving CGRP. S11 Fig. High magnification photomicrograph of pERK-IR neurons in the Vc of an ION-CCI rat receiving control IgG. S12 Fig. High magnification photomicrograph of pERK-IR neurons in the Vc of an ION-CCI rat receiving an anti-CGRP antibody. S13 Fig. Immunofluorescent image of pERK in the Vc. S14 Fig. Immunofluorescent image of neuronal nuclei (NeuN) in the Vc. S15 Fig. Merged image of pERK and NeuN in the Vc. S16 Fig. Immunofluorescent image of pERK in the Vc. S17 Fig. Immunofluorescent image of dopamineD2 receptor (D2 receptor) in the Vc. S18 Fig. Merged image of pERK and D2 receptor in the Vc. S19 Fig. Immunofluorescent image of receptor activity modifying protein 1 (RAMP1) in the Vc. S20 Fig. Immunofluorescent image of D2 receptor in the Vc. S21 Fig. Merged image of RAMP1 and D2 receptor in the Vc. S22 Fig. Photomicrograph of CGRP-IR neurons in the trige [file pone.0323810.s001.zip › supplementary/Fig S5.TIF]

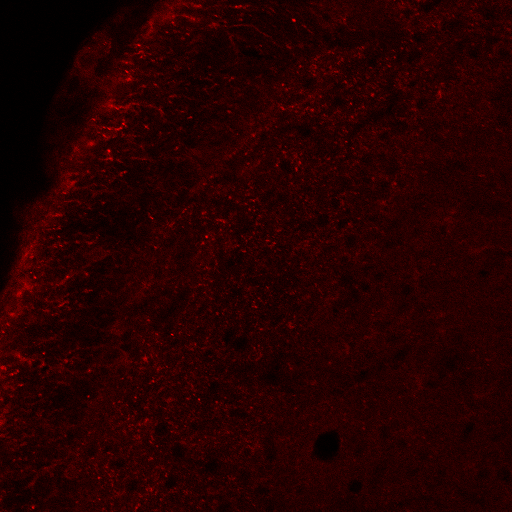

Supplement: S1 Data — S1 Fig. Photomicrograph of phosphorylated extracellular signal-regulated kinase (pERK)-immunoreactive (-IR) neurons in the trigeminal spinal subnucleus caudalis (Vc) of a sham rat. S2 Fig. Photomicrograph of pERK-IR neurons in the Vc of a chronic constriction injury of the infraorbital nerve (ION-CCI) rat. S3 Fig. Photomicrograph of pERK-IR neurons in the Vc of an ION-CCI rat receiving a vehicle. S4 Fig. Photomicrograph of pERK-IR neurons in the Vc of an ION-CCI rat receiving calcitonin gene-related peptide (CGRP). S5 Fig. Photomicrograph of pERK-IR neurons in the Vc of an ION-CCI rat receiving control immunogloublin G (IgG). S6 Fig. Photomicrograph of pERK-IR neurons in the Vc of an ION-CCI rat receiving an anti-CGRP antibody. S7 Fig. High magnification photomicrograph of pERK-IR neurons in the Vc of a sham rat. S8 Fig. High magnification photomicrograph of pERK-IR neurons in the Vc of an ION-CCI rat. S9 Fig. High magnification photomicrograph of pERK-IR neurons in the Vc of an ION-CCI rat receiving a vehicle. S10 Fig. High magnification photomicrograph of pERK-IR neurons in the Vc of an ION-CCI rat receiving CGRP. S11 Fig. High magnification photomicrograph of pERK-IR neurons in the Vc of an ION-CCI rat receiving control IgG. S12 Fig. High magnification photomicrograph of pERK-IR neurons in the Vc of an ION-CCI rat receiving an anti-CGRP antibody. S13 Fig. Immunofluorescent image of pERK in the Vc. S14 Fig. Immunofluorescent image of neuronal nuclei (NeuN) in the Vc. S15 Fig. Merged image of pERK and NeuN in the Vc. S16 Fig. Immunofluorescent image of pERK in the Vc. S17 Fig. Immunofluorescent image of dopamineD2 receptor (D2 receptor) in the Vc. S18 Fig. Merged image of pERK and D2 receptor in the Vc. S19 Fig. Immunofluorescent image of receptor activity modifying protein 1 (RAMP1) in the Vc. S20 Fig. Immunofluorescent image of D2 receptor in the Vc. S21 Fig. Merged image of RAMP1 and D2 receptor in the Vc. S22 Fig. Photomicrograph of CGRP-IR neurons in the trige [file pone.0323810.s001.zip › supplementary/Fig S50.tif]

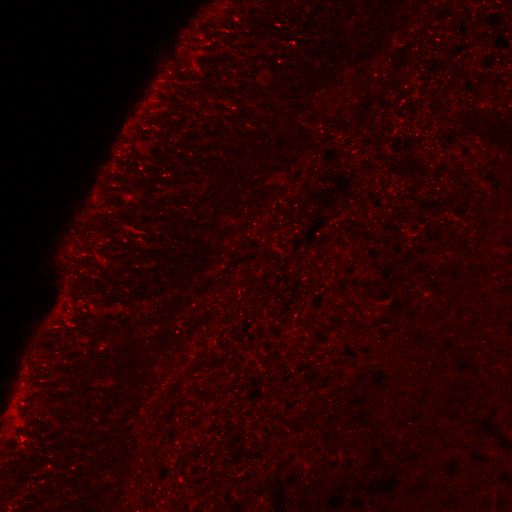

Supplement: S1 Data — S1 Fig. Photomicrograph of phosphorylated extracellular signal-regulated kinase (pERK)-immunoreactive (-IR) neurons in the trigeminal spinal subnucleus caudalis (Vc) of a sham rat. S2 Fig. Photomicrograph of pERK-IR neurons in the Vc of a chronic constriction injury of the infraorbital nerve (ION-CCI) rat. S3 Fig. Photomicrograph of pERK-IR neurons in the Vc of an ION-CCI rat receiving a vehicle. S4 Fig. Photomicrograph of pERK-IR neurons in the Vc of an ION-CCI rat receiving calcitonin gene-related peptide (CGRP). S5 Fig. Photomicrograph of pERK-IR neurons in the Vc of an ION-CCI rat receiving control immunogloublin G (IgG). S6 Fig. Photomicrograph of pERK-IR neurons in the Vc of an ION-CCI rat receiving an anti-CGRP antibody. S7 Fig. High magnification photomicrograph of pERK-IR neurons in the Vc of a sham rat. S8 Fig. High magnification photomicrograph of pERK-IR neurons in the Vc of an ION-CCI rat. S9 Fig. High magnification photomicrograph of pERK-IR neurons in the Vc of an ION-CCI rat receiving a vehicle. S10 Fig. High magnification photomicrograph of pERK-IR neurons in the Vc of an ION-CCI rat receiving CGRP. S11 Fig. High magnification photomicrograph of pERK-IR neurons in the Vc of an ION-CCI rat receiving control IgG. S12 Fig. High magnification photomicrograph of pERK-IR neurons in the Vc of an ION-CCI rat receiving an anti-CGRP antibody. S13 Fig. Immunofluorescent image of pERK in the Vc. S14 Fig. Immunofluorescent image of neuronal nuclei (NeuN) in the Vc. S15 Fig. Merged image of pERK and NeuN in the Vc. S16 Fig. Immunofluorescent image of pERK in the Vc. S17 Fig. Immunofluorescent image of dopamineD2 receptor (D2 receptor) in the Vc. S18 Fig. Merged image of pERK and D2 receptor in the Vc. S19 Fig. Immunofluorescent image of receptor activity modifying protein 1 (RAMP1) in the Vc. S20 Fig. Immunofluorescent image of D2 receptor in the Vc. S21 Fig. Merged image of RAMP1 and D2 receptor in the Vc. S22 Fig. Photomicrograph of CGRP-IR neurons in the trige [file pone.0323810.s001.zip › supplementary/Fig S51.tif]

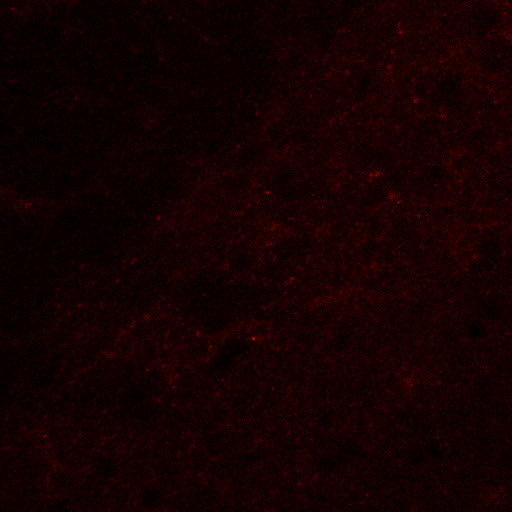

Supplement: S1 Data — S1 Fig. Photomicrograph of phosphorylated extracellular signal-regulated kinase (pERK)-immunoreactive (-IR) neurons in the trigeminal spinal subnucleus caudalis (Vc) of a sham rat. S2 Fig. Photomicrograph of pERK-IR neurons in the Vc of a chronic constriction injury of the infraorbital nerve (ION-CCI) rat. S3 Fig. Photomicrograph of pERK-IR neurons in the Vc of an ION-CCI rat receiving a vehicle. S4 Fig. Photomicrograph of pERK-IR neurons in the Vc of an ION-CCI rat receiving calcitonin gene-related peptide (CGRP). S5 Fig. Photomicrograph of pERK-IR neurons in the Vc of an ION-CCI rat receiving control immunogloublin G (IgG). S6 Fig. Photomicrograph of pERK-IR neurons in the Vc of an ION-CCI rat receiving an anti-CGRP antibody. S7 Fig. High magnification photomicrograph of pERK-IR neurons in the Vc of a sham rat. S8 Fig. High magnification photomicrograph of pERK-IR neurons in the Vc of an ION-CCI rat. S9 Fig. High magnification photomicrograph of pERK-IR neurons in the Vc of an ION-CCI rat receiving a vehicle. S10 Fig. High magnification photomicrograph of pERK-IR neurons in the Vc of an ION-CCI rat receiving CGRP. S11 Fig. High magnification photomicrograph of pERK-IR neurons in the Vc of an ION-CCI rat receiving control IgG. S12 Fig. High magnification photomicrograph of pERK-IR neurons in the Vc of an ION-CCI rat receiving an anti-CGRP antibody. S13 Fig. Immunofluorescent image of pERK in the Vc. S14 Fig. Immunofluorescent image of neuronal nuclei (NeuN) in the Vc. S15 Fig. Merged image of pERK and NeuN in the Vc. S16 Fig. Immunofluorescent image of pERK in the Vc. S17 Fig. Immunofluorescent image of dopamineD2 receptor (D2 receptor) in the Vc. S18 Fig. Merged image of pERK and D2 receptor in the Vc. S19 Fig. Immunofluorescent image of receptor activity modifying protein 1 (RAMP1) in the Vc. S20 Fig. Immunofluorescent image of D2 receptor in the Vc. S21 Fig. Merged image of RAMP1 and D2 receptor in the Vc. S22 Fig. Photomicrograph of CGRP-IR neurons in the trige [file pone.0323810.s001.zip › supplementary/Fig S52.tif]

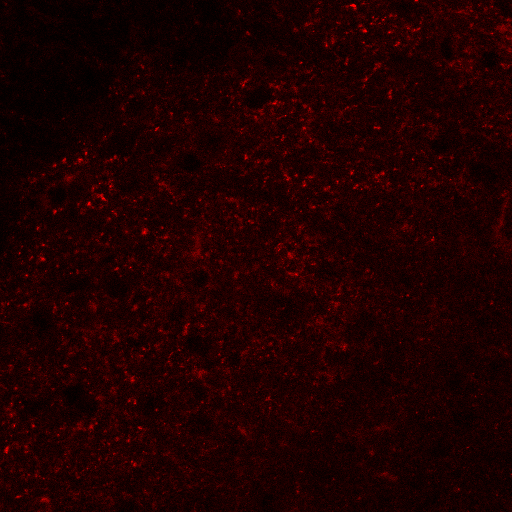

Supplement: S1 Data — S1 Fig. Photomicrograph of phosphorylated extracellular signal-regulated kinase (pERK)-immunoreactive (-IR) neurons in the trigeminal spinal subnucleus caudalis (Vc) of a sham rat. S2 Fig. Photomicrograph of pERK-IR neurons in the Vc of a chronic constriction injury of the infraorbital nerve (ION-CCI) rat. S3 Fig. Photomicrograph of pERK-IR neurons in the Vc of an ION-CCI rat receiving a vehicle. S4 Fig. Photomicrograph of pERK-IR neurons in the Vc of an ION-CCI rat receiving calcitonin gene-related peptide (CGRP). S5 Fig. Photomicrograph of pERK-IR neurons in the Vc of an ION-CCI rat receiving control immunogloublin G (IgG). S6 Fig. Photomicrograph of pERK-IR neurons in the Vc of an ION-CCI rat receiving an anti-CGRP antibody. S7 Fig. High magnification photomicrograph of pERK-IR neurons in the Vc of a sham rat. S8 Fig. High magnification photomicrograph of pERK-IR neurons in the Vc of an ION-CCI rat. S9 Fig. High magnification photomicrograph of pERK-IR neurons in the Vc of an ION-CCI rat receiving a vehicle. S10 Fig. High magnification photomicrograph of pERK-IR neurons in the Vc of an ION-CCI rat receiving CGRP. S11 Fig. High magnification photomicrograph of pERK-IR neurons in the Vc of an ION-CCI rat receiving control IgG. S12 Fig. High magnification photomicrograph of pERK-IR neurons in the Vc of an ION-CCI rat receiving an anti-CGRP antibody. S13 Fig. Immunofluorescent image of pERK in the Vc. S14 Fig. Immunofluorescent image of neuronal nuclei (NeuN) in the Vc. S15 Fig. Merged image of pERK and NeuN in the Vc. S16 Fig. Immunofluorescent image of pERK in the Vc. S17 Fig. Immunofluorescent image of dopamineD2 receptor (D2 receptor) in the Vc. S18 Fig. Merged image of pERK and D2 receptor in the Vc. S19 Fig. Immunofluorescent image of receptor activity modifying protein 1 (RAMP1) in the Vc. S20 Fig. Immunofluorescent image of D2 receptor in the Vc. S21 Fig. Merged image of RAMP1 and D2 receptor in the Vc. S22 Fig. Photomicrograph of CGRP-IR neurons in the trige [file pone.0323810.s001.zip › supplementary/Fig S53.tif]

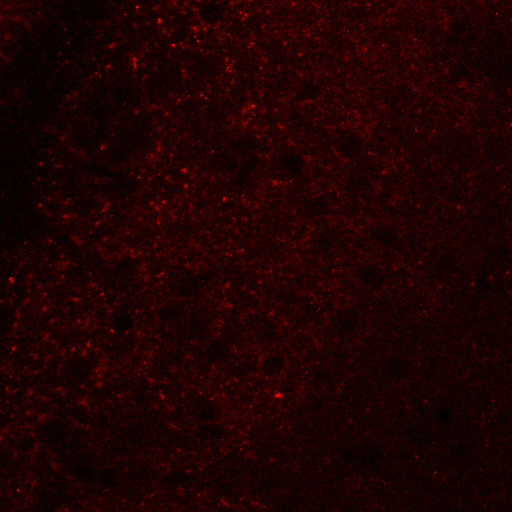

Supplement: S1 Data — S1 Fig. Photomicrograph of phosphorylated extracellular signal-regulated kinase (pERK)-immunoreactive (-IR) neurons in the trigeminal spinal subnucleus caudalis (Vc) of a sham rat. S2 Fig. Photomicrograph of pERK-IR neurons in the Vc of a chronic constriction injury of the infraorbital nerve (ION-CCI) rat. S3 Fig. Photomicrograph of pERK-IR neurons in the Vc of an ION-CCI rat receiving a vehicle. S4 Fig. Photomicrograph of pERK-IR neurons in the Vc of an ION-CCI rat receiving calcitonin gene-related peptide (CGRP). S5 Fig. Photomicrograph of pERK-IR neurons in the Vc of an ION-CCI rat receiving control immunogloublin G (IgG). S6 Fig. Photomicrograph of pERK-IR neurons in the Vc of an ION-CCI rat receiving an anti-CGRP antibody. S7 Fig. High magnification photomicrograph of pERK-IR neurons in the Vc of a sham rat. S8 Fig. High magnification photomicrograph of pERK-IR neurons in the Vc of an ION-CCI rat. S9 Fig. High magnification photomicrograph of pERK-IR neurons in the Vc of an ION-CCI rat receiving a vehicle. S10 Fig. High magnification photomicrograph of pERK-IR neurons in the Vc of an ION-CCI rat receiving CGRP. S11 Fig. High magnification photomicrograph of pERK-IR neurons in the Vc of an ION-CCI rat receiving control IgG. S12 Fig. High magnification photomicrograph of pERK-IR neurons in the Vc of an ION-CCI rat receiving an anti-CGRP antibody. S13 Fig. Immunofluorescent image of pERK in the Vc. S14 Fig. Immunofluorescent image of neuronal nuclei (NeuN) in the Vc. S15 Fig. Merged image of pERK and NeuN in the Vc. S16 Fig. Immunofluorescent image of pERK in the Vc. S17 Fig. Immunofluorescent image of dopamineD2 receptor (D2 receptor) in the Vc. S18 Fig. Merged image of pERK and D2 receptor in the Vc. S19 Fig. Immunofluorescent image of receptor activity modifying protein 1 (RAMP1) in the Vc. S20 Fig. Immunofluorescent image of D2 receptor in the Vc. S21 Fig. Merged image of RAMP1 and D2 receptor in the Vc. S22 Fig. Photomicrograph of CGRP-IR neurons in the trige [file pone.0323810.s001.zip › supplementary/Fig S54.tif]

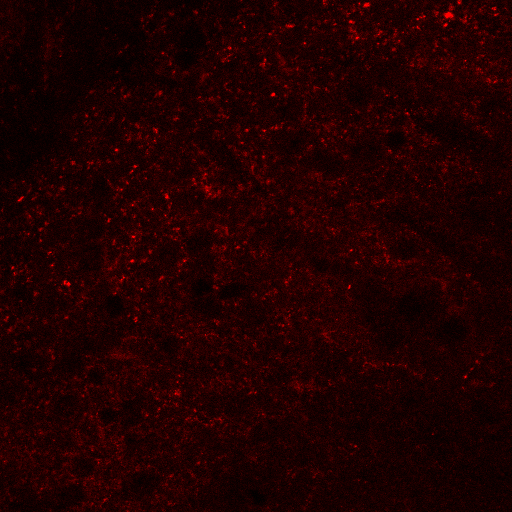

Supplement: S1 Data — S1 Fig. Photomicrograph of phosphorylated extracellular signal-regulated kinase (pERK)-immunoreactive (-IR) neurons in the trigeminal spinal subnucleus caudalis (Vc) of a sham rat. S2 Fig. Photomicrograph of pERK-IR neurons in the Vc of a chronic constriction injury of the infraorbital nerve (ION-CCI) rat. S3 Fig. Photomicrograph of pERK-IR neurons in the Vc of an ION-CCI rat receiving a vehicle. S4 Fig. Photomicrograph of pERK-IR neurons in the Vc of an ION-CCI rat receiving calcitonin gene-related peptide (CGRP). S5 Fig. Photomicrograph of pERK-IR neurons in the Vc of an ION-CCI rat receiving control immunogloublin G (IgG). S6 Fig. Photomicrograph of pERK-IR neurons in the Vc of an ION-CCI rat receiving an anti-CGRP antibody. S7 Fig. High magnification photomicrograph of pERK-IR neurons in the Vc of a sham rat. S8 Fig. High magnification photomicrograph of pERK-IR neurons in the Vc of an ION-CCI rat. S9 Fig. High magnification photomicrograph of pERK-IR neurons in the Vc of an ION-CCI rat receiving a vehicle. S10 Fig. High magnification photomicrograph of pERK-IR neurons in the Vc of an ION-CCI rat receiving CGRP. S11 Fig. High magnification photomicrograph of pERK-IR neurons in the Vc of an ION-CCI rat receiving control IgG. S12 Fig. High magnification photomicrograph of pERK-IR neurons in the Vc of an ION-CCI rat receiving an anti-CGRP antibody. S13 Fig. Immunofluorescent image of pERK in the Vc. S14 Fig. Immunofluorescent image of neuronal nuclei (NeuN) in the Vc. S15 Fig. Merged image of pERK and NeuN in the Vc. S16 Fig. Immunofluorescent image of pERK in the Vc. S17 Fig. Immunofluorescent image of dopamineD2 receptor (D2 receptor) in the Vc. S18 Fig. Merged image of pERK and D2 receptor in the Vc. S19 Fig. Immunofluorescent image of receptor activity modifying protein 1 (RAMP1) in the Vc. S20 Fig. Immunofluorescent image of D2 receptor in the Vc. S21 Fig. Merged image of RAMP1 and D2 receptor in the Vc. S22 Fig. Photomicrograph of CGRP-IR neurons in the trige [file pone.0323810.s001.zip › supplementary/Fig S55.tif]

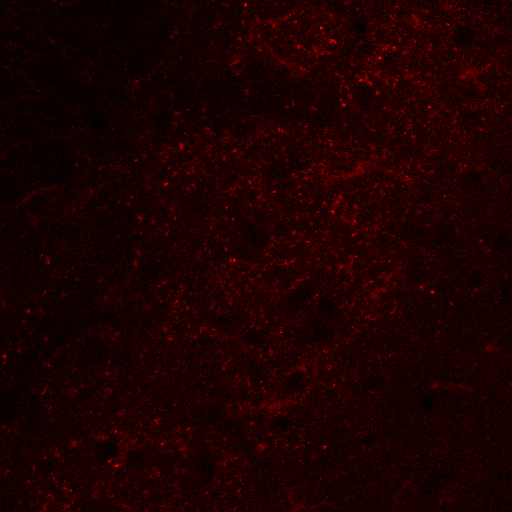

Supplement: S1 Data — S1 Fig. Photomicrograph of phosphorylated extracellular signal-regulated kinase (pERK)-immunoreactive (-IR) neurons in the trigeminal spinal subnucleus caudalis (Vc) of a sham rat. S2 Fig. Photomicrograph of pERK-IR neurons in the Vc of a chronic constriction injury of the infraorbital nerve (ION-CCI) rat. S3 Fig. Photomicrograph of pERK-IR neurons in the Vc of an ION-CCI rat receiving a vehicle. S4 Fig. Photomicrograph of pERK-IR neurons in the Vc of an ION-CCI rat receiving calcitonin gene-related peptide (CGRP). S5 Fig. Photomicrograph of pERK-IR neurons in the Vc of an ION-CCI rat receiving control immunogloublin G (IgG). S6 Fig. Photomicrograph of pERK-IR neurons in the Vc of an ION-CCI rat receiving an anti-CGRP antibody. S7 Fig. High magnification photomicrograph of pERK-IR neurons in the Vc of a sham rat. S8 Fig. High magnification photomicrograph of pERK-IR neurons in the Vc of an ION-CCI rat. S9 Fig. High magnification photomicrograph of pERK-IR neurons in the Vc of an ION-CCI rat receiving a vehicle. S10 Fig. High magnification photomicrograph of pERK-IR neurons in the Vc of an ION-CCI rat receiving CGRP. S11 Fig. High magnification photomicrograph of pERK-IR neurons in the Vc of an ION-CCI rat receiving control IgG. S12 Fig. High magnification photomicrograph of pERK-IR neurons in the Vc of an ION-CCI rat receiving an anti-CGRP antibody. S13 Fig. Immunofluorescent image of pERK in the Vc. S14 Fig. Immunofluorescent image of neuronal nuclei (NeuN) in the Vc. S15 Fig. Merged image of pERK and NeuN in the Vc. S16 Fig. Immunofluorescent image of pERK in the Vc. S17 Fig. Immunofluorescent image of dopamineD2 receptor (D2 receptor) in the Vc. S18 Fig. Merged image of pERK and D2 receptor in the Vc. S19 Fig. Immunofluorescent image of receptor activity modifying protein 1 (RAMP1) in the Vc. S20 Fig. Immunofluorescent image of D2 receptor in the Vc. S21 Fig. Merged image of RAMP1 and D2 receptor in the Vc. S22 Fig. Photomicrograph of CGRP-IR neurons in the trige [file pone.0323810.s001.zip › supplementary/Fig S56.tif]

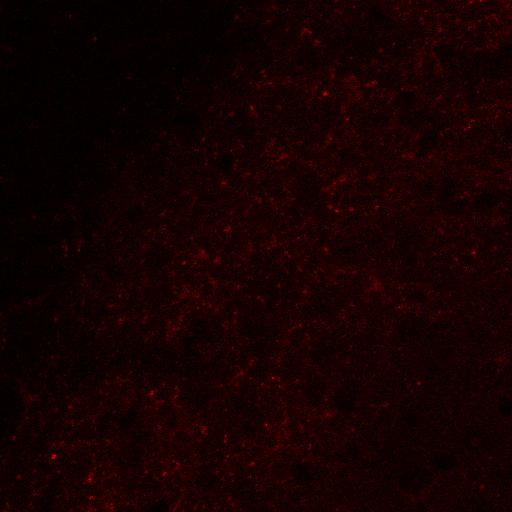

Supplement: S1 Data — S1 Fig. Photomicrograph of phosphorylated extracellular signal-regulated kinase (pERK)-immunoreactive (-IR) neurons in the trigeminal spinal subnucleus caudalis (Vc) of a sham rat. S2 Fig. Photomicrograph of pERK-IR neurons in the Vc of a chronic constriction injury of the infraorbital nerve (ION-CCI) rat. S3 Fig. Photomicrograph of pERK-IR neurons in the Vc of an ION-CCI rat receiving a vehicle. S4 Fig. Photomicrograph of pERK-IR neurons in the Vc of an ION-CCI rat receiving calcitonin gene-related peptide (CGRP). S5 Fig. Photomicrograph of pERK-IR neurons in the Vc of an ION-CCI rat receiving control immunogloublin G (IgG). S6 Fig. Photomicrograph of pERK-IR neurons in the Vc of an ION-CCI rat receiving an anti-CGRP antibody. S7 Fig. High magnification photomicrograph of pERK-IR neurons in the Vc of a sham rat. S8 Fig. High magnification photomicrograph of pERK-IR neurons in the Vc of an ION-CCI rat. S9 Fig. High magnification photomicrograph of pERK-IR neurons in the Vc of an ION-CCI rat receiving a vehicle. S10 Fig. High magnification photomicrograph of pERK-IR neurons in the Vc of an ION-CCI rat receiving CGRP. S11 Fig. High magnification photomicrograph of pERK-IR neurons in the Vc of an ION-CCI rat receiving control IgG. S12 Fig. High magnification photomicrograph of pERK-IR neurons in the Vc of an ION-CCI rat receiving an anti-CGRP antibody. S13 Fig. Immunofluorescent image of pERK in the Vc. S14 Fig. Immunofluorescent image of neuronal nuclei (NeuN) in the Vc. S15 Fig. Merged image of pERK and NeuN in the Vc. S16 Fig. Immunofluorescent image of pERK in the Vc. S17 Fig. Immunofluorescent image of dopamineD2 receptor (D2 receptor) in the Vc. S18 Fig. Merged image of pERK and D2 receptor in the Vc. S19 Fig. Immunofluorescent image of receptor activity modifying protein 1 (RAMP1) in the Vc. S20 Fig. Immunofluorescent image of D2 receptor in the Vc. S21 Fig. Merged image of RAMP1 and D2 receptor in the Vc. S22 Fig. Photomicrograph of CGRP-IR neurons in the trige [file pone.0323810.s001.zip › supplementary/Fig S57.tif]

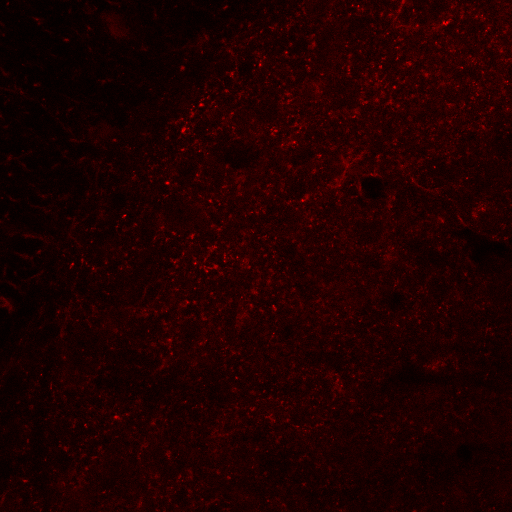

Supplement: S1 Data — S1 Fig. Photomicrograph of phosphorylated extracellular signal-regulated kinase (pERK)-immunoreactive (-IR) neurons in the trigeminal spinal subnucleus caudalis (Vc) of a sham rat. S2 Fig. Photomicrograph of pERK-IR neurons in the Vc of a chronic constriction injury of the infraorbital nerve (ION-CCI) rat. S3 Fig. Photomicrograph of pERK-IR neurons in the Vc of an ION-CCI rat receiving a vehicle. S4 Fig. Photomicrograph of pERK-IR neurons in the Vc of an ION-CCI rat receiving calcitonin gene-related peptide (CGRP). S5 Fig. Photomicrograph of pERK-IR neurons in the Vc of an ION-CCI rat receiving control immunogloublin G (IgG). S6 Fig. Photomicrograph of pERK-IR neurons in the Vc of an ION-CCI rat receiving an anti-CGRP antibody. S7 Fig. High magnification photomicrograph of pERK-IR neurons in the Vc of a sham rat. S8 Fig. High magnification photomicrograph of pERK-IR neurons in the Vc of an ION-CCI rat. S9 Fig. High magnification photomicrograph of pERK-IR neurons in the Vc of an ION-CCI rat receiving a vehicle. S10 Fig. High magnification photomicrograph of pERK-IR neurons in the Vc of an ION-CCI rat receiving CGRP. S11 Fig. High magnification photomicrograph of pERK-IR neurons in the Vc of an ION-CCI rat receiving control IgG. S12 Fig. High magnification photomicrograph of pERK-IR neurons in the Vc of an ION-CCI rat receiving an anti-CGRP antibody. S13 Fig. Immunofluorescent image of pERK in the Vc. S14 Fig. Immunofluorescent image of neuronal nuclei (NeuN) in the Vc. S15 Fig. Merged image of pERK and NeuN in the Vc. S16 Fig. Immunofluorescent image of pERK in the Vc. S17 Fig. Immunofluorescent image of dopamineD2 receptor (D2 receptor) in the Vc. S18 Fig. Merged image of pERK and D2 receptor in the Vc. S19 Fig. Immunofluorescent image of receptor activity modifying protein 1 (RAMP1) in the Vc. S20 Fig. Immunofluorescent image of D2 receptor in the Vc. S21 Fig. Merged image of RAMP1 and D2 receptor in the Vc. S22 Fig. Photomicrograph of CGRP-IR neurons in the trige [file pone.0323810.s001.zip › supplementary/Fig S58.tif]

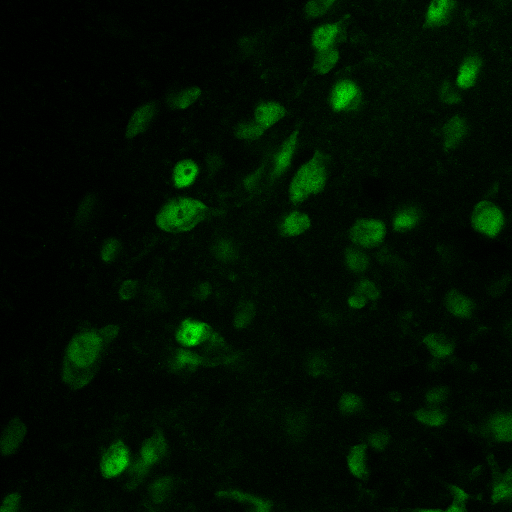

Supplement: S1 Data — S1 Fig. Photomicrograph of phosphorylated extracellular signal-regulated kinase (pERK)-immunoreactive (-IR) neurons in the trigeminal spinal subnucleus caudalis (Vc) of a sham rat. S2 Fig. Photomicrograph of pERK-IR neurons in the Vc of a chronic constriction injury of the infraorbital nerve (ION-CCI) rat. S3 Fig. Photomicrograph of pERK-IR neurons in the Vc of an ION-CCI rat receiving a vehicle. S4 Fig. Photomicrograph of pERK-IR neurons in the Vc of an ION-CCI rat receiving calcitonin gene-related peptide (CGRP). S5 Fig. Photomicrograph of pERK-IR neurons in the Vc of an ION-CCI rat receiving control immunogloublin G (IgG). S6 Fig. Photomicrograph of pERK-IR neurons in the Vc of an ION-CCI rat receiving an anti-CGRP antibody. S7 Fig. High magnification photomicrograph of pERK-IR neurons in the Vc of a sham rat. S8 Fig. High magnification photomicrograph of pERK-IR neurons in the Vc of an ION-CCI rat. S9 Fig. High magnification photomicrograph of pERK-IR neurons in the Vc of an ION-CCI rat receiving a vehicle. S10 Fig. High magnification photomicrograph of pERK-IR neurons in the Vc of an ION-CCI rat receiving CGRP. S11 Fig. High magnification photomicrograph of pERK-IR neurons in the Vc of an ION-CCI rat receiving control IgG. S12 Fig. High magnification photomicrograph of pERK-IR neurons in the Vc of an ION-CCI rat receiving an anti-CGRP antibody. S13 Fig. Immunofluorescent image of pERK in the Vc. S14 Fig. Immunofluorescent image of neuronal nuclei (NeuN) in the Vc. S15 Fig. Merged image of pERK and NeuN in the Vc. S16 Fig. Immunofluorescent image of pERK in the Vc. S17 Fig. Immunofluorescent image of dopamineD2 receptor (D2 receptor) in the Vc. S18 Fig. Merged image of pERK and D2 receptor in the Vc. S19 Fig. Immunofluorescent image of receptor activity modifying protein 1 (RAMP1) in the Vc. S20 Fig. Immunofluorescent image of D2 receptor in the Vc. S21 Fig. Merged image of RAMP1 and D2 receptor in the Vc. S22 Fig. Photomicrograph of CGRP-IR neurons in the trige [file pone.0323810.s001.zip › supplementary/Fig S59.tif]

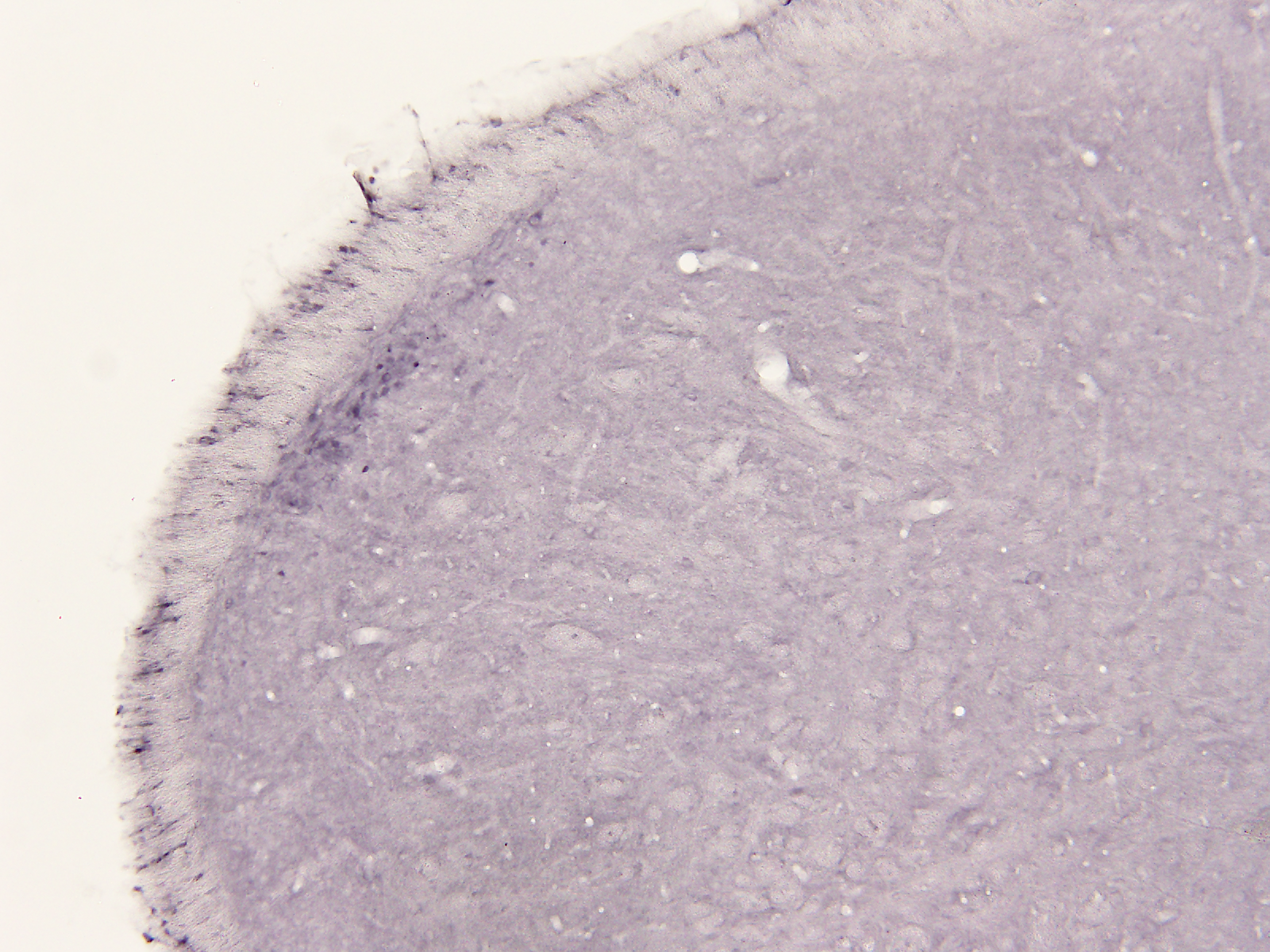

Supplement: S1 Data — S1 Fig. Photomicrograph of phosphorylated extracellular signal-regulated kinase (pERK)-immunoreactive (-IR) neurons in the trigeminal spinal subnucleus caudalis (Vc) of a sham rat. S2 Fig. Photomicrograph of pERK-IR neurons in the Vc of a chronic constriction injury of the infraorbital nerve (ION-CCI) rat. S3 Fig. Photomicrograph of pERK-IR neurons in the Vc of an ION-CCI rat receiving a vehicle. S4 Fig. Photomicrograph of pERK-IR neurons in the Vc of an ION-CCI rat receiving calcitonin gene-related peptide (CGRP). S5 Fig. Photomicrograph of pERK-IR neurons in the Vc of an ION-CCI rat receiving control immunogloublin G (IgG). S6 Fig. Photomicrograph of pERK-IR neurons in the Vc of an ION-CCI rat receiving an anti-CGRP antibody. S7 Fig. High magnification photomicrograph of pERK-IR neurons in the Vc of a sham rat. S8 Fig. High magnification photomicrograph of pERK-IR neurons in the Vc of an ION-CCI rat. S9 Fig. High magnification photomicrograph of pERK-IR neurons in the Vc of an ION-CCI rat receiving a vehicle. S10 Fig. High magnification photomicrograph of pERK-IR neurons in the Vc of an ION-CCI rat receiving CGRP. S11 Fig. High magnification photomicrograph of pERK-IR neurons in the Vc of an ION-CCI rat receiving control IgG. S12 Fig. High magnification photomicrograph of pERK-IR neurons in the Vc of an ION-CCI rat receiving an anti-CGRP antibody. S13 Fig. Immunofluorescent image of pERK in the Vc. S14 Fig. Immunofluorescent image of neuronal nuclei (NeuN) in the Vc. S15 Fig. Merged image of pERK and NeuN in the Vc. S16 Fig. Immunofluorescent image of pERK in the Vc. S17 Fig. Immunofluorescent image of dopamineD2 receptor (D2 receptor) in the Vc. S18 Fig. Merged image of pERK and D2 receptor in the Vc. S19 Fig. Immunofluorescent image of receptor activity modifying protein 1 (RAMP1) in the Vc. S20 Fig. Immunofluorescent image of D2 receptor in the Vc. S21 Fig. Merged image of RAMP1 and D2 receptor in the Vc. S22 Fig. Photomicrograph of CGRP-IR neurons in the trige [file pone.0323810.s001.zip › supplementary/Fig S6.TIF]

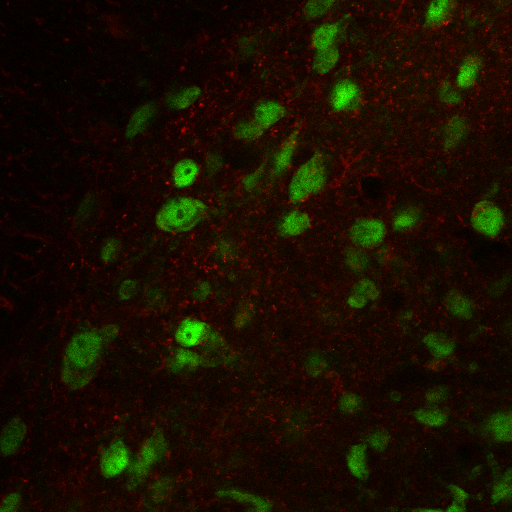

Supplement: S1 Data — S1 Fig. Photomicrograph of phosphorylated extracellular signal-regulated kinase (pERK)-immunoreactive (-IR) neurons in the trigeminal spinal subnucleus caudalis (Vc) of a sham rat. S2 Fig. Photomicrograph of pERK-IR neurons in the Vc of a chronic constriction injury of the infraorbital nerve (ION-CCI) rat. S3 Fig. Photomicrograph of pERK-IR neurons in the Vc of an ION-CCI rat receiving a vehicle. S4 Fig. Photomicrograph of pERK-IR neurons in the Vc of an ION-CCI rat receiving calcitonin gene-related peptide (CGRP). S5 Fig. Photomicrograph of pERK-IR neurons in the Vc of an ION-CCI rat receiving control immunogloublin G (IgG). S6 Fig. Photomicrograph of pERK-IR neurons in the Vc of an ION-CCI rat receiving an anti-CGRP antibody. S7 Fig. High magnification photomicrograph of pERK-IR neurons in the Vc of a sham rat. S8 Fig. High magnification photomicrograph of pERK-IR neurons in the Vc of an ION-CCI rat. S9 Fig. High magnification photomicrograph of pERK-IR neurons in the Vc of an ION-CCI rat receiving a vehicle. S10 Fig. High magnification photomicrograph of pERK-IR neurons in the Vc of an ION-CCI rat receiving CGRP. S11 Fig. High magnification photomicrograph of pERK-IR neurons in the Vc of an ION-CCI rat receiving control IgG. S12 Fig. High magnification photomicrograph of pERK-IR neurons in the Vc of an ION-CCI rat receiving an anti-CGRP antibody. S13 Fig. Immunofluorescent image of pERK in the Vc. S14 Fig. Immunofluorescent image of neuronal nuclei (NeuN) in the Vc. S15 Fig. Merged image of pERK and NeuN in the Vc. S16 Fig. Immunofluorescent image of pERK in the Vc. S17 Fig. Immunofluorescent image of dopamineD2 receptor (D2 receptor) in the Vc. S18 Fig. Merged image of pERK and D2 receptor in the Vc. S19 Fig. Immunofluorescent image of receptor activity modifying protein 1 (RAMP1) in the Vc. S20 Fig. Immunofluorescent image of D2 receptor in the Vc. S21 Fig. Merged image of RAMP1 and D2 receptor in the Vc. S22 Fig. Photomicrograph of CGRP-IR neurons in the trige [file pone.0323810.s001.zip › supplementary/Fig S60.tif]

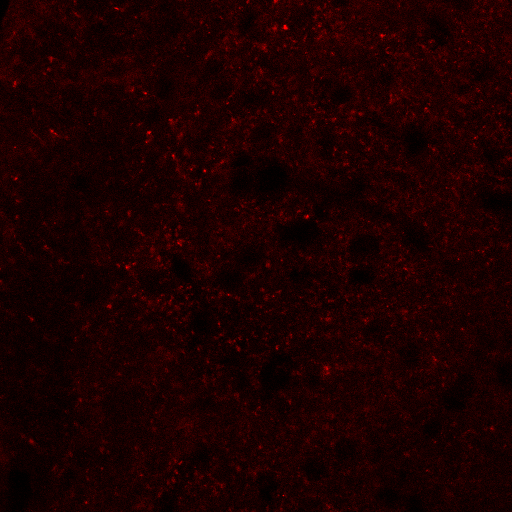

Supplement: S1 Data — S1 Fig. Photomicrograph of phosphorylated extracellular signal-regulated kinase (pERK)-immunoreactive (-IR) neurons in the trigeminal spinal subnucleus caudalis (Vc) of a sham rat. S2 Fig. Photomicrograph of pERK-IR neurons in the Vc of a chronic constriction injury of the infraorbital nerve (ION-CCI) rat. S3 Fig. Photomicrograph of pERK-IR neurons in the Vc of an ION-CCI rat receiving a vehicle. S4 Fig. Photomicrograph of pERK-IR neurons in the Vc of an ION-CCI rat receiving calcitonin gene-related peptide (CGRP). S5 Fig. Photomicrograph of pERK-IR neurons in the Vc of an ION-CCI rat receiving control immunogloublin G (IgG). S6 Fig. Photomicrograph of pERK-IR neurons in the Vc of an ION-CCI rat receiving an anti-CGRP antibody. S7 Fig. High magnification photomicrograph of pERK-IR neurons in the Vc of a sham rat. S8 Fig. High magnification photomicrograph of pERK-IR neurons in the Vc of an ION-CCI rat. S9 Fig. High magnification photomicrograph of pERK-IR neurons in the Vc of an ION-CCI rat receiving a vehicle. S10 Fig. High magnification photomicrograph of pERK-IR neurons in the Vc of an ION-CCI rat receiving CGRP. S11 Fig. High magnification photomicrograph of pERK-IR neurons in the Vc of an ION-CCI rat receiving control IgG. S12 Fig. High magnification photomicrograph of pERK-IR neurons in the Vc of an ION-CCI rat receiving an anti-CGRP antibody. S13 Fig. Immunofluorescent image of pERK in the Vc. S14 Fig. Immunofluorescent image of neuronal nuclei (NeuN) in the Vc. S15 Fig. Merged image of pERK and NeuN in the Vc. S16 Fig. Immunofluorescent image of pERK in the Vc. S17 Fig. Immunofluorescent image of dopamineD2 receptor (D2 receptor) in the Vc. S18 Fig. Merged image of pERK and D2 receptor in the Vc. S19 Fig. Immunofluorescent image of receptor activity modifying protein 1 (RAMP1) in the Vc. S20 Fig. Immunofluorescent image of D2 receptor in the Vc. S21 Fig. Merged image of RAMP1 and D2 receptor in the Vc. S22 Fig. Photomicrograph of CGRP-IR neurons in the trige [file pone.0323810.s001.zip › supplementary/Fig S61.tif]

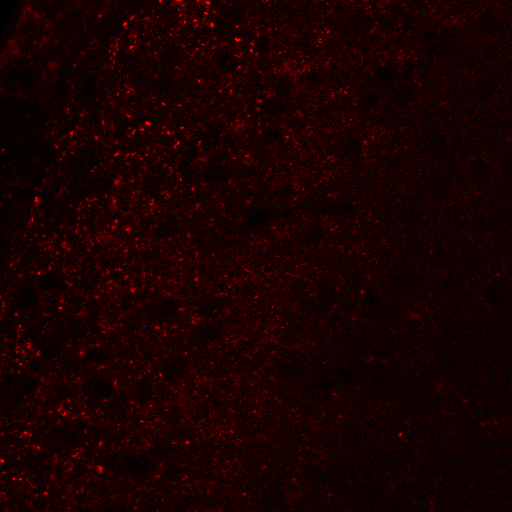

Supplement: S1 Data — S1 Fig. Photomicrograph of phosphorylated extracellular signal-regulated kinase (pERK)-immunoreactive (-IR) neurons in the trigeminal spinal subnucleus caudalis (Vc) of a sham rat. S2 Fig. Photomicrograph of pERK-IR neurons in the Vc of a chronic constriction injury of the infraorbital nerve (ION-CCI) rat. S3 Fig. Photomicrograph of pERK-IR neurons in the Vc of an ION-CCI rat receiving a vehicle. S4 Fig. Photomicrograph of pERK-IR neurons in the Vc of an ION-CCI rat receiving calcitonin gene-related peptide (CGRP). S5 Fig. Photomicrograph of pERK-IR neurons in the Vc of an ION-CCI rat receiving control immunogloublin G (IgG). S6 Fig. Photomicrograph of pERK-IR neurons in the Vc of an ION-CCI rat receiving an anti-CGRP antibody. S7 Fig. High magnification photomicrograph of pERK-IR neurons in the Vc of a sham rat. S8 Fig. High magnification photomicrograph of pERK-IR neurons in the Vc of an ION-CCI rat. S9 Fig. High magnification photomicrograph of pERK-IR neurons in the Vc of an ION-CCI rat receiving a vehicle. S10 Fig. High magnification photomicrograph of pERK-IR neurons in the Vc of an ION-CCI rat receiving CGRP. S11 Fig. High magnification photomicrograph of pERK-IR neurons in the Vc of an ION-CCI rat receiving control IgG. S12 Fig. High magnification photomicrograph of pERK-IR neurons in the Vc of an ION-CCI rat receiving an anti-CGRP antibody. S13 Fig. Immunofluorescent image of pERK in the Vc. S14 Fig. Immunofluorescent image of neuronal nuclei (NeuN) in the Vc. S15 Fig. Merged image of pERK and NeuN in the Vc. S16 Fig. Immunofluorescent image of pERK in the Vc. S17 Fig. Immunofluorescent image of dopamineD2 receptor (D2 receptor) in the Vc. S18 Fig. Merged image of pERK and D2 receptor in the Vc. S19 Fig. Immunofluorescent image of receptor activity modifying protein 1 (RAMP1) in the Vc. S20 Fig. Immunofluorescent image of D2 receptor in the Vc. S21 Fig. Merged image of RAMP1 and D2 receptor in the Vc. S22 Fig. Photomicrograph of CGRP-IR neurons in the trige [file pone.0323810.s001.zip › supplementary/Fig S62.tif]

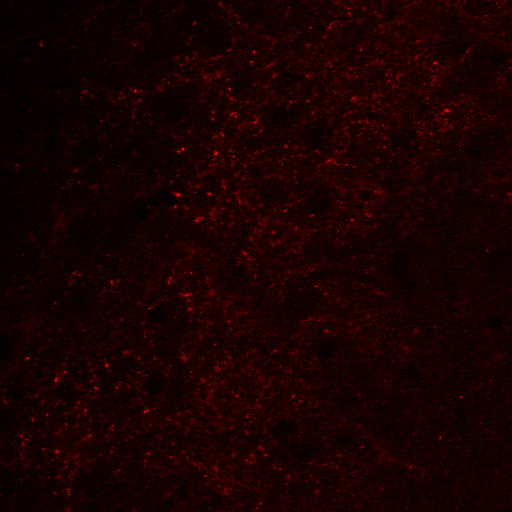

Supplement: S1 Data — S1 Fig. Photomicrograph of phosphorylated extracellular signal-regulated kinase (pERK)-immunoreactive (-IR) neurons in the trigeminal spinal subnucleus caudalis (Vc) of a sham rat. S2 Fig. Photomicrograph of pERK-IR neurons in the Vc of a chronic constriction injury of the infraorbital nerve (ION-CCI) rat. S3 Fig. Photomicrograph of pERK-IR neurons in the Vc of an ION-CCI rat receiving a vehicle. S4 Fig. Photomicrograph of pERK-IR neurons in the Vc of an ION-CCI rat receiving calcitonin gene-related peptide (CGRP). S5 Fig. Photomicrograph of pERK-IR neurons in the Vc of an ION-CCI rat receiving control immunogloublin G (IgG). S6 Fig. Photomicrograph of pERK-IR neurons in the Vc of an ION-CCI rat receiving an anti-CGRP antibody. S7 Fig. High magnification photomicrograph of pERK-IR neurons in the Vc of a sham rat. S8 Fig. High magnification photomicrograph of pERK-IR neurons in the Vc of an ION-CCI rat. S9 Fig. High magnification photomicrograph of pERK-IR neurons in the Vc of an ION-CCI rat receiving a vehicle. S10 Fig. High magnification photomicrograph of pERK-IR neurons in the Vc of an ION-CCI rat receiving CGRP. S11 Fig. High magnification photomicrograph of pERK-IR neurons in the Vc of an ION-CCI rat receiving control IgG. S12 Fig. High magnification photomicrograph of pERK-IR neurons in the Vc of an ION-CCI rat receiving an anti-CGRP antibody. S13 Fig. Immunofluorescent image of pERK in the Vc. S14 Fig. Immunofluorescent image of neuronal nuclei (NeuN) in the Vc. S15 Fig. Merged image of pERK and NeuN in the Vc. S16 Fig. Immunofluorescent image of pERK in the Vc. S17 Fig. Immunofluorescent image of dopamineD2 receptor (D2 receptor) in the Vc. S18 Fig. Merged image of pERK and D2 receptor in the Vc. S19 Fig. Immunofluorescent image of receptor activity modifying protein 1 (RAMP1) in the Vc. S20 Fig. Immunofluorescent image of D2 receptor in the Vc. S21 Fig. Merged image of RAMP1 and D2 receptor in the Vc. S22 Fig. Photomicrograph of CGRP-IR neurons in the trige [file pone.0323810.s001.zip › supplementary/Fig S63.tif]

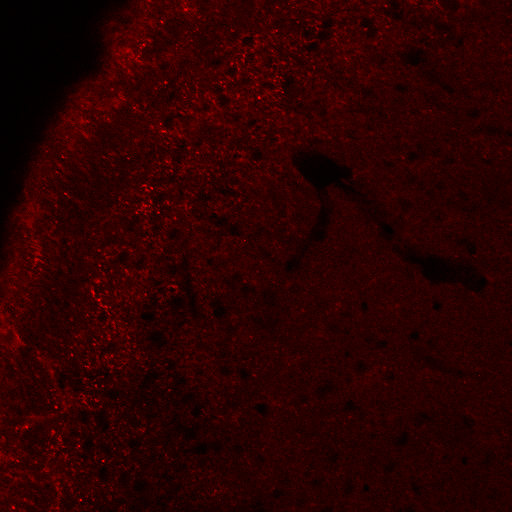

Supplement: S1 Data — S1 Fig. Photomicrograph of phosphorylated extracellular signal-regulated kinase (pERK)-immunoreactive (-IR) neurons in the trigeminal spinal subnucleus caudalis (Vc) of a sham rat. S2 Fig. Photomicrograph of pERK-IR neurons in the Vc of a chronic constriction injury of the infraorbital nerve (ION-CCI) rat. S3 Fig. Photomicrograph of pERK-IR neurons in the Vc of an ION-CCI rat receiving a vehicle. S4 Fig. Photomicrograph of pERK-IR neurons in the Vc of an ION-CCI rat receiving calcitonin gene-related peptide (CGRP). S5 Fig. Photomicrograph of pERK-IR neurons in the Vc of an ION-CCI rat receiving control immunogloublin G (IgG). S6 Fig. Photomicrograph of pERK-IR neurons in the Vc of an ION-CCI rat receiving an anti-CGRP antibody. S7 Fig. High magnification photomicrograph of pERK-IR neurons in the Vc of a sham rat. S8 Fig. High magnification photomicrograph of pERK-IR neurons in the Vc of an ION-CCI rat. S9 Fig. High magnification photomicrograph of pERK-IR neurons in the Vc of an ION-CCI rat receiving a vehicle. S10 Fig. High magnification photomicrograph of pERK-IR neurons in the Vc of an ION-CCI rat receiving CGRP. S11 Fig. High magnification photomicrograph of pERK-IR neurons in the Vc of an ION-CCI rat receiving control IgG. S12 Fig. High magnification photomicrograph of pERK-IR neurons in the Vc of an ION-CCI rat receiving an anti-CGRP antibody. S13 Fig. Immunofluorescent image of pERK in the Vc. S14 Fig. Immunofluorescent image of neuronal nuclei (NeuN) in the Vc. S15 Fig. Merged image of pERK and NeuN in the Vc. S16 Fig. Immunofluorescent image of pERK in the Vc. S17 Fig. Immunofluorescent image of dopamineD2 receptor (D2 receptor) in the Vc. S18 Fig. Merged image of pERK and D2 receptor in the Vc. S19 Fig. Immunofluorescent image of receptor activity modifying protein 1 (RAMP1) in the Vc. S20 Fig. Immunofluorescent image of D2 receptor in the Vc. S21 Fig. Merged image of RAMP1 and D2 receptor in the Vc. S22 Fig. Photomicrograph of CGRP-IR neurons in the trige [file pone.0323810.s001.zip › supplementary/Fig S64.tif]

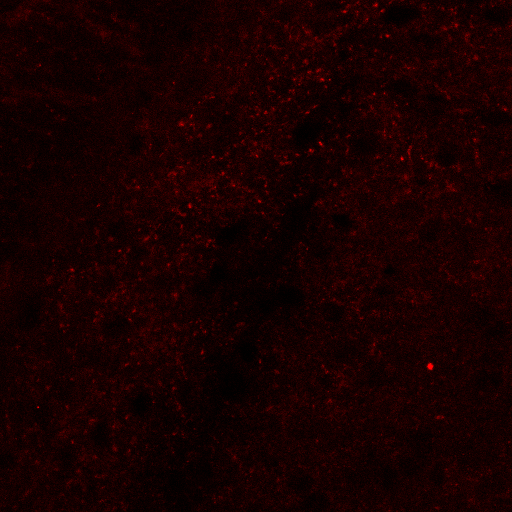

Supplement: S1 Data — S1 Fig. Photomicrograph of phosphorylated extracellular signal-regulated kinase (pERK)-immunoreactive (-IR) neurons in the trigeminal spinal subnucleus caudalis (Vc) of a sham rat. S2 Fig. Photomicrograph of pERK-IR neurons in the Vc of a chronic constriction injury of the infraorbital nerve (ION-CCI) rat. S3 Fig. Photomicrograph of pERK-IR neurons in the Vc of an ION-CCI rat receiving a vehicle. S4 Fig. Photomicrograph of pERK-IR neurons in the Vc of an ION-CCI rat receiving calcitonin gene-related peptide (CGRP). S5 Fig. Photomicrograph of pERK-IR neurons in the Vc of an ION-CCI rat receiving control immunogloublin G (IgG). S6 Fig. Photomicrograph of pERK-IR neurons in the Vc of an ION-CCI rat receiving an anti-CGRP antibody. S7 Fig. High magnification photomicrograph of pERK-IR neurons in the Vc of a sham rat. S8 Fig. High magnification photomicrograph of pERK-IR neurons in the Vc of an ION-CCI rat. S9 Fig. High magnification photomicrograph of pERK-IR neurons in the Vc of an ION-CCI rat receiving a vehicle. S10 Fig. High magnification photomicrograph of pERK-IR neurons in the Vc of an ION-CCI rat receiving CGRP. S11 Fig. High magnification photomicrograph of pERK-IR neurons in the Vc of an ION-CCI rat receiving control IgG. S12 Fig. High magnification photomicrograph of pERK-IR neurons in the Vc of an ION-CCI rat receiving an anti-CGRP antibody. S13 Fig. Immunofluorescent image of pERK in the Vc. S14 Fig. Immunofluorescent image of neuronal nuclei (NeuN) in the Vc. S15 Fig. Merged image of pERK and NeuN in the Vc. S16 Fig. Immunofluorescent image of pERK in the Vc. S17 Fig. Immunofluorescent image of dopamineD2 receptor (D2 receptor) in the Vc. S18 Fig. Merged image of pERK and D2 receptor in the Vc. S19 Fig. Immunofluorescent image of receptor activity modifying protein 1 (RAMP1) in the Vc. S20 Fig. Immunofluorescent image of D2 receptor in the Vc. S21 Fig. Merged image of RAMP1 and D2 receptor in the Vc. S22 Fig. Photomicrograph of CGRP-IR neurons in the trige [file pone.0323810.s001.zip › supplementary/Fig S65.tif]

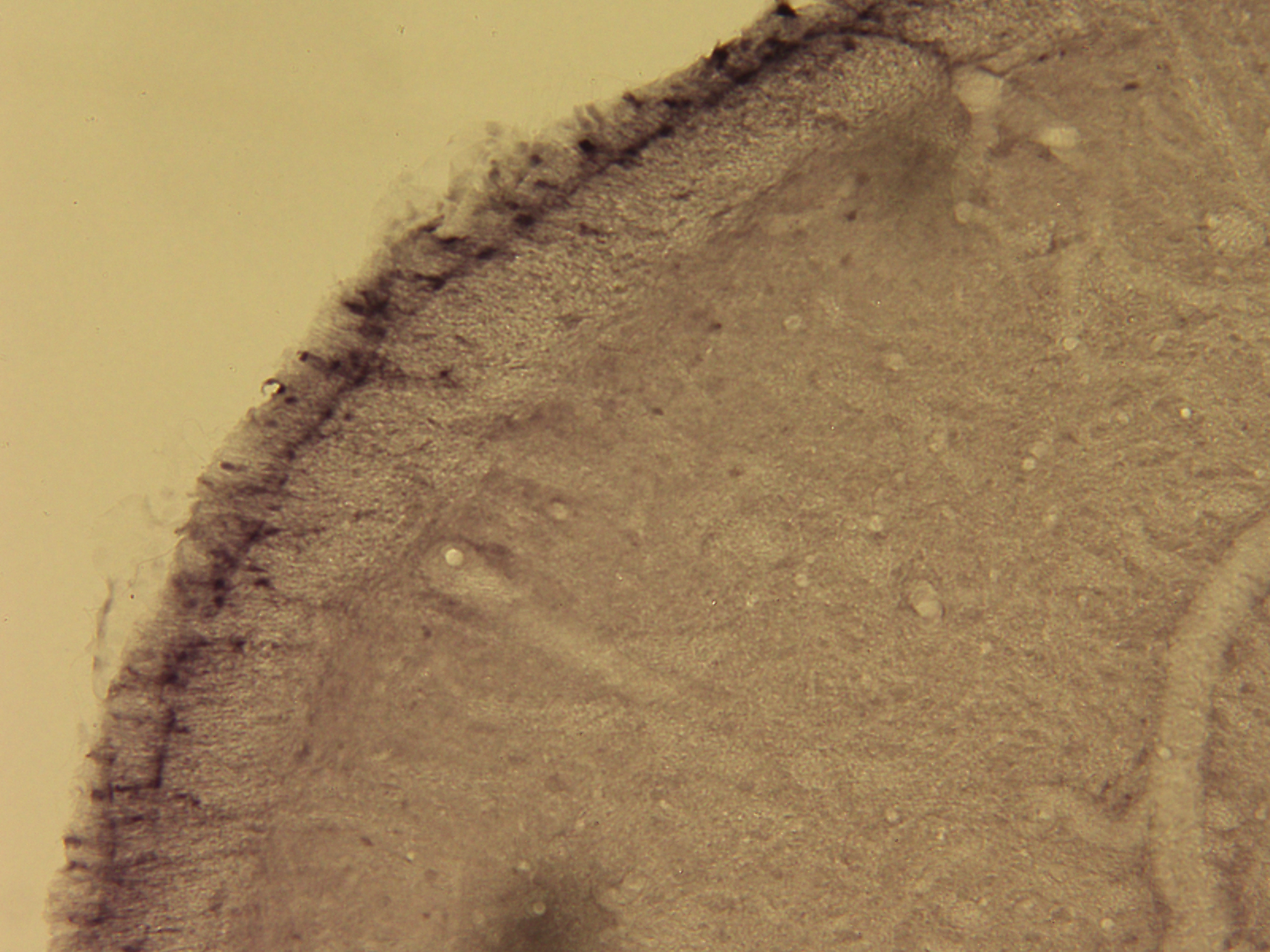

Supplement: S1 Data — S1 Fig. Photomicrograph of phosphorylated extracellular signal-regulated kinase (pERK)-immunoreactive (-IR) neurons in the trigeminal spinal subnucleus caudalis (Vc) of a sham rat. S2 Fig. Photomicrograph of pERK-IR neurons in the Vc of a chronic constriction injury of the infraorbital nerve (ION-CCI) rat. S3 Fig. Photomicrograph of pERK-IR neurons in the Vc of an ION-CCI rat receiving a vehicle. S4 Fig. Photomicrograph of pERK-IR neurons in the Vc of an ION-CCI rat receiving calcitonin gene-related peptide (CGRP). S5 Fig. Photomicrograph of pERK-IR neurons in the Vc of an ION-CCI rat receiving control immunogloublin G (IgG). S6 Fig. Photomicrograph of pERK-IR neurons in the Vc of an ION-CCI rat receiving an anti-CGRP antibody. S7 Fig. High magnification photomicrograph of pERK-IR neurons in the Vc of a sham rat. S8 Fig. High magnification photomicrograph of pERK-IR neurons in the Vc of an ION-CCI rat. S9 Fig. High magnification photomicrograph of pERK-IR neurons in the Vc of an ION-CCI rat receiving a vehicle. S10 Fig. High magnification photomicrograph of pERK-IR neurons in the Vc of an ION-CCI rat receiving CGRP. S11 Fig. High magnification photomicrograph of pERK-IR neurons in the Vc of an ION-CCI rat receiving control IgG. S12 Fig. High magnification photomicrograph of pERK-IR neurons in the Vc of an ION-CCI rat receiving an anti-CGRP antibody. S13 Fig. Immunofluorescent image of pERK in the Vc. S14 Fig. Immunofluorescent image of neuronal nuclei (NeuN) in the Vc. S15 Fig. Merged image of pERK and NeuN in the Vc. S16 Fig. Immunofluorescent image of pERK in the Vc. S17 Fig. Immunofluorescent image of dopamineD2 receptor (D2 receptor) in the Vc. S18 Fig. Merged image of pERK and D2 receptor in the Vc. S19 Fig. Immunofluorescent image of receptor activity modifying protein 1 (RAMP1) in the Vc. S20 Fig. Immunofluorescent image of D2 receptor in the Vc. S21 Fig. Merged image of RAMP1 and D2 receptor in the Vc. S22 Fig. Photomicrograph of CGRP-IR neurons in the trige [file pone.0323810.s001.zip › supplementary/Fig S7.TIF]

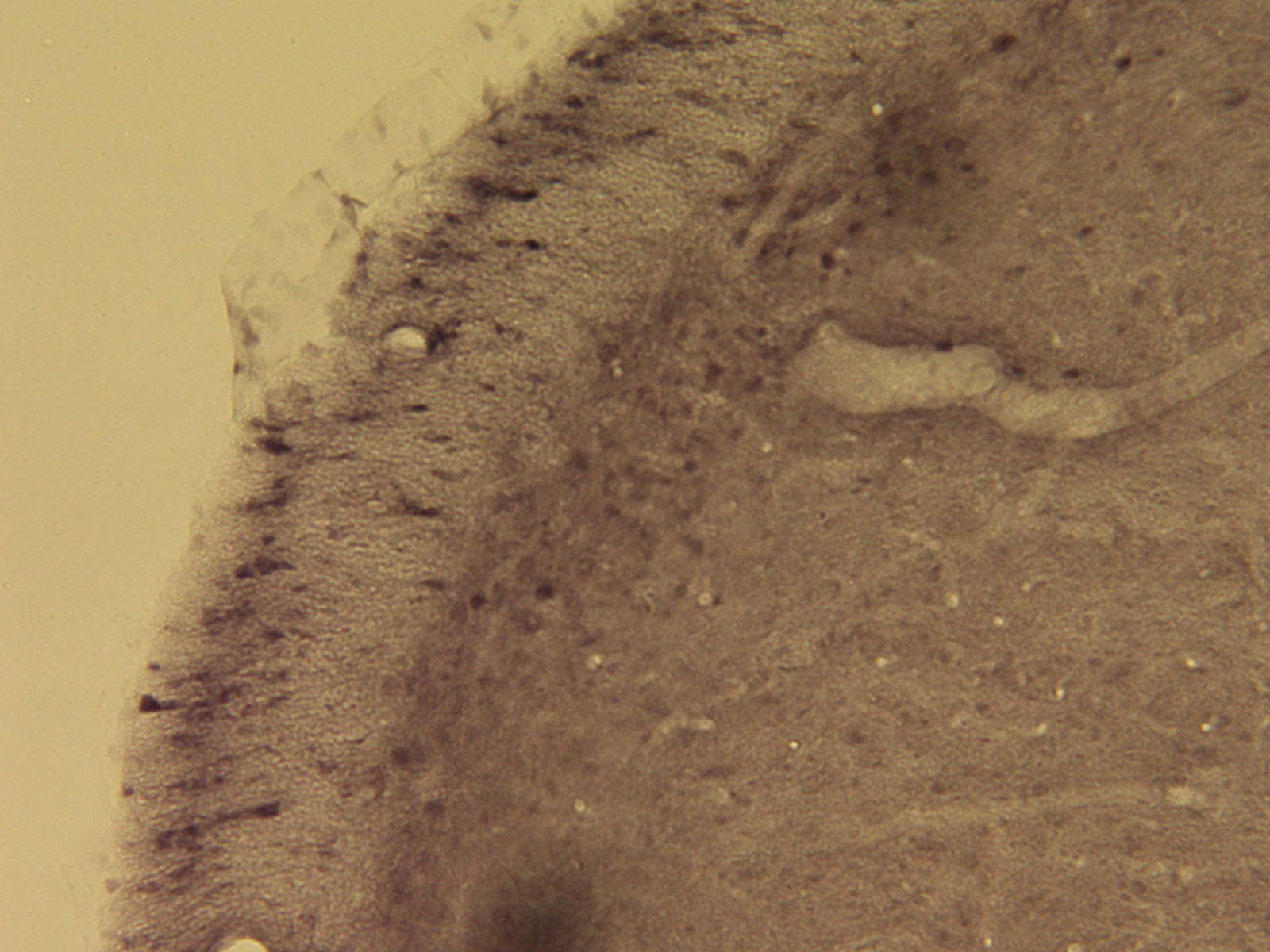

Supplement: S1 Data — S1 Fig. Photomicrograph of phosphorylated extracellular signal-regulated kinase (pERK)-immunoreactive (-IR) neurons in the trigeminal spinal subnucleus caudalis (Vc) of a sham rat. S2 Fig. Photomicrograph of pERK-IR neurons in the Vc of a chronic constriction injury of the infraorbital nerve (ION-CCI) rat. S3 Fig. Photomicrograph of pERK-IR neurons in the Vc of an ION-CCI rat receiving a vehicle. S4 Fig. Photomicrograph of pERK-IR neurons in the Vc of an ION-CCI rat receiving calcitonin gene-related peptide (CGRP). S5 Fig. Photomicrograph of pERK-IR neurons in the Vc of an ION-CCI rat receiving control immunogloublin G (IgG). S6 Fig. Photomicrograph of pERK-IR neurons in the Vc of an ION-CCI rat receiving an anti-CGRP antibody. S7 Fig. High magnification photomicrograph of pERK-IR neurons in the Vc of a sham rat. S8 Fig. High magnification photomicrograph of pERK-IR neurons in the Vc of an ION-CCI rat. S9 Fig. High magnification photomicrograph of pERK-IR neurons in the Vc of an ION-CCI rat receiving a vehicle. S10 Fig. High magnification photomicrograph of pERK-IR neurons in the Vc of an ION-CCI rat receiving CGRP. S11 Fig. High magnification photomicrograph of pERK-IR neurons in the Vc of an ION-CCI rat receiving control IgG. S12 Fig. High magnification photomicrograph of pERK-IR neurons in the Vc of an ION-CCI rat receiving an anti-CGRP antibody. S13 Fig. Immunofluorescent image of pERK in the Vc. S14 Fig. Immunofluorescent image of neuronal nuclei (NeuN) in the Vc. S15 Fig. Merged image of pERK and NeuN in the Vc. S16 Fig. Immunofluorescent image of pERK in the Vc. S17 Fig. Immunofluorescent image of dopamineD2 receptor (D2 receptor) in the Vc. S18 Fig. Merged image of pERK and D2 receptor in the Vc. S19 Fig. Immunofluorescent image of receptor activity modifying protein 1 (RAMP1) in the Vc. S20 Fig. Immunofluorescent image of D2 receptor in the Vc. S21 Fig. Merged image of RAMP1 and D2 receptor in the Vc. S22 Fig. Photomicrograph of CGRP-IR neurons in the trige [file pone.0323810.s001.zip › supplementary/Fig S8.TIF]

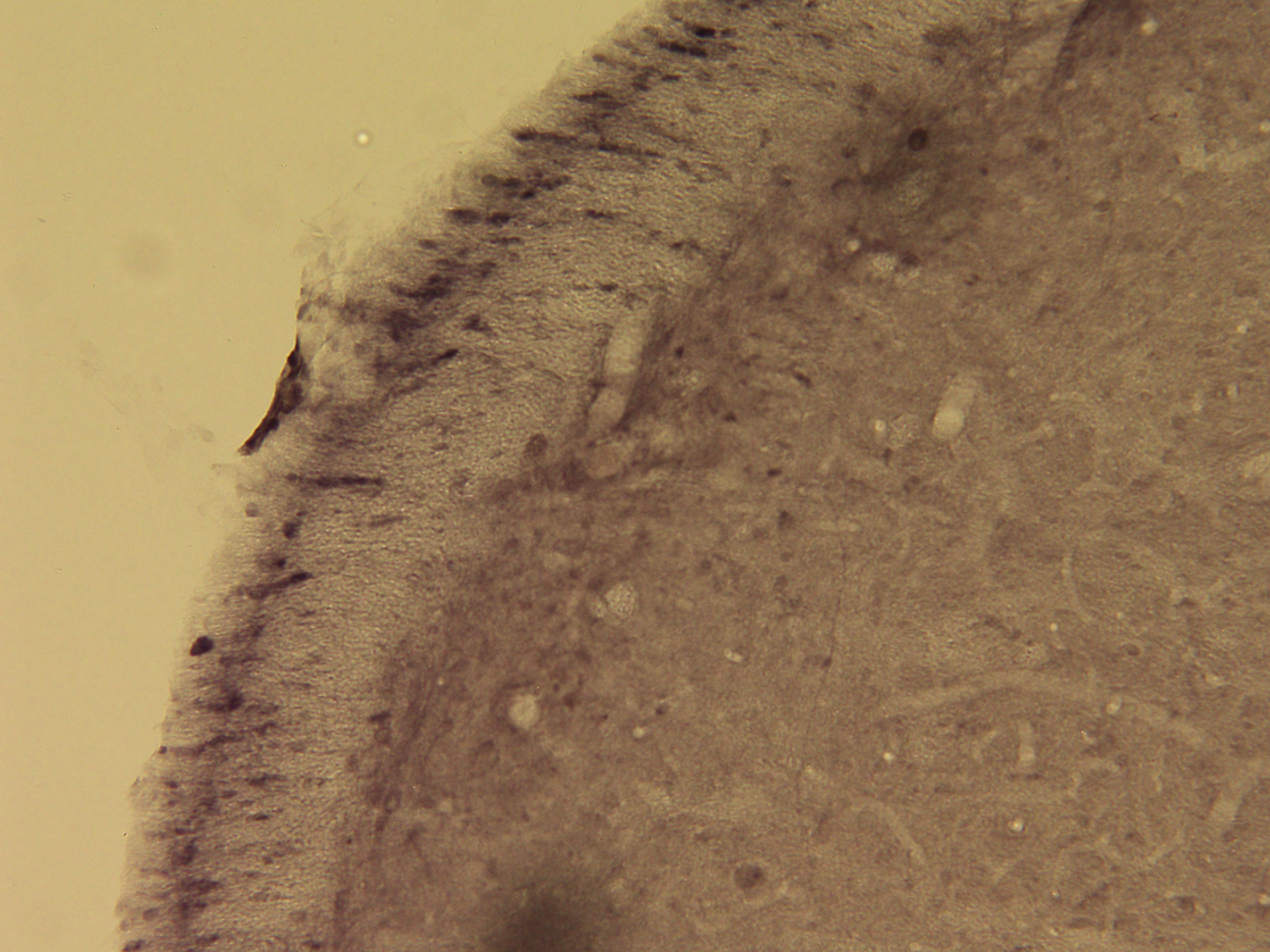

Supplement: S1 Data — S1 Fig. Photomicrograph of phosphorylated extracellular signal-regulated kinase (pERK)-immunoreactive (-IR) neurons in the trigeminal spinal subnucleus caudalis (Vc) of a sham rat. S2 Fig. Photomicrograph of pERK-IR neurons in the Vc of a chronic constriction injury of the infraorbital nerve (ION-CCI) rat. S3 Fig. Photomicrograph of pERK-IR neurons in the Vc of an ION-CCI rat receiving a vehicle. S4 Fig. Photomicrograph of pERK-IR neurons in the Vc of an ION-CCI rat receiving calcitonin gene-related peptide (CGRP). S5 Fig. Photomicrograph of pERK-IR neurons in the Vc of an ION-CCI rat receiving control immunogloublin G (IgG). S6 Fig. Photomicrograph of pERK-IR neurons in the Vc of an ION-CCI rat receiving an anti-CGRP antibody. S7 Fig. High magnification photomicrograph of pERK-IR neurons in the Vc of a sham rat. S8 Fig. High magnification photomicrograph of pERK-IR neurons in the Vc of an ION-CCI rat. S9 Fig. High magnification photomicrograph of pERK-IR neurons in the Vc of an ION-CCI rat receiving a vehicle. S10 Fig. High magnification photomicrograph of pERK-IR neurons in the Vc of an ION-CCI rat receiving CGRP. S11 Fig. High magnification photomicrograph of pERK-IR neurons in the Vc of an ION-CCI rat receiving control IgG. S12 Fig. High magnification photomicrograph of pERK-IR neurons in the Vc of an ION-CCI rat receiving an anti-CGRP antibody. S13 Fig. Immunofluorescent image of pERK in the Vc. S14 Fig. Immunofluorescent image of neuronal nuclei (NeuN) in the Vc. S15 Fig. Merged image of pERK and NeuN in the Vc. S16 Fig. Immunofluorescent image of pERK in the Vc. S17 Fig. Immunofluorescent image of dopamineD2 receptor (D2 receptor) in the Vc. S18 Fig. Merged image of pERK and D2 receptor in the Vc. S19 Fig. Immunofluorescent image of receptor activity modifying protein 1 (RAMP1) in the Vc. S20 Fig. Immunofluorescent image of D2 receptor in the Vc. S21 Fig. Merged image of RAMP1 and D2 receptor in the Vc. S22 Fig. Photomicrograph of CGRP-IR neurons in the trige [file pone.0323810.s001.zip › supplementary/Fig S9.TIF]
