# Supplementary material for: Suppression of mechanical hypersensitivity and change in the expression of the dopamine D2 receptor by administration of anti-CGRP antibody into the trigeminal ganglion in trigeminal neuropathic pain model rats
Source: PLoS One. 2025 May 14;20(5):e0323810. doi: 10.1371/journal.pone.0323810 (PMC12077688; doi:10.1371/journal.pone.0323810)
Supplement: Attachment — Submitted filename: Response to reviewers.docx [file pone.0323810.s004.docx]

Thank you for reviewing our manuscript. We would like to provide our responses to the reviewer's comments.

**Reviewer #1**: The authors Maegawa et al. have reported that the interaction of CGRP and D2 receptor in the trigeminal ganglion neurons is involved in orofacial neuropathic pain development using infraorbital nerve ligation (ION-CCI) model rats. The following are comments on this paper:

Major

1. The authors want to mention that the interaction between pERK-IR and D2 receptor expression in Vc neurons is involved in the orofacial neuropathic pain mechanism. To clarify this question, double immunostaining of CGRP and D2 receptors should be conducted in each group of animals.

**Response**

Thank you for reviewing our manuscript. The CGRP receptor consists of receptor activity modifying protein 1 (RAMP1) and calcitonin receptor-like receptor (CLR). To show the CGRP receptor by immunostaining, double staining of RAMP1 and CLR is required. However, although anti-RAMP1 antibodies are commercially available, we could not find any commercially available anti-CLR antibodies. There are reports of self-preparing anti-CLR antibodies, but we have no experience in producing antibodies ourselves and thought it would be difficult to prepare anti-CLR antibodies. Therefore, we performed double staining of RAMP1 and D2 receptors, and obtained images suggesting colocalization of RAMP1 and D2 receptor in Vc (Fig 3F). The colocalization was observed in all experimental groups. We are aware that RAMP1 immunoreactivity alone does not completely show the CGRP receptor, but we adopted this method as an experimental method that we could use. This has also been noted as a limitation in the discussion (from page13 line 231 to page 14 line 244, from page 20 line 346 to line 348, from page 21 line 364 to line 366, and from page 33 line 574 to line 579 in manuscript; from page 14 line 248 to page 15 line 264, from page 21 line 369 to line 371, from page 22 line 390 to line 392, and from page 35 line 625 to page36 line 630 in revised manuscript with track changes).

2. Furthermore, did D2 receptor immunoreactivity express in neurons or non-neuronal cells? The authors need to show the double immunohistochemistry of pERK and NeuN, as well as the D2 receptor and NeuN.

**Response**

We performed double staining of Vc sections for pERK and NeuN, and D2 receptor and NeuN. Colocalization of pERK and NeuN were observed, and colocalization of D2 receptor and NeuN were observed (Fig 3D, E). These colocalizations were observed in all experimental groups. We wrote the above (from page 13 line 231 to page 14 line 244, from page 20 line 342 to line343, and from page 21 line 360 to line 362, from page 26 line 463 to line 465, from page 27 line 476 to line 477 in manuscript; from page 14 line 248 to page 15 line 264, from page 21 line 364 to line 366, and from page 22 line 385 to line 387, from page 29 line 504 to line 506, from page 29 line 517 to 519 in revised manuscript with track changes). Therefore, we considered that pERK and D2 receptor are expressed in neurons.

3. The authors need to test if CGRP injection into TG causes mechanical hypersensitivity in naïve rats.

**Response**

CGRP was administered to the trigeminal ganglion of naïve rats, and the threshold for the escape response to mechanical stimulation was measured. A decrease in the threshold was observed one day after the final administration (Fig 8). No significant change in the threshold was observed in the vehicle-administered group. We wrote the above (from page 8 line 142 to line 143, from page 9 line 150 to line 153, and from page 27 line 483 to page 28 line 497 in manuscript; from page 9 line 153 to line 154, page 9 line 161 to page 10 line 164, and from page 30 line 525 to page 31 line 540 in revised manuscript with track changes).

4. CGRP may be released from central terminals of CGRP-IR cells in Vc and act on D2 receptor-IR cells. If this is so, the authors need to show the interaction between CGRP-IR cells and D2 receptor-expressing cells.

**Response**

Double staining for CGRP and D2 receptor was performed in Vc. Immunofluorescent images showed close proximity of CGRP immunoreactivity and D2 receptor immunoreactivity (Fig 6C). We wrote the above (from page 13 line 231 to page 14 line 244, from page 25 line 438 to line 441, and from page 25 line 447 to page 26 line 449 in manuscript; from page 14 line 248 to page 15 line 264, from page 27 line 478 to line 481, and page 28 line 487 to line 489 in revised manuscript with track changes).

5. Does intrathecal administration of CGRP or CGRP Ab cause D2 receptor expression or drown regulation in Vc in ION-CCI rats? The authors need to conduct this experiment.

**Response**

We performed an intracisternal administration of CGRP and anti-CGRP antibody to ION-CCI rats. A decrease in D2 receptor immunoreactivity was found in ION-CCI rats receiving anti-CGRP antibodies (Fig 9). We wrote the above (from page 9 line 153 to line 157, from page 11 line 186 to page 12 line 201, and from page28 line 499 to page 29 line 516 in manuscript; from page 10 line 164 to line 168, from page 12 line 199 to line 216, and page 31 line 542 to page 32 line 561 in revised manuscript with track changes).

6. Since the authors mentioned a lot about the involvement of satellite glial cells in orofacial neuropathic pain in the discussion section, the authors need to show the immunohistochemistry data of satellite cell activation in TG following ION-CCI.

**Response**

Immunostaining for GFAP was performed on trigeminal ganglion sections. GFAP-immunoreactive cells were observed around TG neurons, which was consistent with the presence of activated satellite glial cells around TG neurons (Fig5A). GFAP immunoreactivity was quantified by calculating the ratio of TG neurons encircled with GFAP-immunoreactive cells. Infraorbital nerve ligation increased the ratio of TG neurons encircled with GFAP immunoreactive cells (Fig 5B). No further increase was observed after CGRP administration. Administration of anti-CGRP antibody decreased the ratio of TG neurons encircled with GFAP immunoreactive cells (Fig 5B). We wrote the above (from page 2 line 31 to line33, from page 13 line 231 to page 14 line 244, and from page 23 line 408 to page 24 line 428 in manuscript; from page 2 line 32 to line 34, from page 14 line 248 to page 15 line 264, and from page 25 line 447 to page 27 line 468 in revised manuscript with track changes).

Minor

1. The authors addressed the head-withdrawal threshold (HWT) in each group of animals, sham, ION-CCI, ION-CCI+vehile injection into trigeminal ganglion (TG), ION-CCI+CGRP injection into TG, ION-CCI+IgG injection into TG and ION-CCI+CGRP antibody injection into TG. It’s better to show the time course change in HWT.

**Response**

When measuring the head-withdrawal threshold after drug administration, an increase in the threshold was observed in ION-CCI rats that received anti-CGRP antibody. Therefore, we investigated the change in threshold over time in this group. Compared to before administration of anti-CGRP antibody, an increase in the threshold was observed 1 and 2 days after the end of administration (Fig 2B). We wrote the above (from page 10 line 177 to line 178, from page18 line 304 to line 308, from page 18 line 321 to page 19 line 325 in manuscript; from page 11 line 189 to line 191, from page 19 line 325 to line 328, from page 20 line 342 to line 346 in revised manuscript with track changes).

2. High-magnification photomicrographs should be addressed in Fig. 1 B and Fig. 4A.

**Response**

We presented high-magnification photomicrographs of Fig1B and 4A. Fig 1B is now Fig 3B. Fig 4A is now Fig 7B.

3. Some large-sized TG cells look CGRP-IR. It is necessary to analyze the cell size of CGRP-IR cells in ION-CCI tars.

**Response**

The size of CGRP-immunoreactive cells was analyzed (Fig 4C). Following previous literature, the size of CGRP-immunoreactive cells was divided into small, medium, and large according to area, and the ratio of the number of CGRP-immunoreactive cells of each size to the total number of CGRP-immunoreactive cells was calculated. Differences in the ratio were observed in the ION-CCI group, ION-CCI+vehi group, ION-CCI+CGRP group, and ION-CCI+ctrl IgG group compared to the sham group. In addition, differences were observed in the ratio of the ION-CCI+CGRP group and ION-CCI+ctrl IgG group compared to the ION-CCI+CGRP Ab group. We wrote the above (from page 15 line 258 to line 260, from page 17 line 289 to line 291, from page 22 line 379 to line 385, from page 23 line 395 to line 400 in manuscript; from page 16 line 278 to line 281, from page 18 line 309 to line 311, from page 24 line 417 to line 423, from page 25 line 433 to line 439 in revised manuscript with track changes).

4. The photomicrographs in Fig.4A are pretty low quality. Need to show higher quality photomicrographs in Fig. 4A.

**Response**

The photomicrograph in Fig 4A has been updated to a higher quality version (Fig 7A).

5. The discussion section is awkwardly addressed in the current paper. The discussion section should be totally rewritten.

**Response**

We have revised the discussion. We have deleted content that is not relevant to the results of the present study. We have also included the results of additional experiments based on the reviewers’ comments. As a result of these revisions, we believe that we have been able to strengthen our discussion. The corrections are follows: from page 30 line 525 to page 31 line 542, from page32 line 560 to line 563, from page 32 line 572 to page 33 line 581, from page 33 line 583 to line 591, from page 34 line 604 to line 608 in manuscript; from page 32 line 571 to page 33 line 589, from page 34 line 609 to page 35 line 613, from page 35 line 623 to page 36 line 632, from page 36 line 635 to line 643, from page 38 line 680 to page 39 line 685 in revised manuscript with track changes.

**Reviewer #2**: The authors found an interaction between CGRP and D2R in the Vc after IONI. TG administration of anti-CGRP antibody decreased CGRP and D2R expression in the Vc. However, the authors need to address the following issues to strengthen the manuscript.

The authors noted that the fluorescence intensity correlates with the amount of CGRP and D2R, using Ref. It is quite difficult to conclude from fluorescence alone without measuring protein levels by Western blot analysis. The authors must show Western blot data.

**Response**

Thank you for reviewing our manuscript. We agree with the reviewer's comment. However, we performed Western blot experiments, but unfortunately, we were unable to obtain reproducible data. We have described this in the discussion section as a limitation (from page 32 line 561 to line 563 in manuscript, from page 36 line 629 to line 630 revised manuscript with track changes).

Pentobarbital cannot be used for anesthesia due to the welfare of the animals. Use the appropriate anesthetic.

**Response**

We agree with reviewer’s comment. We did not use pentobarbital. We used a saline solution containing midazolam, medetomidine, and butorphanol as anesthetic in all experiments in the present study. We have mentioned the above (from page 6 line 95 to line 99 in manuscript, from page 6 line 104 to line 108 in revised manuscript with track changes).

Which cells in Vc express D2R? If CGRP projected to the Vc regulates D2R expression, then D2R and pERK are likely expressed in the same cells. Double staining of D2R and pERK should be done. Describe the cellular mechanisms by which CGRP increases D2R expression. If CGRP is altering D2R expression through changes in activity in the upper brain, there is no proof of this, and further experiments should be done.

**Response**

We performed double staining for D2 receptors and pERK. As a result, we found colocalization of D2 receptor and pERK (Fig. 3D). We wrote the above (from page 13 line 231 to page 14 line 244, from page 20 line 344 to line 346, from page 21 line 362 to line 364 in manuscript; from page 14 line 248 to page 15 line 264, from page 21 line 366 to line 369, from page 22 line 385 to line 387 in revised manuscript with track changes).

Colocalization of RAMP1 and D2 receptors was observed (Fig. 3F), suggesting colocalization of CGRP receptors and D2 receptors. There have also been reports that CGRP receptors exist in Vc neurons (36). We hypothesized that after CGRP binds to the CGRP receptor, an intracellular signaling mechanism is activated, leading to increased protein expression, one of which is the D2 receptor. However, another report showed that Vc neurons did not express CGRP receptors [37]. NMDA and AMPA receptors are present in the second order neurons of the Vc [38]. Sustained excitation of primary nociceptive neurons, such as that caused by peripheral nerve injury or chronic inflammation, increases the synthesis and release of neurotransmitters such as SP and CGRP from their central terminals, which enhances the excitability of AMPA and NMDA receptors in second order neurons [39]. Increased excitability of NMDA receptors leads to an influx of calcium ions into the cell, which can activate protein kinase C and affect transcription [10]. We wrote the above (from page 32 line 572 to page 33 line 591 in manuscript, from page 35 line 623 to page 36 line 643 in revised manuscript with track changes).

This study did not examine activity in the upper brain. Therefore, the contents of page19, line 341-page 20, line 351 in the first submitted manuscript were deleted because they were not considered appropriate as a discussion of the results of this study (from page 37 line 654 to page 38 line 670).

**Reviewer #**3: PONE-D-24-44553

In the manuscript entitled “Suppression of mechanical hypersensitivity and change in the expression of the dopamine D2 receptor by administration of anti-CGRP antibody into the trigeminal ganglion in trigeminal neuropathic pain model rats” the Authors aimed to investigate the relationship between CGRP and the dopaminergic nervous system in neuropathic pain following anti-CGRP Ab treatment in the trigeminal ganglion.

The Authors should clearly define in the introduction section the differences from the previous study (DOI: 10.1016/j.bbrc.2022.05.015) in which they already investigated the relationship between the CGRP and dopaminergic pathway. Indeed it should be highlighted the novelty of the present study together with the aim of the study. The Authors stated: “The current study aims to elucidate the relationship between CGRP and the dopaminergic nervous system in neuropathic pain further.”; to achieve this purpose they only looked at the Dopamine D2 receptor immunoreactivity in the Vc following an anti-CGRP Ab treatment, which was already investigated in a previous paper from the same Authors (doi: 10.1016/j.bbrc.2022.05.015). The only difference was the site of anti-CGRP Ab administration (cerebroventricular vs TG), but the result is the same: dopamine D2 receptor immunostaining in the Vc decreased after treatment with an anti-CGRP antibody.

In the present form this study does not seem to provide additional information to the field.

**Response**

Thank you for reviewing our manuscript. In our previous study in which CGRP antibodies were administered intracerebroventricularly, it was thought that anti-CGRP antibodies may bind to CGRP released from primary neurons and inhibit CGRP binding to CGRP receptors expressed in Vc. However, CGRP receptors are also present in other sites. Because anti-CGRP antibody administration was intracerebroventricular, it was difficult to identify the site of action of the CGRP antibodies. In this study, we were able to suppress hypersensitivity to mechanical stimuli by administering CGRP antibodies to the trigeminal ganglion. Some of the results obtained are similar to previous studies. However, we believe that the novelty lies in the fact that we showed that neuropathic pain was suppressed and that changes occurred in D2 receptors by acting on CGRP in the trigeminal ganglion. In other words, we were able to clarify the site involved in the inhibition of neuropathic pain more than in previous studies.

Beside the abovementioned issue I have some comments about this manuscript, which is however well written.

1. To specify in the abstract that were used male rats.

**Response**

In the introduction, we mentioned that male rats were used (page 2 line 3 in manuscript, page 2 line 23 in revised manuscript with track changes).

2. To better explain when the animals were sacrificed, e.g. how many days or hours from the last administration, etc.. A schematic representation would be helpful (at least as supplementary material).

**Response**

The experimental schedule is shown (Fig 1, from page 9 line 145 to line 157 in manuscript, from page 9 line 156 to page 10 line 168 in revised manuscript with track changes).

3. Even if references of previous papers from the same Authors (in which they used the same CGRP antibody) are provided it is not clear to me how did they define the dose of an antibody designed to be used in IHC to be injected in the rats. Are there any toxicological test related to its use in vivo?

**Response**

The antibody concentration was based on the following literature: Bai et al. Neuropsychopharmacology 2016; 41:2882-2892, PMID27312407, DOI10.1038/npp.2016.100

Also, Mikuzuki et al. Eur J Neurosci. 2017; 46: 2190-2202. (ref 3 of the present study) administered the drugs into the trigeminal ganglion at a dose of 0.5µL. Based on these literatures, the dose of anti-CGRP antibody was set at 0.5µg/0.5µL. No obvious abnormalities were observed in the animals after antibody administration.

4. To specify that the IHC staining and analysis were performed ipsilateral to the ION-CCI

**Response**

We described that immunohistochemical staining and analysis were performed on the ipsilateral side of infraorbital nerve ligation (from page 12 line 212 to line 213, from page 15 line 252 to line 252, from page 16 line 270 to line 271 in manuscript; from page 13 line 228 to line 229, from page 16 line 271 to line 272, from page 17 line 290 to line 291 in revised manuscript with track changes).

5. Line 192: “As reported previously, pERK-IR cells were primarily found in the superficial layers of the Vc”, please to add references.

**Response**

We added references (page 14 line 247 in manuscript, from page 15 line 267 to page 16 line 268 in revised manuscript with track changes).

6. To add the sample size calculation. Which was the primary outcome?

**Response**

The sample size was determined based on our previous studies.

The primary outcome was to determine whether administration of anti-CGRP antibody to the trigeminal ganglion of a rat neuropathic pain model induced by infraorbital nerve ligation suppresses hypersensitivity to mechanical stimuli and whether it reduces the increase in D2 receptor immunoreactivity after nerve ligation in Vc, and finally, to indicate that the site of action of anti-CGRP antibodies is the trigeminal ganglion.

7. To add which test was used for testing data normality.

**Response**

We have added that the Shapiro-Wilk test was used to test for normality (page 16 line 280 in manuscript, from page 17 line 300 to line 301 in revised manuscript with track changes).

8. To include in the figure legends the statistical test used and N.

**Response**

We described the test used and N in the figure legends (from page 18 line 312 to line 313, from page 19 line 322 to line 324, from page 20 line 354 to line 356, from page 22 line 389 to line 390, from page 23 line 396 to line 397, from page 24 line 419 to line 420, from page 25 line 445 to line 446, from page 27 line 470 to line 471, from page 28 line 489 to line 493, from page 29 line 509 to line 510 in manuscript; from page 19 line 332 to line 333, from page 20 line 343 to line 345, from page 22 line 378 to line 380, from page 24 line 427 to line 428, from page 25 line 434 to line 435, from page 26 line 458 to line 459, from page 28 line 485 to line 486, from page 29 line 511 to line 513, from page 30 line 531 to line 536, from page 31 line 553 to line 554 in revised manuscript with track changes).

9. Line 305: “Our findings also suggest an involvement of CGRP and CGRP receptor in the TG on trigeminal neuropathic pain”. In which way the data in this study outline an involvement of CGRP receptor? No investigation on them was achieved.

**Response**

We agree with reviewer’s comment. The results of this study do not indicate the involvement of CGRP receptors in the TG. We deleted the statement regarding the involvement of CGRP receptors in TG (page 32 line 570 in revised manuscript with track changes).

10. The discussion is redundant and speculative, since the finding of the present study do not provide further information.

**Response**

We agree with reviewer’s comments. In the discussion, we deleted descriptions that were not relevant to the present study (from page 37 line 654 to page 38 line 670 in manuscript with track changes). In addition, we conducted additional experiments based on comments from other reviewers, and revised discussion including the results of these experiments. We believe that our discussion was strengthened. The corrections are follows: from page 30 line 525 to page 31 line 542, from page32 line 560 to line 563, from page 32 line 572 to page 33 line 581, from page 33 line 583 to line 591, from page 34 line 604 to line 608 in manuscript; from page 32 line 571 to page 33 line 589, from page 34 line 609 to page 35 line 613, from page 35 line 623 to page 36 line 632, from page 36 line 635 to line 643, from page 38 line 680 to page 39 line 685 in revised manuscript with track changes.

**Reviewer #4**: This is a well-designed and presented study investigating the mechanisms of trigeminal neuropathic pain. In particular the role of CGRP and anti-CGRP antibody in trigeminal neuropathic pain model in rats. The appropriate statistical methods were used and the data is presented clearly. The results support the findings.

Minor comment:

Could you please use a better resolution images for the figures? it would make viewing of the results much easier.

**Response**

Thank you for reviewing our manuscript.

We have increased the resolution of the images.
