# Supplementary material for: Suppression of mechanical hypersensitivity and change in the expression of the dopamine D2 receptor by administration of anti-CGRP antibody into the trigeminal ganglion in trigeminal neuropathic pain model rats
Source: PLoS One. 2025 May 14;20(5):e0323810. doi: 10.1371/journal.pone.0323810 (PMC12077688; doi:10.1371/journal.pone.0323810)
Supplement: Attachment — Submitted filename: Reviewer.docx [file pone.0323810.s003.docx]

**Introduction**

1. **Clear Hypothesis and Objectives**:
   - **Issue**: The introduction lacks a clear statement of the hypothesis and objectives.
   - **Suggestion**: Clearly state the hypothesis and objectives at the beginning to help readers understand the purpose and direction of the research.
2. **Redundancy**:
   - **Issue**: There is redundant information regarding the role of CGRP and its receptors.
   - **Suggestion**: Consolidate this information to make the text more concise and focused.

**Methods**

3. **Detailed Descriptions**:
   - **Issue**: Some methodological details are missing or insufficiently described.
     - **Example**: The exact coordinates for the stereotaxic apparatus are not specified.
     - **Example**: The type of dental acrylic used for fixing the cannula is not mentioned.
     - **Example**: The method of drug administration into the TG and the intracisternal space could be described in more detail to ensure replicability.
   - **Suggestion**: Provide these details to ensure that other researchers can replicate the study accurately.
4. **Consistency in Terminology and Units**:
   - **Issue**: Inconsistencies in the use of terminology and units.
     - **Example**: Ensure that all measurements are in the same units (e.g., grams for weight, microliters for volumes).
   - **Suggestion**: Maintain consistency in terminology and units throughout the section.
5. **Ethical Considerations**:
   - **Issue**: Brief description of measures taken to minimize animal suffering is missing.
   - **Suggestion**: Include a brief description of any measures taken to minimize animal suffering.

**Statistical Analysis**

6. **Clear Description of Statistical Methods**:
   - **Issue**: The statistical methods are not described in sufficient detail.
     - **Example**: The software or tools used for statistical analysis are not mentioned.
     - **Example**: The rationale for choosing specific statistical tests is not explained.
   - **Suggestion**: Clearly describe the statistical methods used, including any software or tools, and explain the rationale for choosing specific tests.

**Results**

7. **Data Presentation**:
   - **Issue**: The data presentation could be clearer and more concise.
     - **Example**: The results could be summarized in a more straightforward manner.
     - **Example**: The use of figures and tables could be optimized to illustrate key findings.
   - **Suggestion**: Summarize the results in a more straightforward manner and optimize the use of figures and tables.
8. **Interpretation of Results**:
   - **Issue**: The results are not sufficiently discussed in relation to the hypothesis and objectives.
   - **Suggestion**: Provide a more thorough interpretation of the findings, linking them back to the hypothesis and objectives.
9. **Linking Results to Introduction**:
   - **Issue**: The results should be more clearly linked to the introduction.
   - **Suggestion**: Ensure that the flow from the background information to the experimental design and findings is seamless.

**Discussion**

10. **Comprehensive Discussion**:
    - **Issue**: The discussion section should comprehensively interpret the results, compare them with existing literature, and discuss the broader implications of the findings.
    - **Suggestion**: Expand the discussion to include comparisons with existing literature and broader implications.
11. **Future Directions**:
    - **Issue**: Potential future research directions are not clearly outlined.
    - **Suggestion**: Clearly outline potential future research directions based on the findings to help understand the broader impact and next steps for the research.

**Grammar and Syntax**

12. **Proofreading**:
    - **Issue**: There are grammatical errors and awkward phrasing.
    - **Suggestion**: Proofread for grammatical errors and awkward phrasing to maintain a professional tone and ensure that the text is polished.
